# Supplementary material for: A Structure—Activity Relationship Study of Bis-Benzamides as Inhibitors of Androgen Receptor—Coactivator Interaction
Source: Molecules. 2019 Jul 31;24(15):2783. doi: 10.3390/molecules24152783 (PMC6696232; doi:10.3390/molecules24152783)
Supplement: Supplementary file 1 [file molecules-24-02783-s001.pdf]

Supplementary Materials

A structure–activity relationship study of bis-benzamides as inhibitors of androgen receptor–coactivator interaction

Tae-Kyung Lee [1], Preethi Ravindranathan [2], Rajni Sonavane [2], Ganesh V. Raj [2] and Jung-Mo Ahn [1,*]

Department of Chemistry and Biochemistry, University of Texas at Dallas, Richardson, TX 75080

Correspondence: jungmo.ahn@utdallas.edu

Abbreviations: 2

Synthesis of compounds **1b**, **6b**, and **10**: 2

Synthesis of bis-benzamide library **14**: 4

Docking simulations using different grid box sizes 7

Cluster analysis on the docked conformers 8

^{1}H NMR and ^{13}C NMR spectra: 11

HPLC chromatograms of bis-benzamide library **14**: 49

References: 52

Abbreviations

ACN, acetonitrile; ADT, androgen deprivation therapy; AF-2, activation function-2; AR, androgen receptor; ARE, androgen response element; Boc, *tert*-butoxycarbonyl; $CDCl_3$, deuterated chloroform; CHCA, α-cyano-4-hydroxycinnamic acid; CRPC, castration-resistant prostate cancer; DIEA, N,N-diisopropylethylamine; DMF, N,N-dimethylformamide; DCM, dichloromethane; DHT, 5-α-dihydrotestosterone; DMSO-d6, deuterated dimethyl sulfoxide; EtOAc, ethyl acetate; ESI, electrospray ionization; Fmoc, 9-fluorenylmethoxycarbonyl; HATU, 2-(7-aza-1H-benzotriazol-1-yl)-1,1,3,3-tetramethyluronium hexafluorophosphate; HOAt, 1-hydroxy-7-azabenzotriazole; HPLC, high performance liquid chromatography; IC_{50}, half-maximal inhibitory concentration; HRMS, high resolution mass spectrometry; LBD, ligand-binding domain; MALDI-TOF MS, matrix-assisted laser desorption/ionization time-of flight mass spectrometry; MTT, 3-(4,5-dimethylthiazol-2-yl)-2,5-diphenyltetrazolium bromide; NMR, nuclear magnetic resonance; PCa, prostate cancer; PELP1, proline-, glutamic acid-and leucine-rich-protein-1; PyBOP, (benzotriazol-1-yloxy)tripyrrolidinophosphonium hexafluorophosphate; TFA, trifluoroacetic acid; THF, tetrahydrofuran; TLC, thin-layer chromatography.

Synthesis of compounds **1b**, **6b**, and **10**

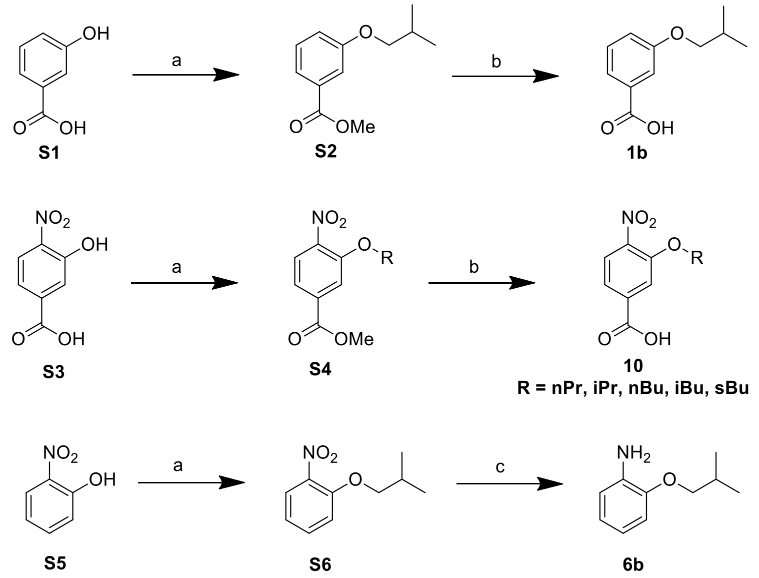

Scheme S1. Synthesis of compounds **1b**, **6b**, and **10**. Reagents and conditions: (a) R-Br, K_2CO_3, DMF, 90 °C, 12 h; (b) NaOH, MeOH/THF, rt, 12 h; (c) $SnCl_2{\cdot}2H_2O$, DMF, rt, 24 h.

General procedure for the synthesis of carboxylic acids **1b**, and **10**

To a solution of phenol **S1**, **S3**, or **S5** (1.0 equiv.) in DMF (20 mL per 1.0 mmol of phenol) was added K_2CO_3 (2 equiv.), followed by R-Br (3.0 equiv.). The reaction mixture was stirred at 90 °C for 12 h and cooled to room temperature. The mixture was concentrated under reduced pressure, and diluted with EtOAc and brine (50 mL). The layers were separated, and the aqueous layer was extracted with EtOAc. The organic layers were combined, washed with brine, dried over anhydrous sodium sulfate, and concentrated under reduced pressure affording O-alkylated compound **S2**, **S4**, or **S6**. Then, to a solution

of the O-alkylated compound **S2** or **S4** in MeOH/THF (1:1, 20 mL per 1.0 mmol of the O-alkylated compound) was added 10% aqueous NaOH solution (5 equiv.) and the resulting solution was stirred at room temperature for 12 h. The solution was then concentrated under reduced pressure, acidified to pH 1-2 with 1N HCl, and extracted with EtOAc (×2). The organic layers were combined, washed with brine, dried over anhydrous sodium sulfate, and concentrated under reduced pressure to afford carboxylic acids **1b** or **10**.

Synthesis of aromatic amine **6b**

To a solution of the O-alkylated compound **S6** (1.0 equiv.) in DMF (10 mL per 1.0 mmol of the O-alkylated compounds) was added $SnCl_2 \cdot 2H_2O$ (5 equiv.) and the resulting solution was stirred at room temperature for 24 h. The solution was then diluted with EtOAc and 1N HCl. The layers were separated, and the aqueous layer was extracted with EtOAc. The organic layers were combined, washed with saturated 1N HCl and brine, dried over anhydrous sodium sulfate, and concentrated under reduced pressure. The crude product was purified by flash column chromatography to afford aromatic amine **6b**.

3-Isobutoxybenzoic acid (1b)

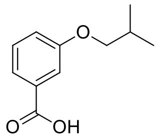

Yellow solid, 98% yield. ^{1}H NMR (DMSO-d_6, 600 MHz): δ 12.96 (br s, 1 H), 7.51 (d, J = 7.8 Hz, 1 H), 7.42 (br s, 1 H), 7.39 (t, J = 7.9 Hz, 1 H), 7.18 (dd, J = 8.1, 1.8 Hz, 1 H), 3.79 (d, J = 6.6 Hz, 2 H), 2.05 – 1.99 (m, 1 H), 0.98 (d, J = 6.6 Hz, 6 H). ^{13}C NMR (DMSO-d_6, 150 MHz): δ 167.1, 158.8, 132.2, 129.7, 121.4, 119.3, 114.5, 73.9, 27.7, 19.0.

4-Nitro-3-propoxybenzoic aid (10-nPr)

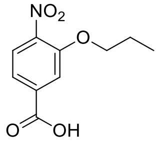

Light yellow solid, 86% yield. ^{1}H NMR (DMSO-d_6, 270 MHz): δ 7.95 (d, J = 8.4 Hz, 1 H), 7.74 (d, J = 1.5 Hz, 1 H), 7.63 (dd, J = 8.4, 1.5 Hz, 1 H), 4.18 (t, J = 6.3 Hz, 2 H), 1.80 – 1.67 (m, 2 H), 0.97 (t, J = 7.3 Hz, 3 H). ^{13}C NMR (DMSO-d_6, 150 MHz): δ 165.8, 150.9, 142.1, 135.6, 124.9, 121.2, 115.3, 70.8, 21.8, 10.1.

3-Isopropoxy-4-nitrobenzoic aid (10-iPr)

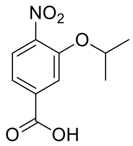

The title compound was synthesized as previously described [1].

3-Butoxy-4-nitrobenzoic aid (10-nBu)

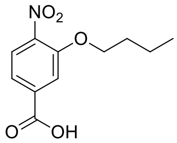

The title compound was synthesized as previously described [1].

3-Isobutoxy-4-nitrobenzoic aid (10-iBu)

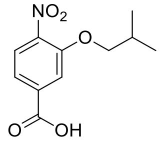

The title compound was synthesized as previously described [2].

3-*sec*-Butoxy-4-nitrobenzoic aid (10-sBu)

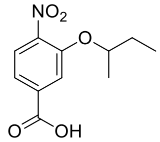

Light yellow solid, 92% yield. ^{1}H NMR (DMSO-d_6, 600 MHz): δ 13.60 (br s, 1 H), 7.92 (d, J = 8.4 Hz, 1 H), 7.75 (br s, 1 H), 7.61 (dd, J = 8.4, 0.7 Hz, 1 H), 4.73 – 4.68 (m, 1 H), 1.68 – 1.61(m, 2 H), 1.27 (d, J = 6.2 Hz, 3 H), 0.91 (t, J = 7.5 Hz, 3 H). ^{13}C NMR (DMSO-d_6, 150 MHz): δ 165.8, 149.8, 143.2, 135.4, 124.8, 121.1, 116.3, 76.9, 28.4, 18.6, 9.1.

2-Isobutoxyaniline (6b)

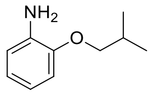

Light yellow oil, 46% yield. ^{1}H NMR (DMSO-d_6, 600 MHz): δ 6.74 (d, J = 8.1 Hz, 1 H), 6.67 – 6.63 (m, 2 H), 6.50 (t, J = 7.0 Hz, 1 H), 4.69 (br s, 2 H), 3.69 (d, J = 6.2 Hz, 2 H), 2.06 – 1.99 (m, 1 H), 0.99 (d, J = 6.6 Hz, 6 H). ^{13}C NMR (DMSO-d_6, 150 MHz): δ 145.8, 137.4, 120.7, 116.4, 114.0, 111.5, 73.9, 27.8, 19.1.

Bis-benzamide library **14**

4-[(3-*sec*-Butoxy-4-nitrobenzoyl)amino]-3-propoxybenzamide (14e)

Light yellow solid, 25 mg, 40% overall yield, 96% purity by HPLC. ^{1}H NMR (DMSO-d_6, 270 MHz): δ 9.74 (br s, 1 H), 7.98 (br s, 1 H), 7.97 (d, J = 8.2 Hz, 1 H), 7.87 (d, J = 8.4 Hz, 1 H), 7.81 (d, J = 1.4 Hz, 1 H), 7.582 (dd, J = 8.2, 1.7 Hz, 1 H), 7.576 (d, J = 1.7 Hz, 1 H), 7.53 (dd, J = 8.3, 1.4 Hz, 1 H), 7.37 (br s, 1 H), 4.81 – 4.70 (m, 1 H), 4.05 (t, J = 6.4 Hz, 2 H), 1.84 – 1.73 (m, 2 H), 1.73 – 1.63 (m, 2 H), 1.30 (d, J = 6.2 Hz, 3 H), 0.99 (t, J = 7.4 Hz, 3 H), 0.92 (t, J = 7.4 Hz, 3 H). ^{13}C NMR (DMSO-d_6, 150 MHz): δ 167.2, 163.6, 150.4, 150.0, 142.2, 139.2, 131.7, 129.2, 124.9, 123.3, 119.8, 119.5, 114.8, 111.4, 77.0, 69.9, 28.4, 22.0, 18.7, 10.4, 9.2. MALDI-TOF (*m*/*z*): $[M+Na]^+$ calcd for $C_{21}H_{25}N_3NaO_6$: 438.16, found 438.95.

3-Isopropoxy-4-[(3-isopropoxy-4-nitrobenzoyl)amino]benzamide (14g)

Light yellow solid, 27 mg, 45% overall yield, > 99% purity by HPLC. ^{1}H NMR (DMSO-d_6, 270 MHz): δ 9.63 (br s, 1 H), 7.99 (br s, 1 H), 7.97 (d, J = 8.4 Hz, 1 H), 7.94 (d, J = 8.4 Hz, 1 H), 7.80 (d, J = 1.2 Hz, 1 H), 7.581 (d, J = 1.7 Hz, 1 H), 7.575 (dd, J = 8.2, 1.2 Hz, 1 H), 7.52 (dd, J = 8.3, 1.6 Hz, 1 H), 7.37 (br s, 1 H), 4.95 (sep, J = 5.9 Hz, 1 H), 4.70 (sep, J = 5.9 Hz, 1 H), 1.34 (d, J = 5.9 Hz, 6 H), 1.32 (d, J = 5.9 Hz, 6 H). ^{13}C NMR (DMSO-d_6, 150 MHz): δ 167.2, 163.6, 149.8, 148.8, 142.3, 139.3, 131.4, 130.3, 125.0, 122.9, 119.9, 119.5, 115.1, 113.0, 72.5, 71.2, 21.8, 21.6. MALDI-TOF (*m*/*z*): $[M+Na]^+$ calcd for $C_{20}H_{23}N_3NaO_6$: 424.15, found 424.99.

4-[(3-*sec*-Butoxy-4-nitrobenzoyl)amino]-3-isopropoxybenzamide (14j)
Light yellow solid, 25 mg, 40% overall yield, 95% purity by HPLC. ^{1}H NMR (DMSO-d_6, 270 MHz): δ 9.63 (br s, 1 H), 7.99 (br s, 1 H), 7.97 (d, *J* = 8.2 Hz, 1 H), 7.95 (d, *J* = 8.3 Hz, 1 H), 7.78 (br s, 1 H), 7.58 (br s, 1 H), 7.56 (dd, *J* = 8.4, 1.5 Hz, 1 H), 7.52 (dd, *J* = 8.3, 1.5 Hz, 1 H), 7.37 (br s, 1 H), 4.79 – 4.66 (m, 2 H), 1.73 – 1.63 (m, 2 H), 1.32 (d, *J* = 5.9 Hz, 6 H), 1.31 (d, *J* = 5.9 Hz, 3 H), 0.93 (t, *J* = 7.4 Hz, 3 H). ^{13}C NMR (DMSO-d_6, 150 MHz): δ 167.2, 163.6, 150.0, 148.8, 142.2, 139.3, 131.4, 130.3, 125.0, 122.9, 119.9, 119.4, 114.9, 113.0, 77.0, 71.2, 28.4, 21.8, 18.7, 9.2. MALDI-TOF (*m*/*z*): [M+Na]$^+$ calcd for $C_{21}H_{25}N_3NaO_6$: 438.16, found 438.96.

3-Butoxy-4-[(3-isobutoxy-4-nitrobenzoyl)amino]benzamide (14n)
Light yellow solid, 28 mg, 44% overall yield, 92% purity by HPLC. ^{1}H NMR (DMSO-d_6, 270 MHz): δ 9.74 (br s, 1 H), 8.01 (d, *J* = 8.4 Hz, 1 H), 7.99 (br s, 1 H), 7.85 (d, *J* = 8.4 Hz, 1 H), 7.79 (br s, 1 H), 7.60 (d, *J* = 8.2 Hz, 1 H), 7.58 (br s, 1 H), 7.53 (d, *J* = 8.4 Hz, 1 H), 7.37 (br s, 1 H), 4.09 (t, *J* = 6.4 Hz, 2 H), 4.03 (d, *J* = 6.4 Hz, 1 H), 2.14 – 2.02 (m, 1 H), 1.80 – 1.70 (m, 2 H), 1.52 – 1.39 (m, 2 H), 0.99 (d, *J* = 6.7 Hz, 6 H), 0.91 (t, *J* = 7.2 Hz, 3 H). ^{13}C NMR (DMSO-d_6, 150 MHz): δ 167.2, 163.5, 151.0, 150.4, 141.1, 139.4, 131.8, 129.2, 125.0, 123.4, 119.8, 119.5, 113.9, 111.4, 75.2, 68.1, 30.6, 27.6, 18.7, 18.7, 13.7. MALDI-TOF (*m*/*z*): [M+Na]$^+$ calcd for $C_{22}H_{27}N_3NaO_6$: 452.18, found 452.83.

3-Isobutoxy-4-[(4-nitro-3-propoxybenzoyl)amino]benzamide (14p)
Light yellow solid, 25 mg, 40% overall yield based on the loading of Fmoc-Rink amide resin, > 99% purity by HPLC, m.p. 258–260 °C. ^{1}H NMR (DMSO-d_6, 270 MHz): δ 9.77 (br s, 1 H), 8.01 (d, *J* = 8.2 Hz, 1 H), 8.00 (br s, 1 H), 7.83 (d, *J* = 8.2 Hz, 1 H), 7.80 (d, *J* = 1.5 Hz, 1 H), 7.61 (dd, *J* = 8.4, 1.5 Hz, 1 H), 7.57 (d, *J* = 1.7 Hz, 1 H), 7.53 (dd, *J* = 8.2, 1.7 Hz, 1 H), 7.37 (br s, 1 H), 4.21 (t, *J* = 6.4 Hz, 2 H), 3.87 (d, *J* = 6.4 Hz, 2 H), 2.14 – 2.00 (m, 1 H), 1.83 – 1.70 (m, 2 H), 0.99 (d, *J* = 6.7 Hz, 6 H), 0.98 (t, *J* = 7.4 Hz, 3 H). ^{13}C NMR (DMSO-d_6, 150 MHz): δ 167.2, 163.5, 150.9, 150.7, 141.2, 139.4, 131.9, 129.2, 125.0, 123.6, 119.7, 119.5, 113.9, 111.4, 74.5, 70.8, 27.8, 21.7, 19.0, 10.1. MALDI-TOF (*m*/*z*): [M+H]$^+$ calcd for $C_{21}H_{26}N_3O_6$: 416.18, found 416.57.

3-Isobutoxy-4-[(3-isopropoxy-4-nitrobenzoyl)amino]benzamide (14q)
Light yellow solid, 21 mg, 32% overall yield, > 99% purity by HPLC. ^{1}H NMR (DMSO-d_6, 270 MHz): δ 9.76 (br s, 1 H), 7.99 (br s, 1 H), 7.97 (d, *J* = 8.4 Hz, 1 H), 7.83 (d, *J* = 8.2 Hz, 1 H), 7.82 (d, *J* = 1.5 Hz, 1 H), 7.59 (dd, *J* = 8.4, 1.5 Hz, 1 H), 7.57 (br s, 1 H), 7.53 (dd, *J* = 8.2, 1.7 Hz, 1 H), 7.37 (br s, 1 H), 4.93 (sep, *J* = 5.9 Hz, 1 H), 3.86 (d, *J* = 6.4 Hz, 2 H), 2.14 – 1.99 (m, 1 H), 1.33 (d, *J* = 6.2 Hz, 6 H), 0.99 (d, *J* = 6.7 Hz, 6 H). ^{13}C NMR (DMSO-d_6, 150 MHz): δ 167.2, 163.5, 150.7, 149.7, 142.3, 139.2, 131.9, 129.2, 124.9, 123.7, 119.7, 119.6, 115.0, 111.4, 74.5, 72.5, 27.8, 21.6, 19.1. MALDI-TOF (*m*/*z*): [M+Na]$^+$ calcd for $C_{21}H_{25}N_3NaO_6$: 438.16, found 438.98.

3-*sec*-Butoxy-4-[(4-nitro-3-propoxybenzoyl)amino]benzamide (14t)
Light yellow solid, 28 mg, 45% overall yield, 93% purity by HPLC. ^{1}H NMR (DMSO-d_6, 270 MHz): δ 9.66 (br s, 1 H), 8.01 (d, *J* = 8.4 Hz, 1 H), 8.00 (br s, 1 H), 7.90 (d, *J* = 8.2 Hz, 1 H), 7.77 (d, *J* = 1.4 Hz, 1 H), 7.59 (dd, *J* = 8.4, 1.5 Hz, 1 H), 7.57 (d, *J* = 1.5 Hz, 1 H), 7.52 (dd, *J* = 8.3, 1.5 Hz, 1 H), 7.37 (br s, 1 H), 4.56 – 4.46 (m, 1 H), 4.22 (t, *J* = 6.4 Hz, 2 H), 1.84 – 1.60 (m, 4 H), 1.27 (d, *J* = 6.2 Hz, 3 H), 0.98 (t, *J* = 7.4 Hz, 3 H), 0.93 (t, *J* = 7.4 Hz, 3 H). ^{13}C NMR (DMSO-d_6, 150 MHz): δ 167.2, 163.5, 150.9, 149.2, 141.2, 139.4, 131.6, 130.2, 125.1, 123.2, 119.8, 119.4, 114.0, 112.9, 75.9, 70.8, 28.6, 21.7, 18.8, 10.2, 9.4. MALDI-TOF (*m*/*z*): [M+Na]$^+$ calcd for $C_{21}H_{25}N_3NaO_6$: 438.16, found 438.99.

3-*sec*-Butoxy-4-[(3-isopropoxy-4-nitrobenzoyl)amino]benzamide (14u)
Light yellow solid, 29 mg, 47% overall yield, > 99% purity by HPLC. ^{1}H NMR (DMSO-d_6, 270 MHz): δ 9.65 (br s, 1 H), 7.99 (br s, 1 H), 7.97 (d, *J* = 8.4 Hz, 1 H), 7.92 (d, *J* = 8.2 Hz, 1 H), 7.79 (d, *J* = 1.5 Hz, 1 H), 7.572

(d, *J* = 1.7 Hz, 1 H), 7.567 (dd, *J* = 8.3, 1.5 Hz, 1 H), 7.52 (dd, *J* = 8.4, 1.7 Hz, 1 H), 7.37 (br s, 1 H), 4.94 (sep, *J* = 5.9 Hz, 1 H), 4.54 – 4.46 (m, 1 H), 1.77 – 1.58 (m, 2 H), 1.33 (d, *J* = 5.9 Hz, 6 H), 1.26 (d, *J* = 5.9 Hz, 3 H), 0.93 (t, *J* = 7.4 Hz, 3 H). ^{13}C NMR (DMSO-d_6, 150 MHz): δ 167.2, 163.6, 149.7, 149.2, 142.3, 139.3, 131.6, 130.2, 125.0, 123.1, 119.8, 119.5, 115.1, 112.9, 75.9, 72.5, 28.6, 21.6, 18.8, 9.4. MALDI-TOF (*m*/*z*): [M+Na]$^+$ calcd for $C_{21}H_{25}N_3NaO_6$: 438.16, found 438.96.

3-sec-Butoxy-4-[(3-butoxy-4-nitrobenzoyl)amino]benzamide (14v)

Light yellow solid, 25 mg, 39% overall yield based on the loading of Fmoc-Rink amide resin, > 99% purity by HPLC. ^{1}H NMR (DMSO-d_6, 270 MHz): δ 9.66 (br s, 1 H), 8.01 (d, *J* = 8.2 Hz, 1 H), 7.99 (br s, 1 H), 7.90 (d, *J* = 8.2 Hz, 1 H), 7.78 (d, *J* = 1.2 Hz, 1 H), 7.59 (dd, *J* = 8.2, 1.5 Hz, 1 H), 7.57 (d, *J* = 1.2 Hz, 1 H), 7.52 (dd, *J* = 8.3, 1.6 Hz, 1 H), 7.37 (br s, 1 H), 4.56 – 4.46 (m, 1 H), 4.26 (t, *J* = 6.2 Hz, 2 H), 1.79 – 1.61 (m, 4 H), 1.51 – 1.37 (m, 2 H), 1.26 (d, *J* = 5.9 Hz, 3 H), 0.933 (t, *J* = 7.4 Hz, 3 H), 0.929 (t, *J* = 7.4 Hz, 3 H). ^{13}C NMR (DMSO-d_6, 150 MHz): δ 167.2, 163.5, 150.9, 149.2, 141.2, 139.4, 131.6, 130.2, 125.0, 123.1, 119.8, 119.4, 114.0, 112.9, 75.9, 69.1, 30.3, 28.5, 18.8, 18.5, 13.5, 9.4. MALDI-TOF (*m*/*z*): [M+H]$^+$ calcd for $C_{22}H_{28}N_3O_6$: 430.20, found 430.55.

3-sec-Butoxy-4-[(3-isobutoxy-4-nitrobenzoyl)amino]benzamide (14w)

Light yellow solid, 24 mg, 37% overall yield based on the loading of Fmoc-Rink amide resin, > 99% purity by HPLC, m.p. 204–206 °C. ^{1}H NMR (DMSO-d_6, 270 MHz): δ 9.66 (br s, 1 H), 8.02 (d, *J* = 8.4 Hz, 1 H), 7.99 (br s, 1 H), 7.91 (d, *J* = 8.4 Hz, 1 H), 7.76 (d, *J* = 1.2 Hz, 1 H), 7.59 (dd, *J* = 8.3, 1.5 Hz, 1 H), 7.57 (d, *J* = 1.2 Hz, 1 H), 7.52 (dd, *J* = 8.4, 1.5 Hz, 1 H), 7.37 (br s, 1 H), 4.57 – 4.45 (m, 1 H), 4.04 (d, *J* = 6.4 Hz, 2 H), 2.13 – 1.99 (m, 1 H), 1.78 – 1.61 (m, 2 H), 1.27 (d, *J* = 6.2 Hz, 3 H), 0.99 (d, *J* = 6.7 Hz, 6 H), 0.93 (t, *J* = 7.4 Hz, 3 H). ^{13}C NMR (DMSO-d_6, 150 MHz): δ 167.2, 163.5, 151.0, 149.2, 141.1, 139.5, 131.6, 130.2, 125.1, 123.1, 119.8, 119.4, 113.9, 112.9, 75.9, 75.2, 28.5, 27.6, 18.8, 18.7, 9.4. MALDI-TOF (*m*/*z*): [M+H]$^+$ calcd for $C_{22}H_{28}N_3O_6$: 430.20, found 430.56.

3-*sec*-Butoxy-4-[(3-sec-butoxy-4-nitrobenzoyl)amino]benzamide (14x)

Light yellow solid, 24 mg, 37% overall yield, 93% purity by HPLC. ^{1}H NMR (DMSO-d_6, 270 MHz): δ 9.64 (br s, 1 H), 7.99 (br s, 1 H), 7.97 (d, *J* = 8.4 Hz, 1 H), 7.92 (d, *J* = 8.2 Hz, 1 H), 7.77 (br s, 1 H), 7.57 (br s, 1 H), 7.56 (dd, *J* = 8.3, 1.5 Hz, 1 H), 7.52 (dd, *J* = 8.4, 1.5 Hz, 1 H), 7.37 (br s, 1 H), 4.80 – 4.69 (m, 1 H), 4.56 – 4.46 (m, 1 H), 1.74 – 1.60 (m, 4 H), 1.30 (d, *J* = 5.9 Hz, 3 H), 1.26 (d, *J* = 5.9 Hz, 3 H), 0.93 (t, *J* = 7.4 Hz, 6 H). ^{13}C NMR (DMSO-d_6, 150 MHz): δ 167.2, 163.6, 150.0, 149.2, 142.2, 139.3, 131.6, 130.2, 125.0, 123.1, 119.8, 119.4, 114.8, 112.9, 77.0, 75.9, 28.6, 28.4, 18.8, 18.7, 9.4, 9.2. MALDI-TOF (*m*/*z*): [M+Na]$^+$ calcd for $C_{22}H_{27}N_3NaO_6$: 452.18, found 453.04.

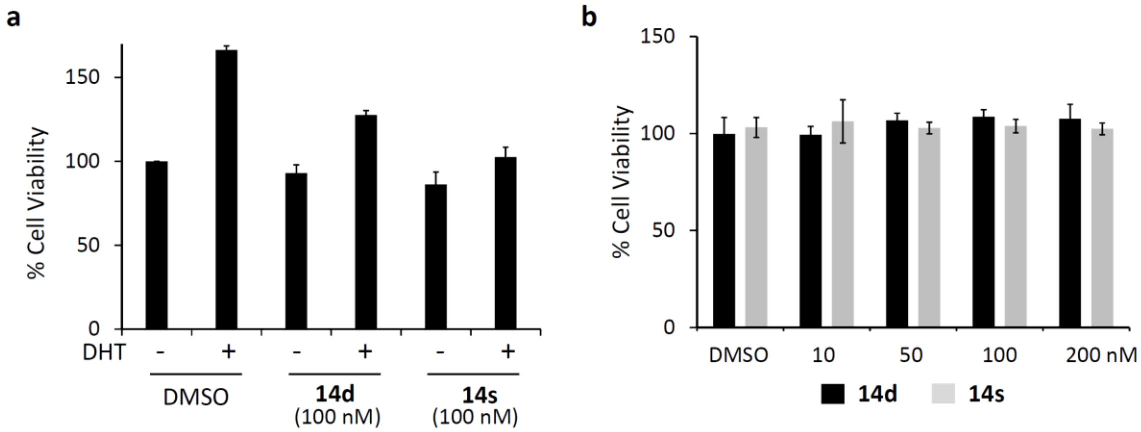

Figure S1. Dependence of compounds **14d** and **14s** on AR-signaling. (**a**) The effects of **14d** and **14s** for the growth inhibition of LNCaP cells in the presence or absence of DHT by MTT assay. (**b**) Dose-response experiments of **14d** and **14s** for the growth inhibition of PC3 cells by MTT assay.

Docking simulations using different grid box sizes

Docking calculations were performed as described in 4.7 Molecular docking study and docking poses with the lowest binding energy are shown in Figure S1.

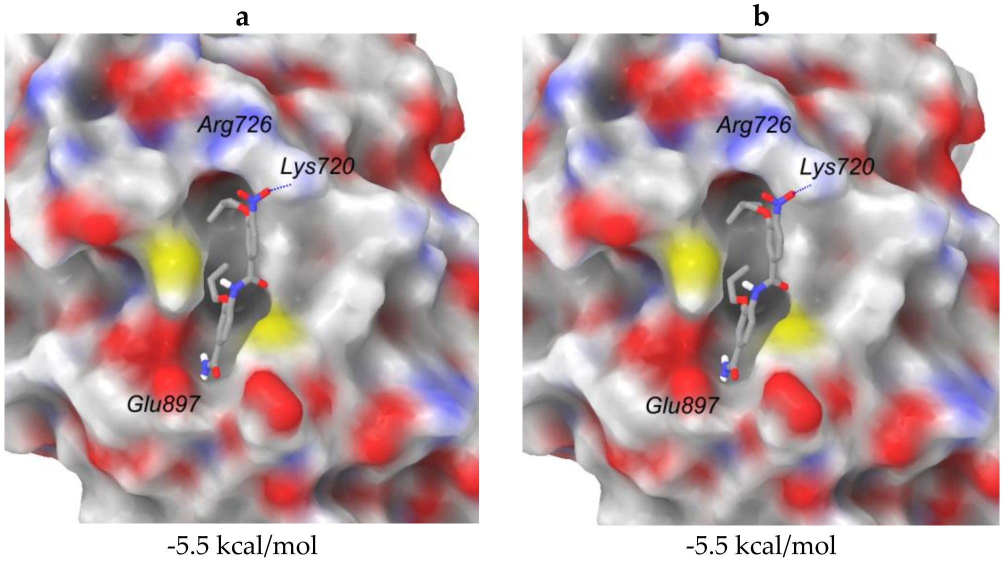

Figure S2. Docked poses for compound **14d** with the lowest binding energy obtained using a grid box of size (a) 22 × 22 × 22 Å and (b) 26 × 26 × 26 Å.

Cluster analysis on the docked conformers

Docking calculations were performed five times and 19 or 20 conformers from each docking were retrieved. The resulting 100 conformers of **14d** and 97 conformers of **D2** were clustered using clustering of conformer's script in Maestro (version 9.1, Schrödinger, LLC, New York, NY, USA, 2010). The clustering was performed with atomic RMSD using the average-linkage method. To generate the RMS matrix, all heavy atoms were included. The docked conformers were energy minimized in the MacroModel suite of Maestro (OPLS-2005 force filed, default settings). The results are shown in Table S1 and S2.

Table S1. Clusters of docked poses of compound **14d** on the AF-2 domain of AR (PDB code 1T63)

Cluster	Mean binding energy of the cluster (kcal/mol)	Number of Conformers	The lowest energy binding mode of the cluster (kcal/mol)	
1	-5.04	39	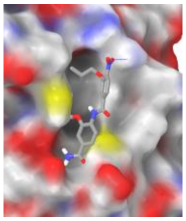	-5.5
2	-4.91	19	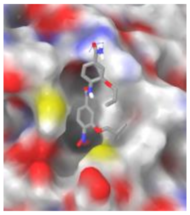	-5.2
3	-4.90	1	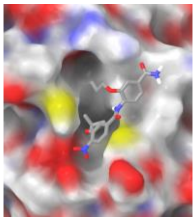	-4.9
4	-4.83	20	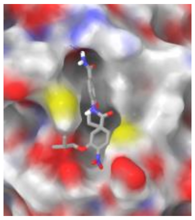	-5.1
5	-4.73	7	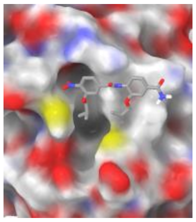	-5.0
6	-4.72	13	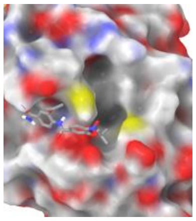	-5.0
7	-4.70	1	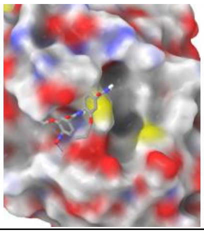	-4.7

Table S2. Clusters of docked poses of compound **D2** on the AF-2 domain of AR (PDB code 1T63)

Cluster	Mean binding energy of the cluster (kcal/mol)	Number of Conformers	The lowest energy binding mode of the cluster (kcal/mol)	
1	-4.06	26	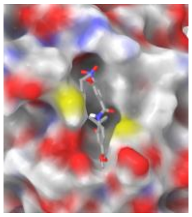	-5.0
2	-3.95	19	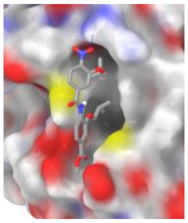	-4.7
3	-3.80	33	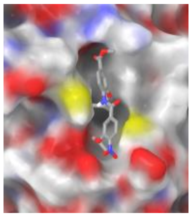	-4.6
4	-3.44	11	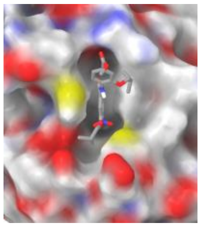	-3.7
5	-3.38	4	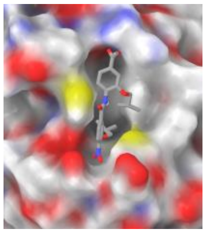	-3.6
6	-3.00	3	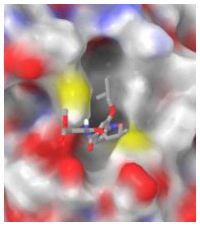	-3.1
7	-2.80	1	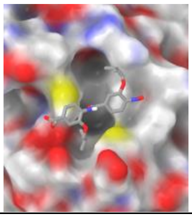	-2.8

^{1}H NMR and ^{13}C NMR spectra

Compound **S3**

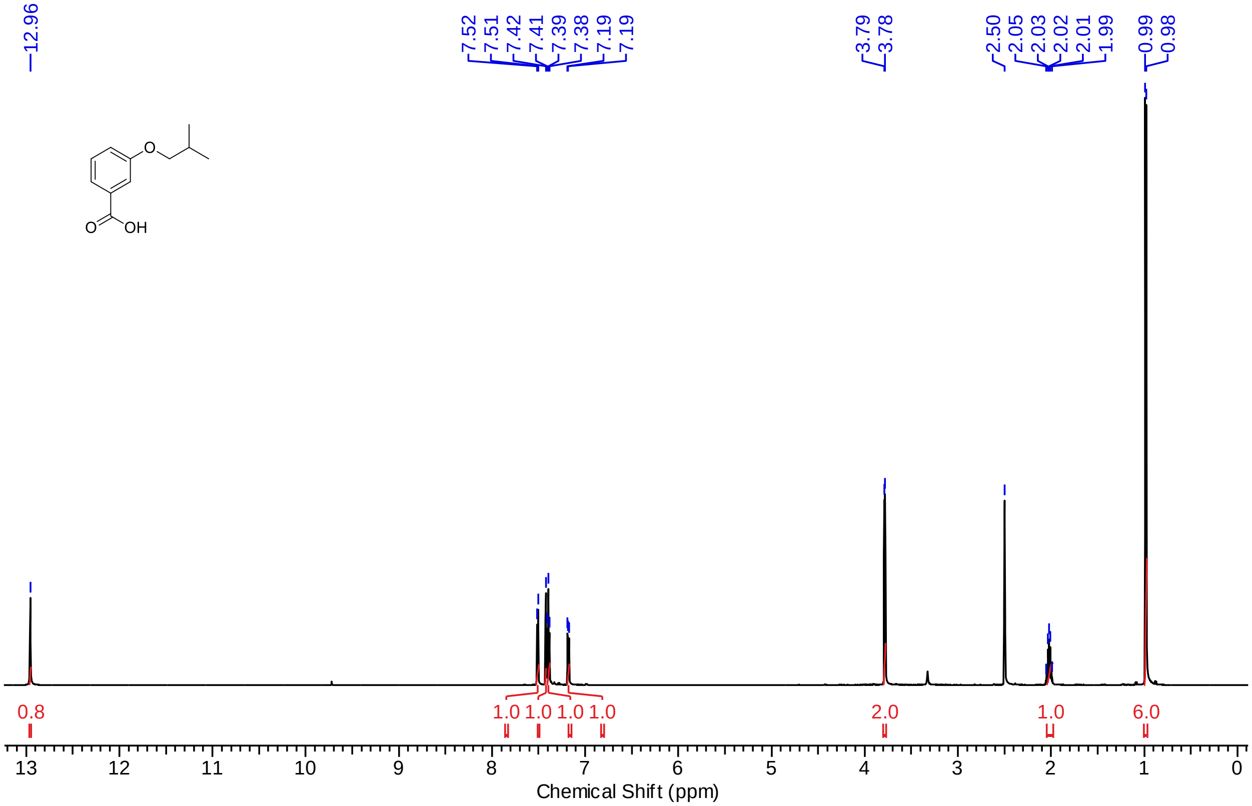

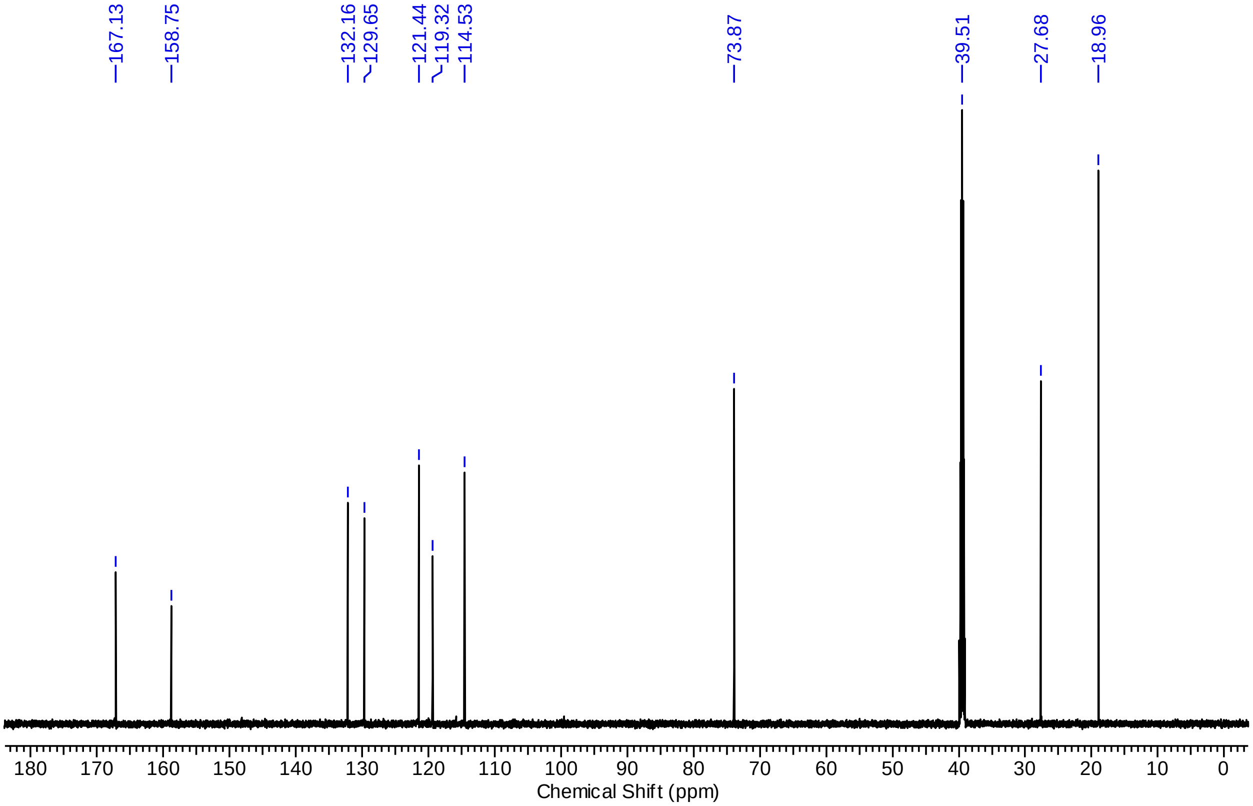

Compound **S6**

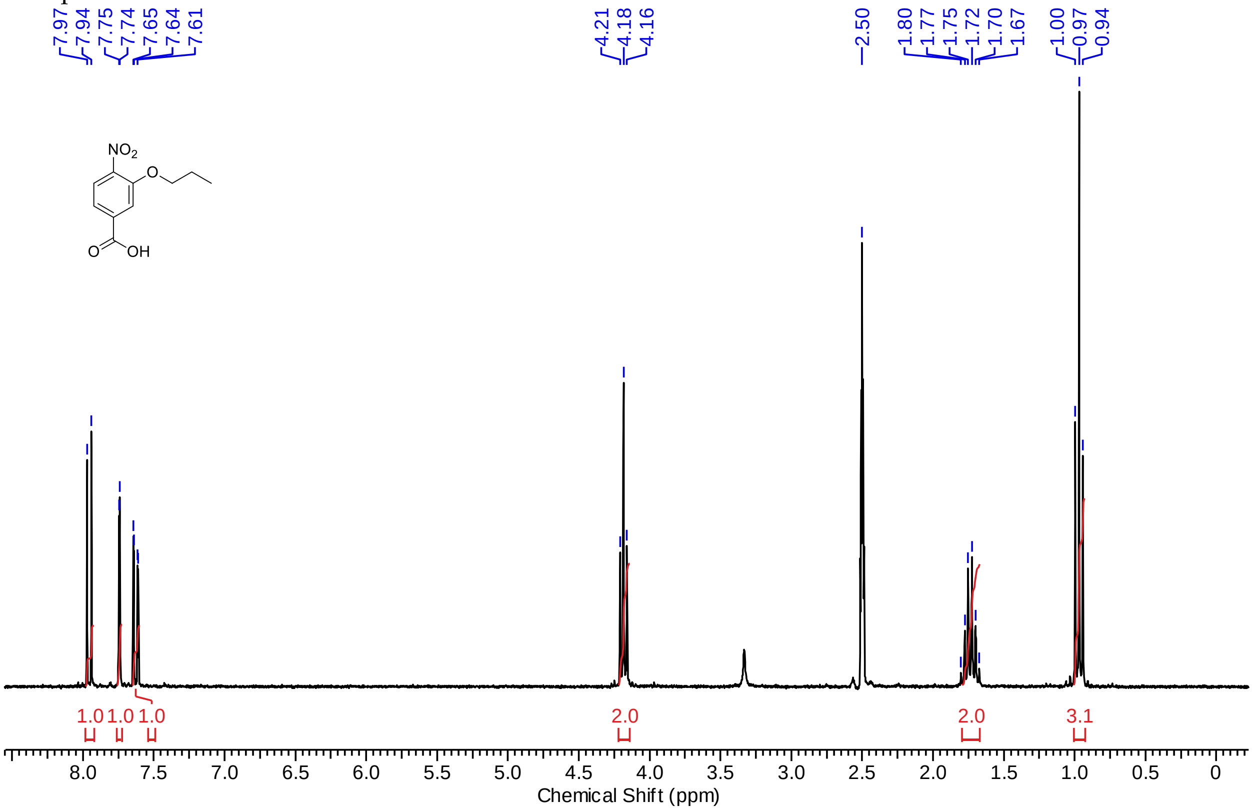
7.97
7.94
7.75
7.74
7.65
7.64
7.61
4.21
4.18
4.16
2.50
1.80
1.77
1.75
1.72
1.70
1.67
1.00
0.97
0.94
NO2
O
O
OH
1.0
1.0
1.0
2.0
2.0
3.1
Chemical Shift (ppm)

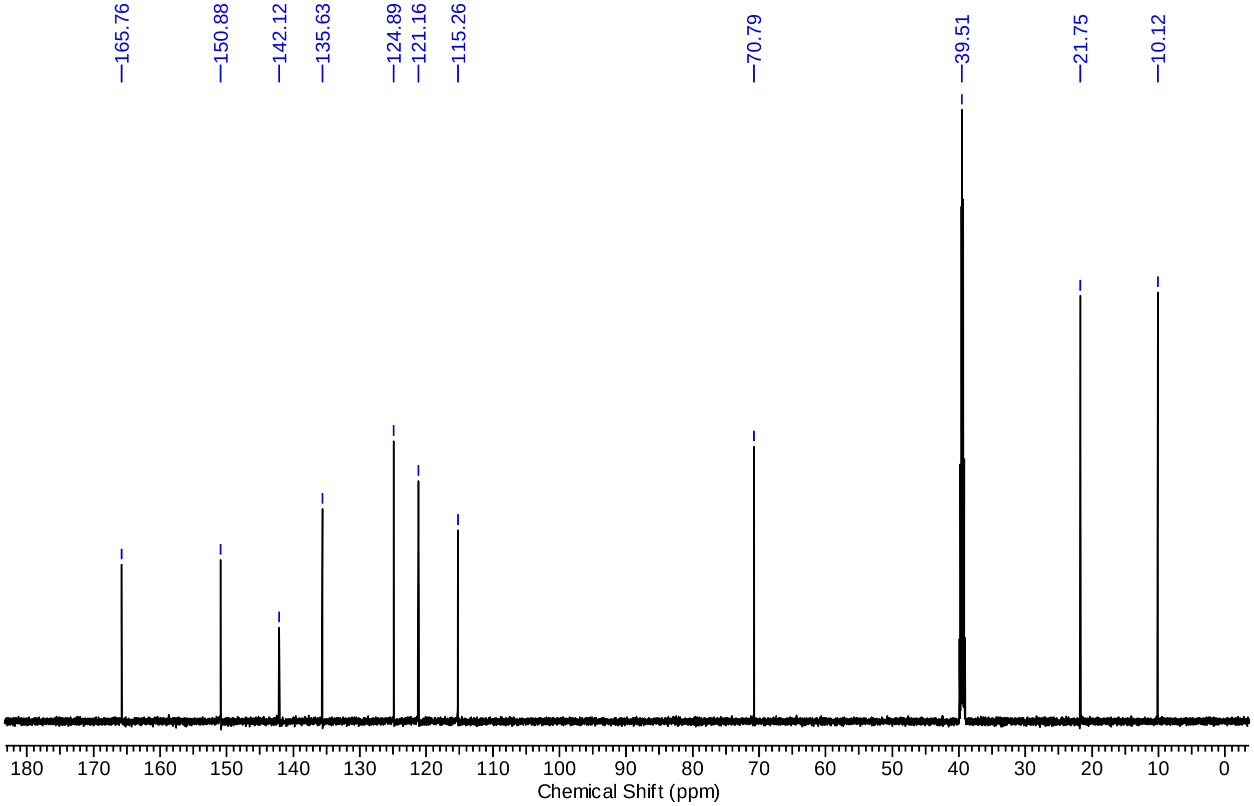
165.76
150.88
142.12
135.63
124.89
121.16
115.26
70.79
39.51
21.75
10.12
Chemical Shift (ppm)

Compound **S10**

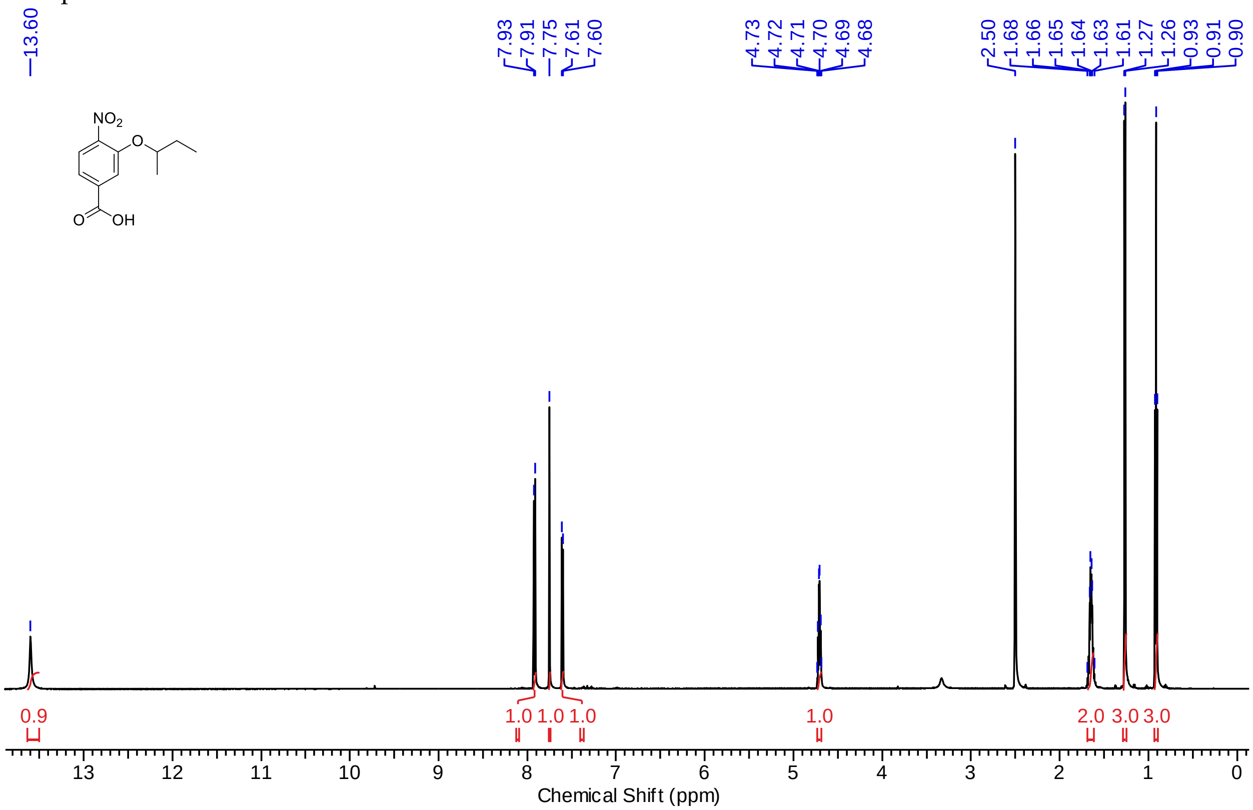

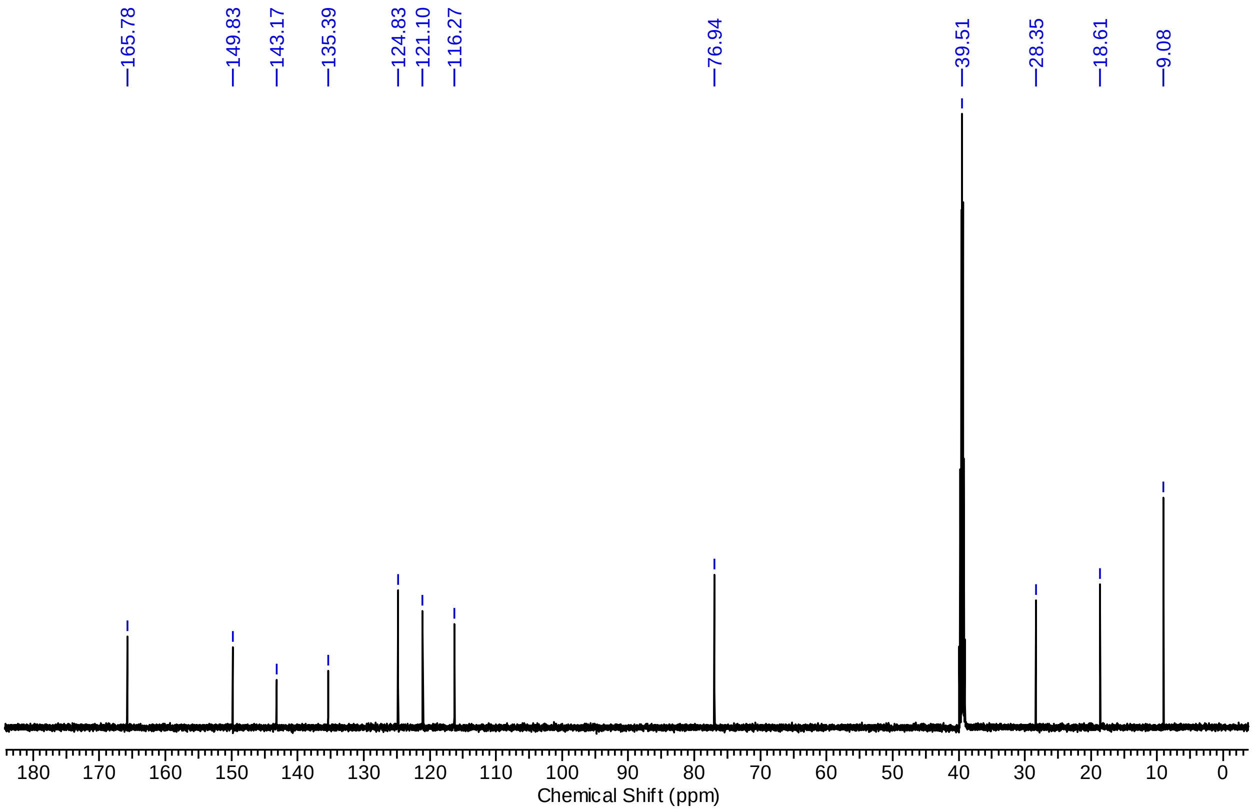

Compound **S13**

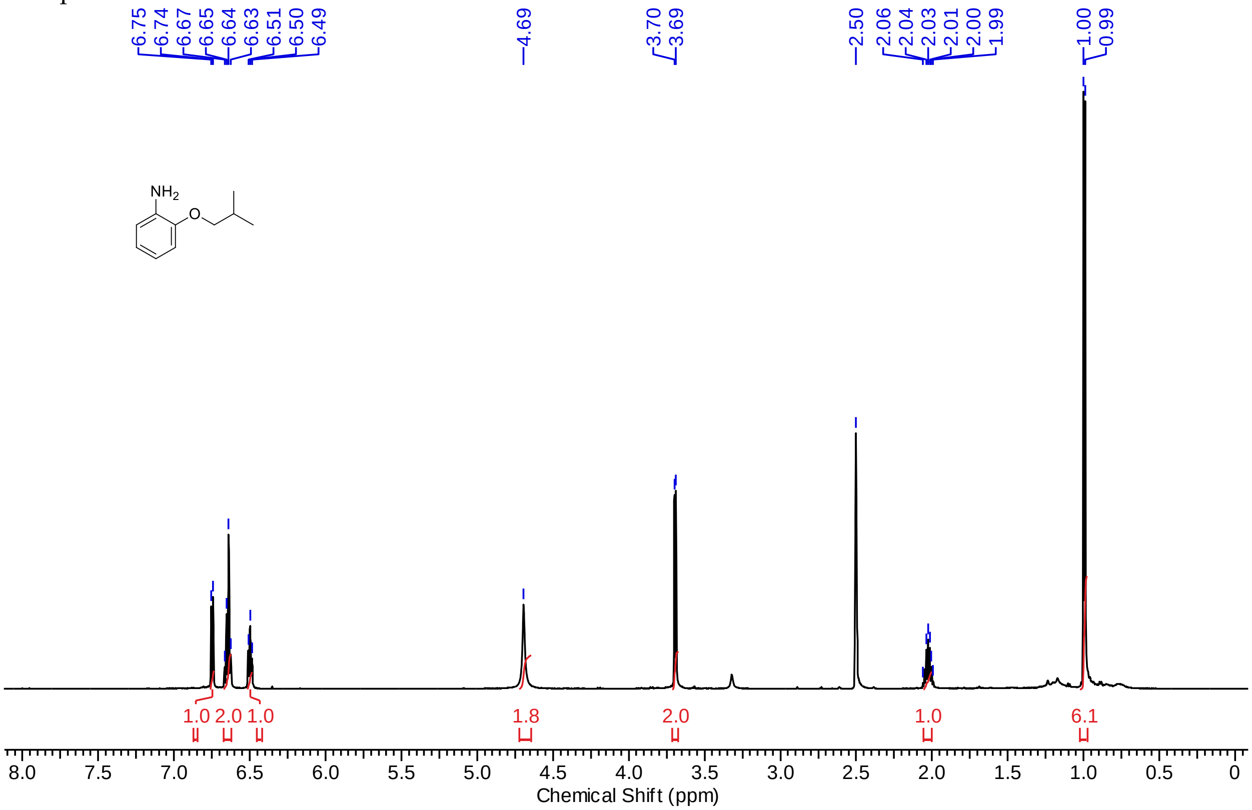

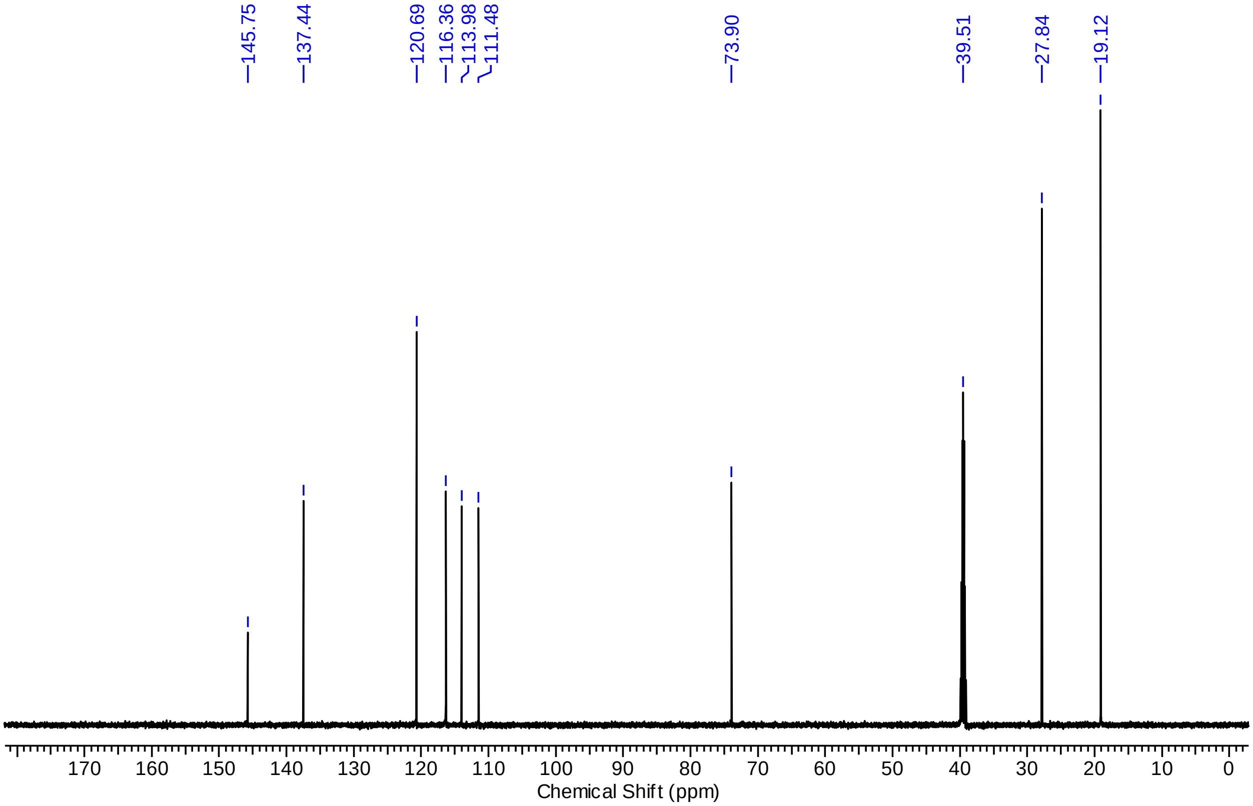

Compound **3**

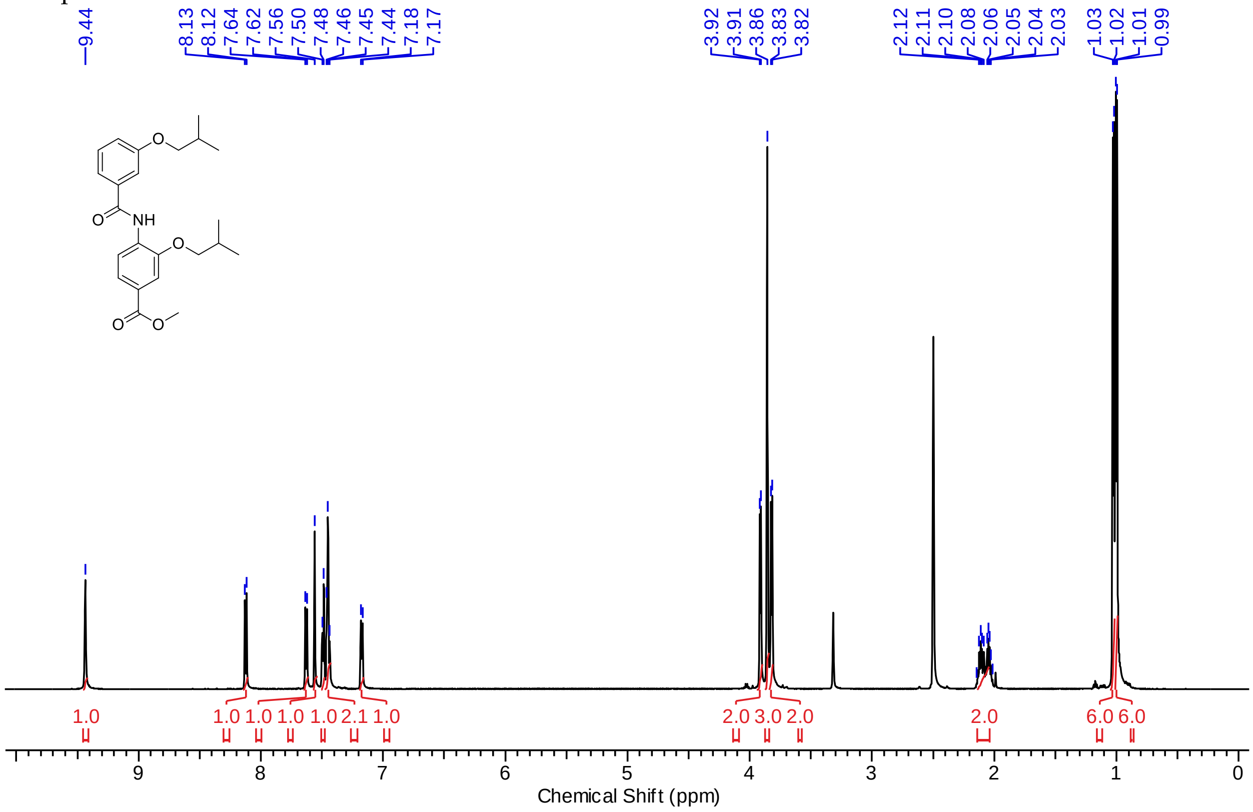

9.44
8.13 8.12 7.64 7.62 7.56 7.50 7.48 7.46 7.45 7.44 7.18 7.17
3.92 3.91 3.86 3.83 3.82
2.12 2.11 2.10 2.08 2.06 2.05 2.04 2.03
1.03 1.02 1.01 0.99
O
NH
O
O
O
1.0 1.0 1.0 1.0 1.0 2.1 1.0
2.0 3.0 2.0
2.0
6.0 6.0
Chemical Shift (ppm)

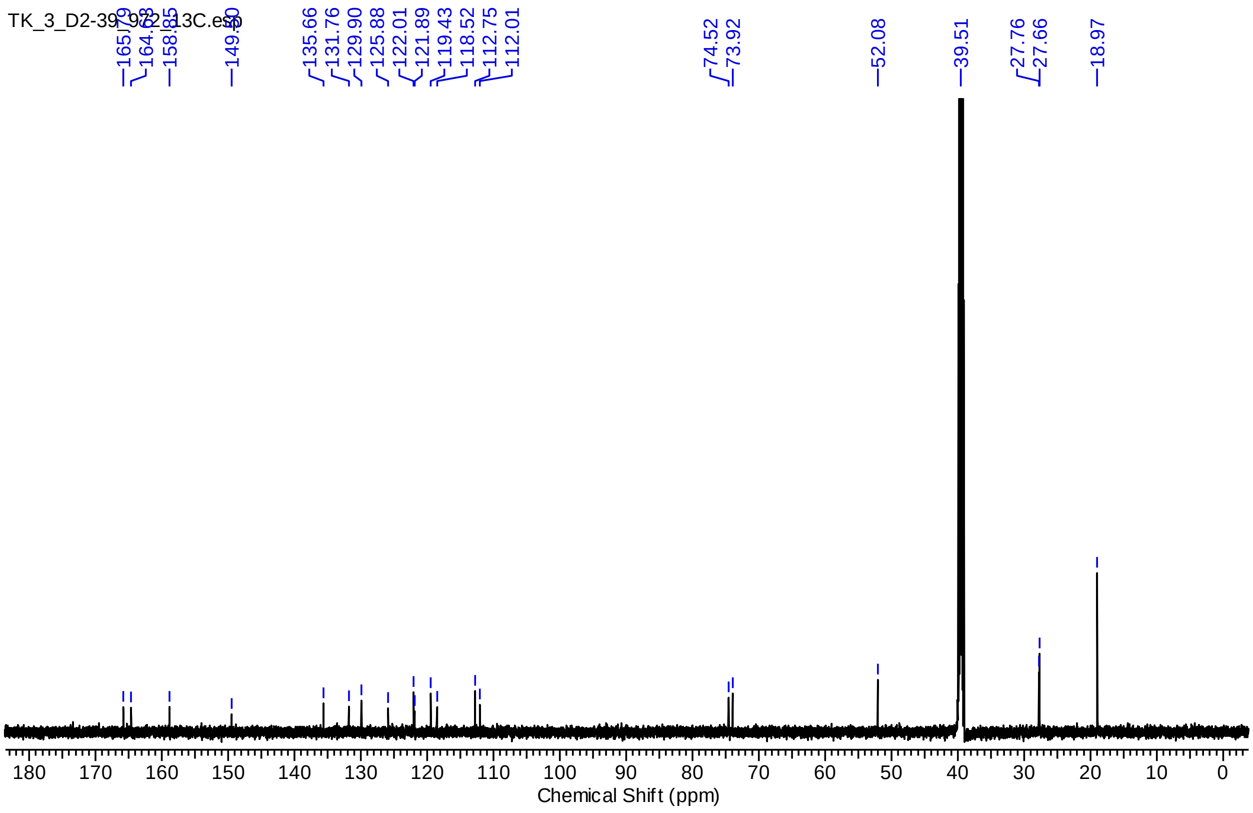

158.85
135.66 131.76 129.90 125.88 122.01 121.89 119.43 118.52 112.75 112.01
74.52 73.92
52.08
39.51
27.76 27.66
18.97
Chemical Shift (ppm)

Compound **5a**

TK_5a_D2-2_695.esp

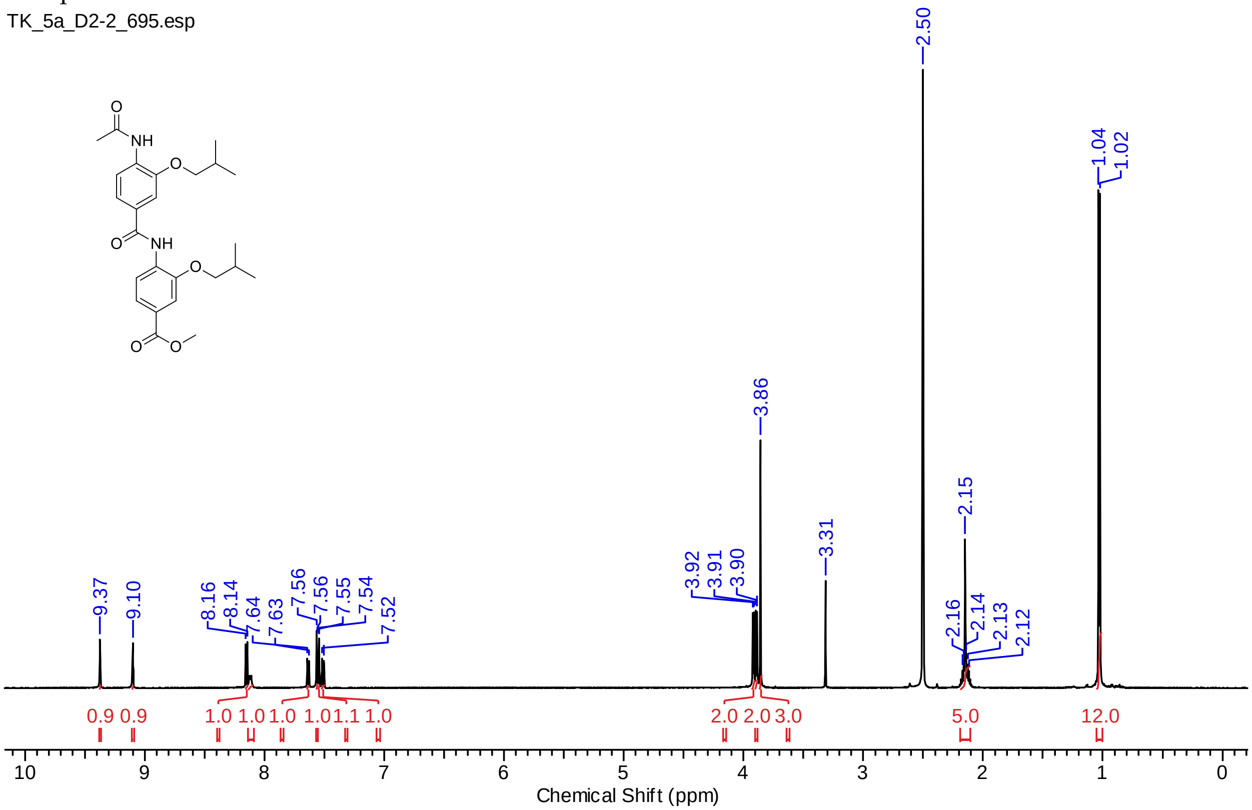

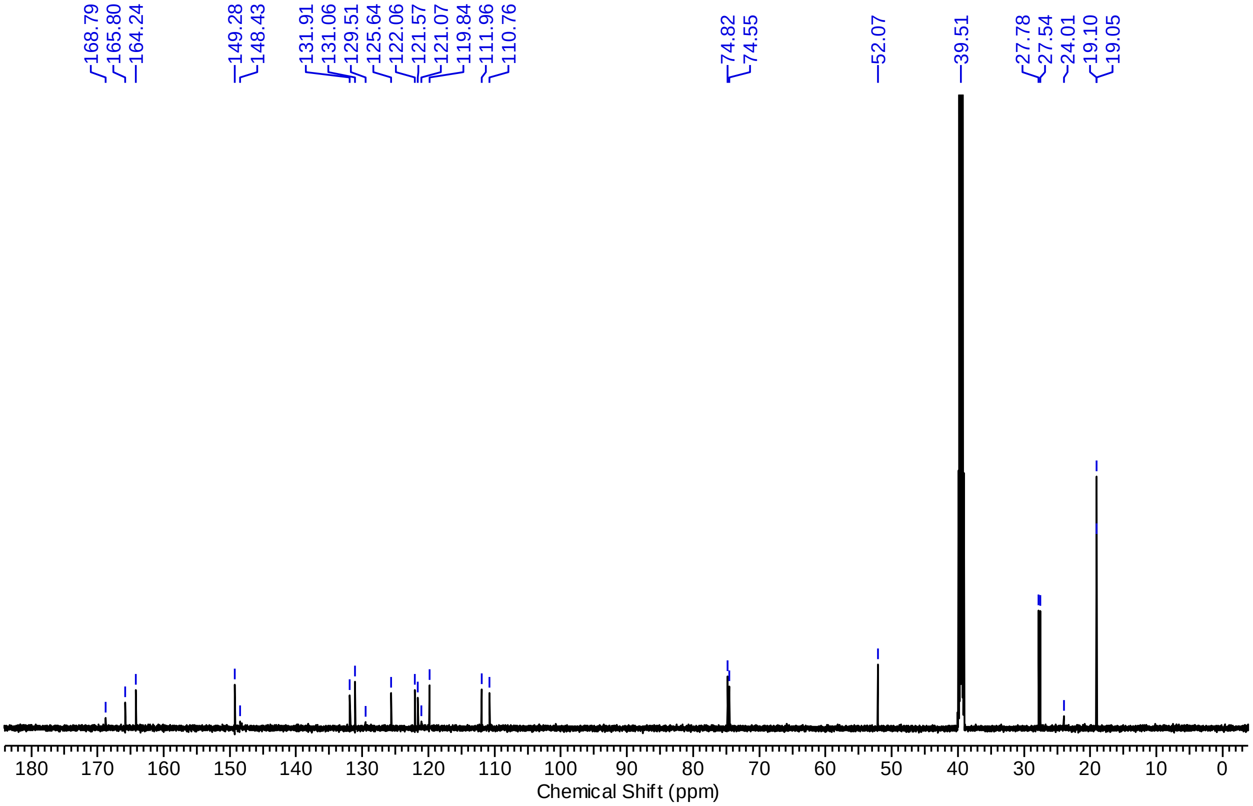

Compound **5b**

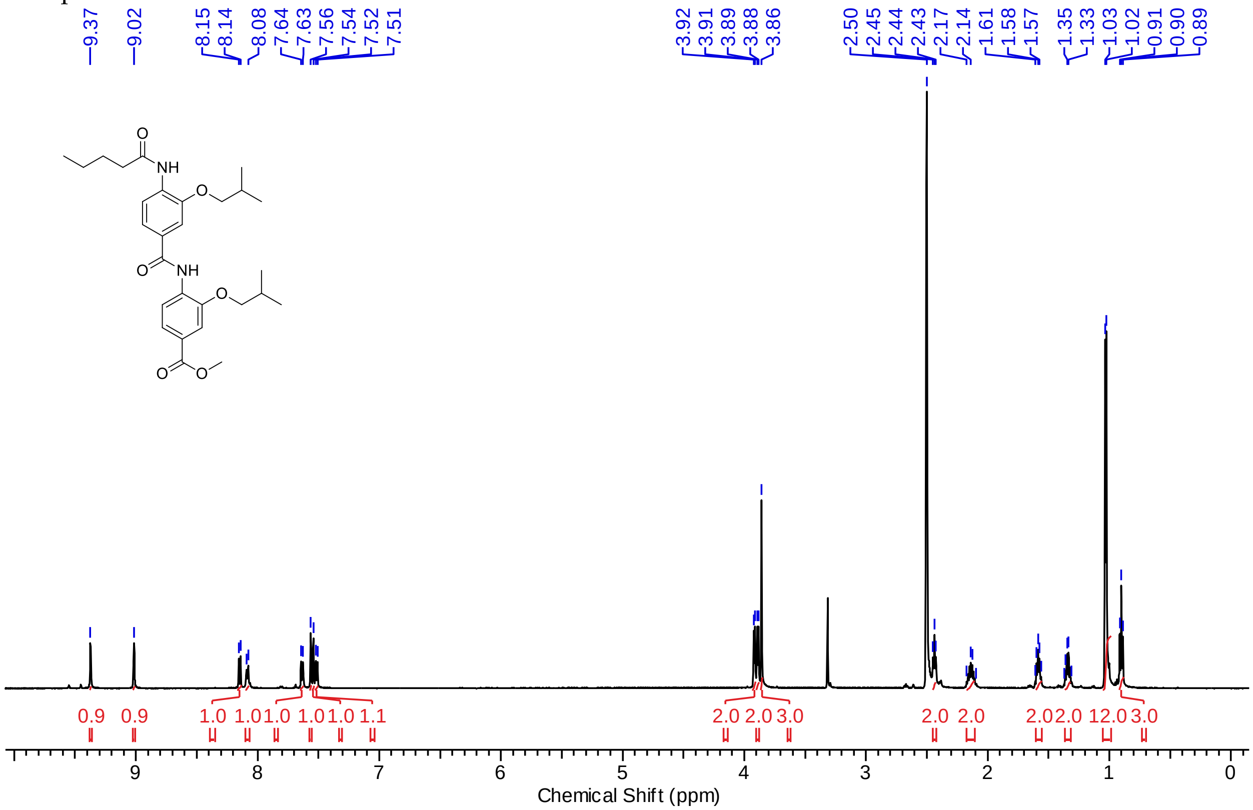
O
NH
O
O
NH
O
O
O
O
9.37
9.02
8.15
8.14
8.08
7.64
7.63
7.56
7.54
7.52
7.51
3.92
3.91
3.89
3.88
3.86
2.50
2.45
2.44
2.43
2.17
2.14
1.61
1.58
1.57
1.35
1.33
1.03
1.02
0.91
0.90
0.89
0.9 0.9 1.0 1.0 1.0 1.0 1.0 1.1
2.0 2.0 3.0
2.0 2.0
2.0 2.0 12.0 3.0
9 8 7 6 5 4 3 2 1 0
Chemical Shift (ppm)

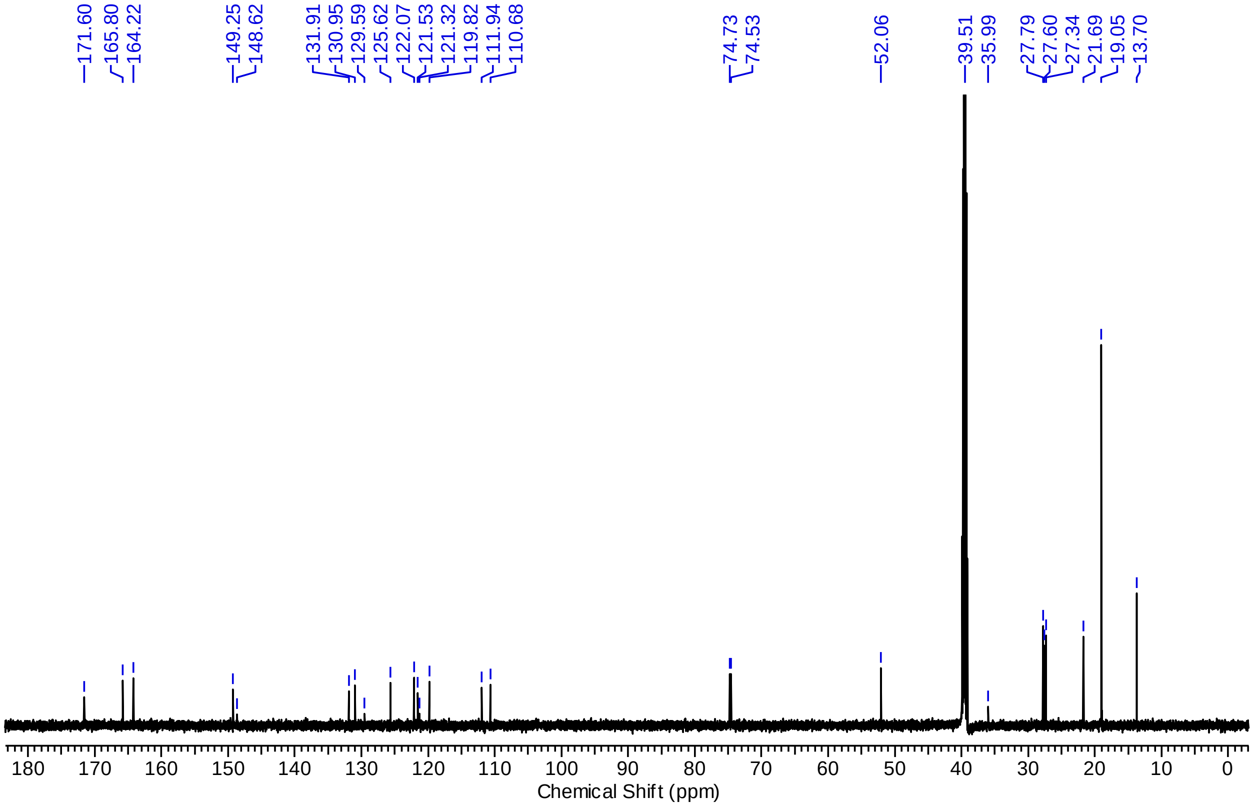
171.60
165.80
164.22
149.25
148.62
131.91
130.95
129.59
125.62
122.07
121.53
121.32
119.82
111.94
110.68
74.73
74.53
52.06
39.51
35.99
27.79
27.60
27.34
21.69
19.05
13.70
180 170 160 150 140 130 120 110 100 90 80 70 60 50 40 30 20 10 0
Chemical Shift (ppm)

Compound **5c**

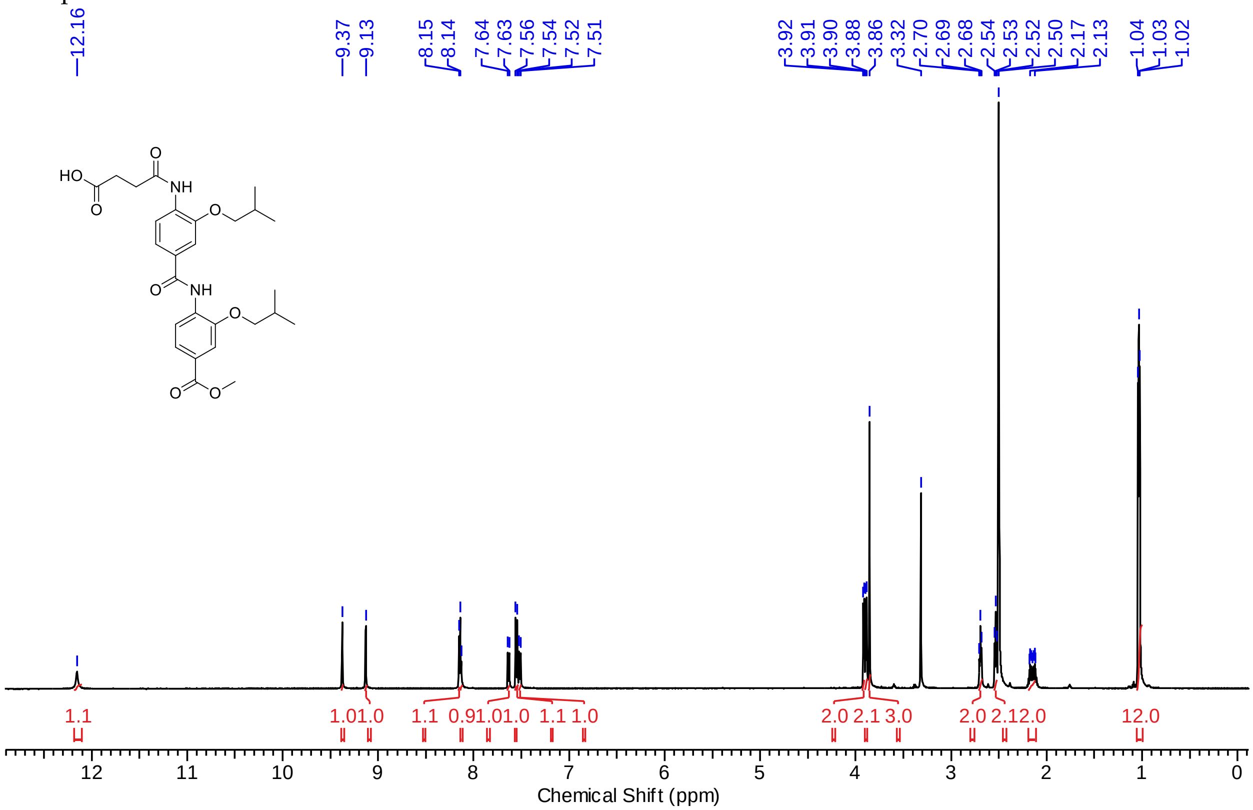
HO
O
O
NH
O
O
NH
O
O
O
12.16
9.37
9.13
8.15
8.14
7.64
7.63
7.56
7.54
7.52
7.51
3.92
3.91
3.90
3.88
3.86
3.32
2.70
2.69
2.68
2.54
2.53
2.52
2.50
2.17
2.13
1.04
1.03
1.02
1.1
1.0
1.0
1.1
0.9
1.0
1.0
1.1
1.0
2.0
2.1
3.0
2.0
2.1
2.0
12.0
Chemical Shift (ppm)

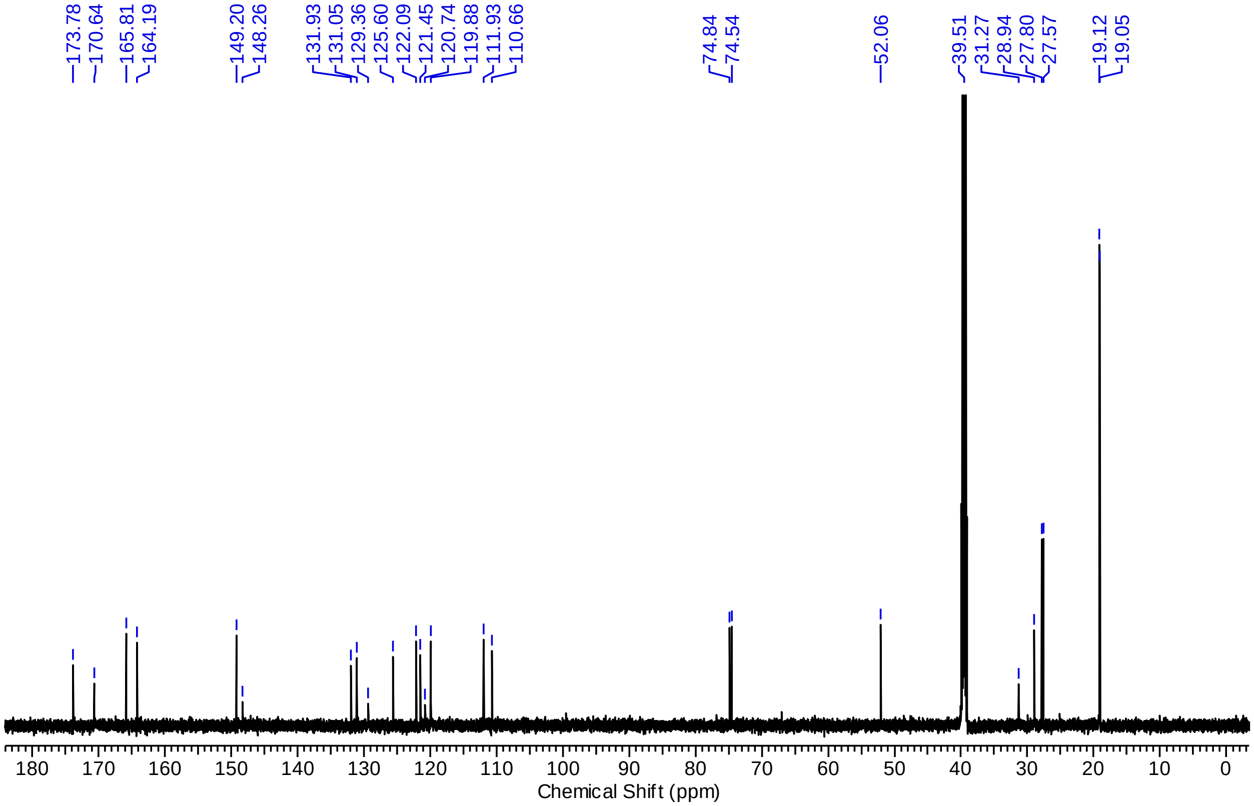
173.78
170.64
165.81
164.19
149.20
148.26
131.93
131.05
129.36
125.60
122.09
121.45
120.74
119.88
111.93
110.66
74.84
74.54
52.06
39.51
31.27
28.94
27.80
27.57
19.12
19.05
Chemical Shift (ppm)

Compound **5e**

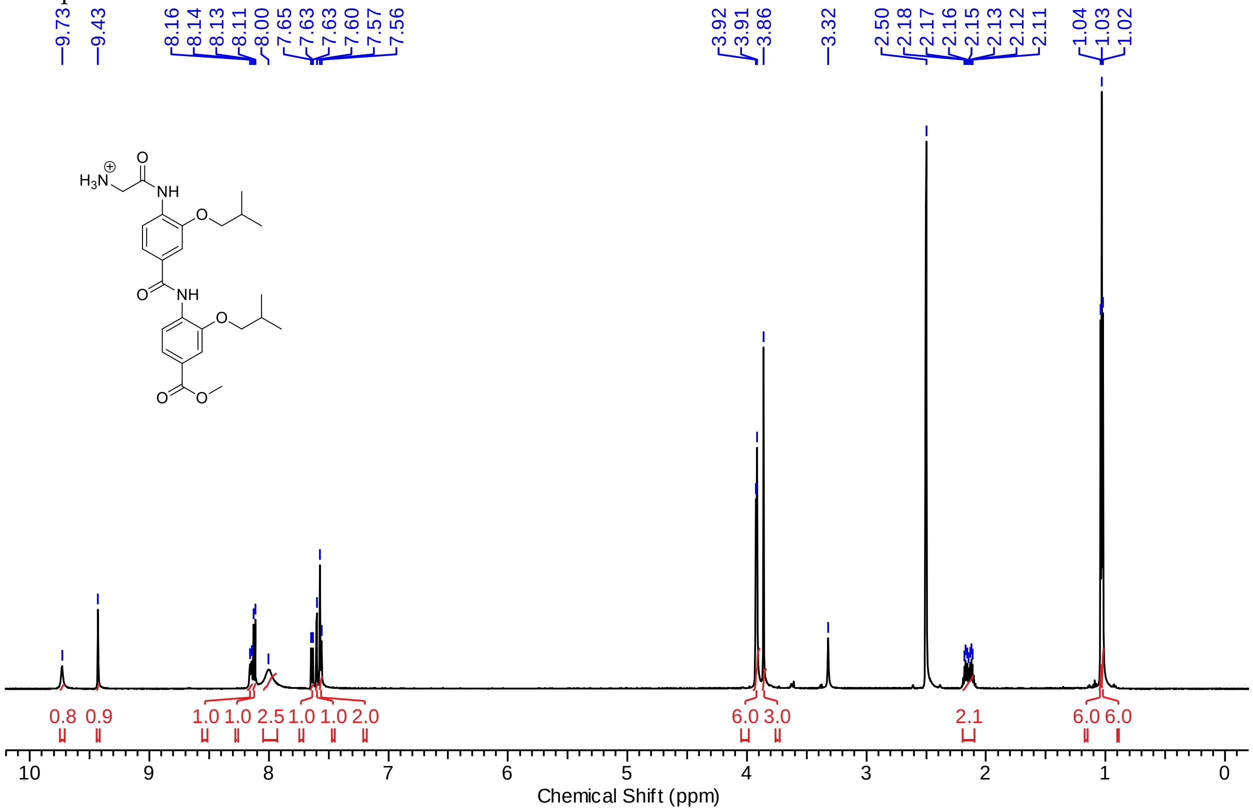
9.73
9.43
8.16
8.14
8.13
8.11
8.00
7.65
7.63
7.63
7.60
7.57
7.56
3.92
3.91
3.86
3.32
2.50
2.18
2.17
2.16
2.15
2.13
2.12
2.11
1.04
1.03
1.02
0.8 0.9 1.0 1.0 2.5 1.0 1.0 2.0 6.0 3.0 2.1 6.0 6.0
10 9 8 7 6 5 4 3 2 1 0
Chemical Shift (ppm)

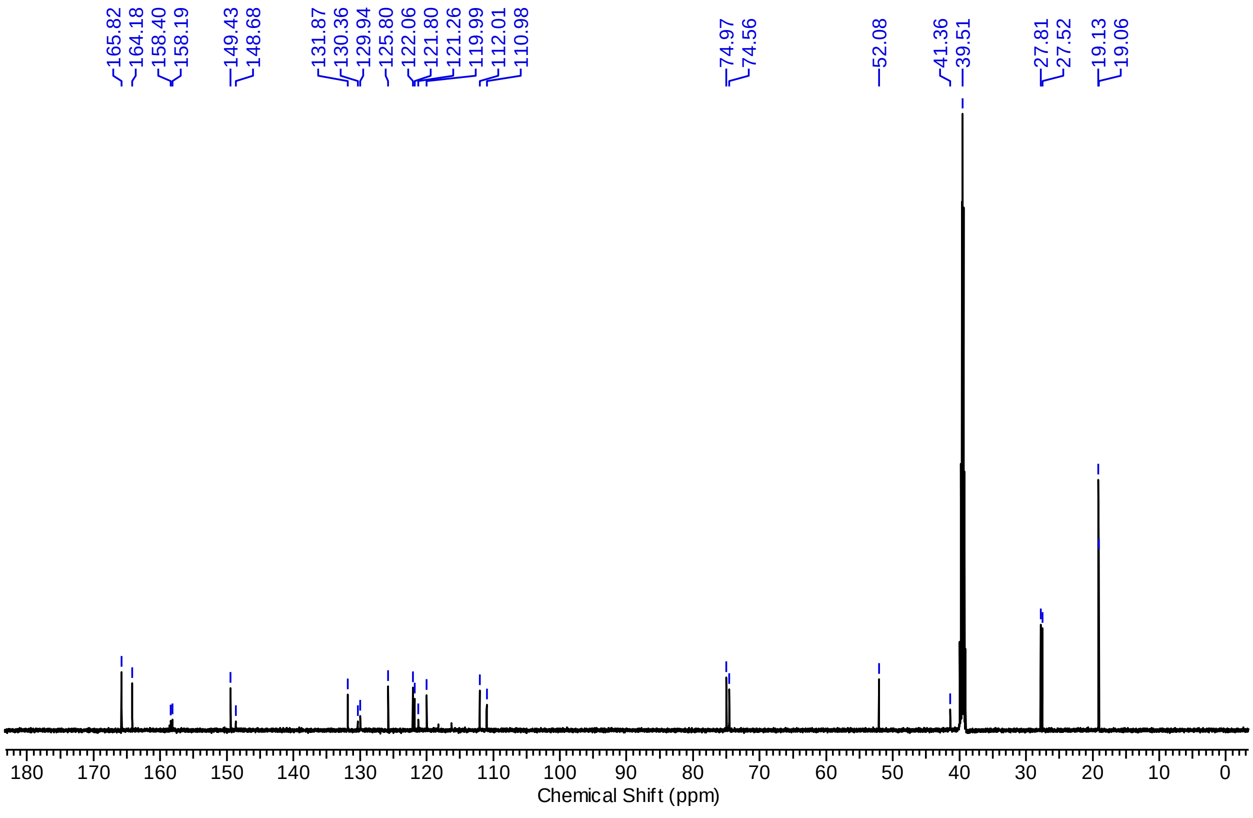
165.82
164.18
158.40
158.19
149.43
148.68
131.87
130.36
129.94
125.80
122.06
121.80
121.26
119.99
112.01
110.98
74.97
74.56
52.08
41.36
39.51
27.81
27.52
19.13
19.06
180 170 160 150 140 130 120 110 100 90 80 70 60 50 40 30 20 10 0
Chemical Shift (ppm)

Compound **7b**

TK_7b_D2-38_971.esp

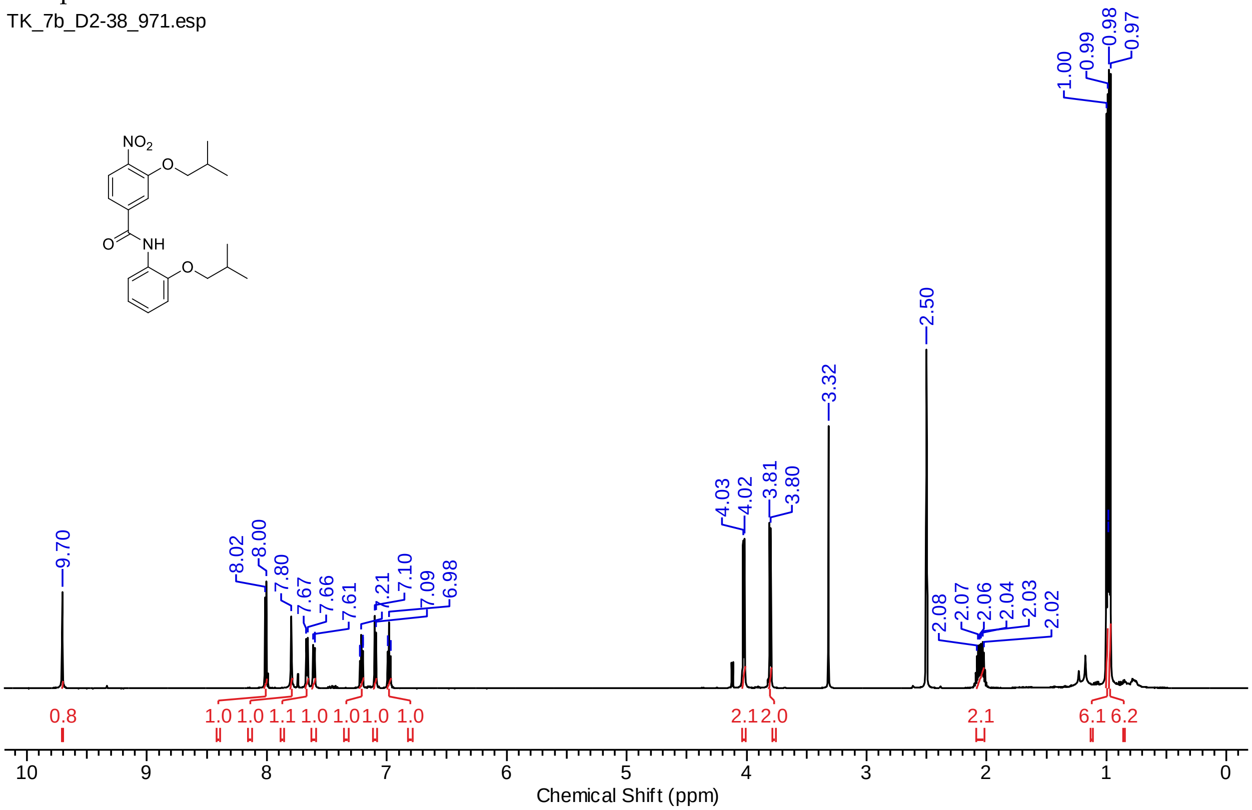

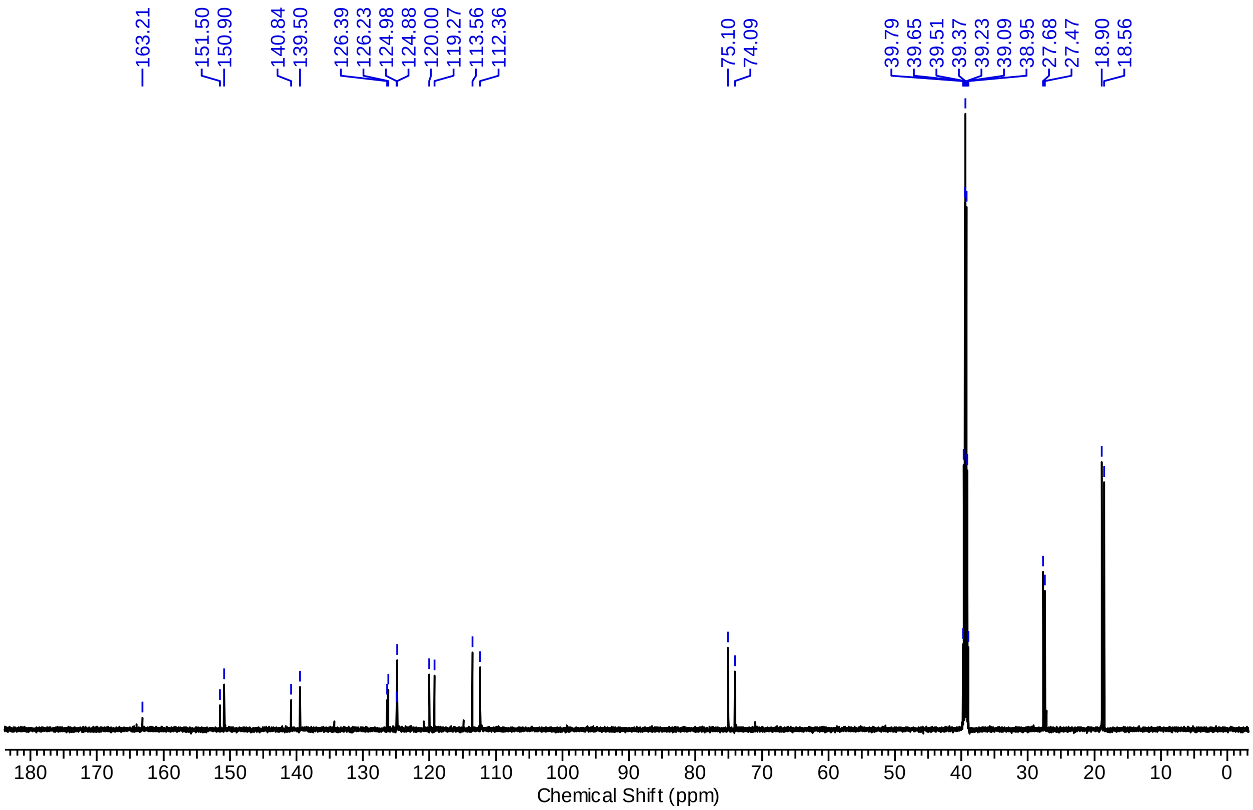

Compound **8**

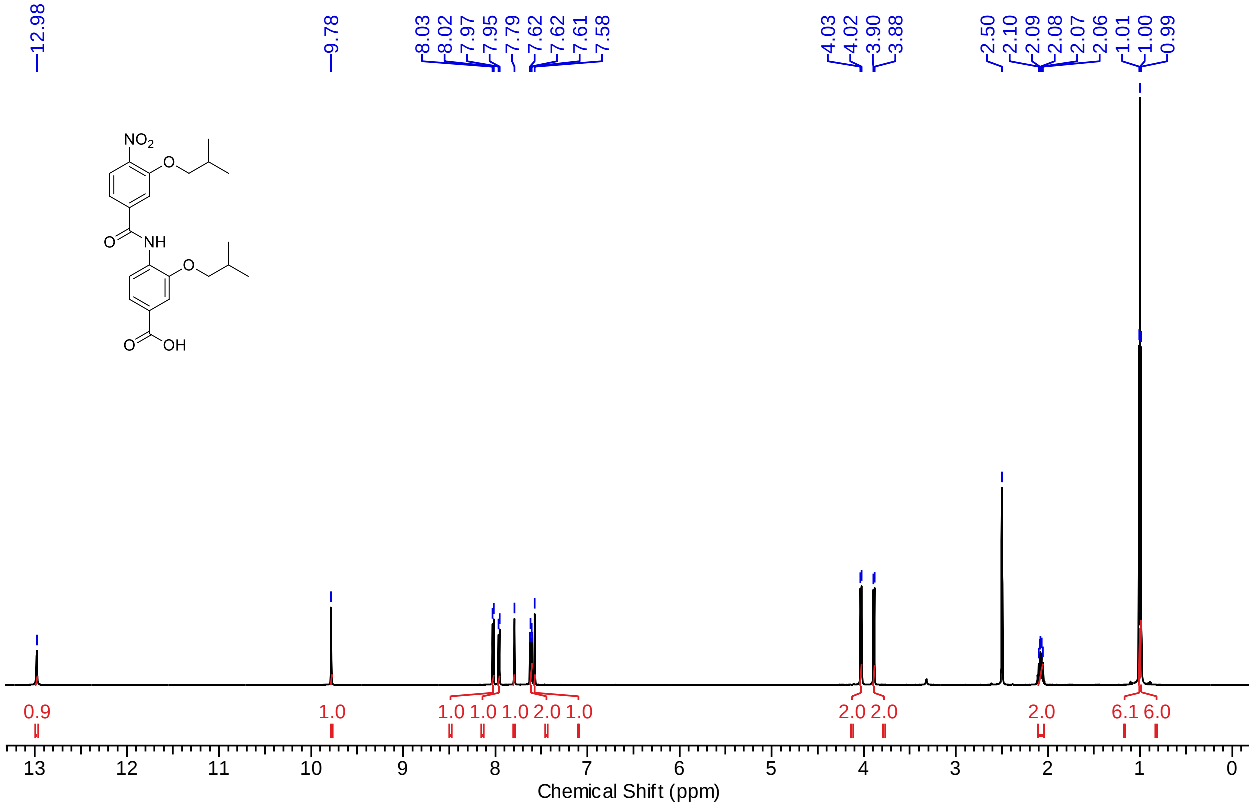
NO2
O
O
NH
O
O
OH
12.98
9.78
8.03
8.02
7.97
7.95
7.79
7.62
7.62
7.61
7.58
4.03
4.02
3.90
3.88
2.50
2.10
2.09
2.08
2.07
2.06
1.01
1.00
0.99
0.9
1.0
1.0
1.0
1.0
2.0
1.0
2.0
2.0
2.0
6.1
6.0
13
12
11
10
9
8
7
6
5
4
3
2
1
0
Chemical Shift (ppm)

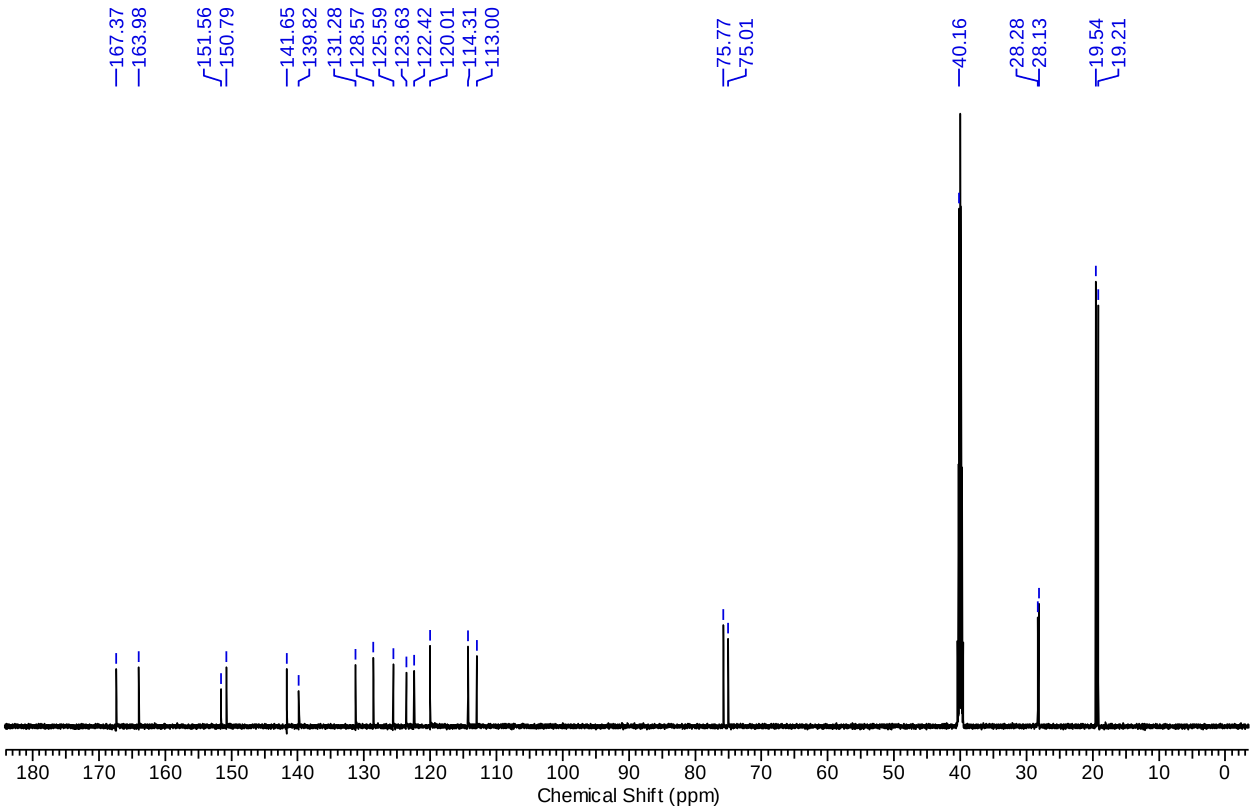
167.37
163.98
151.56
150.79
141.65
139.82
131.28
128.57
125.59
123.63
122.42
120.01
114.31
113.00
75.77
75.01
40.16
28.28
28.13
19.54
19.21
180
170
160
150
140
130
120
110
100
90
80
70
60
50
40
30
20
10
0
Chemical Shift (ppm)

Compound **9a**

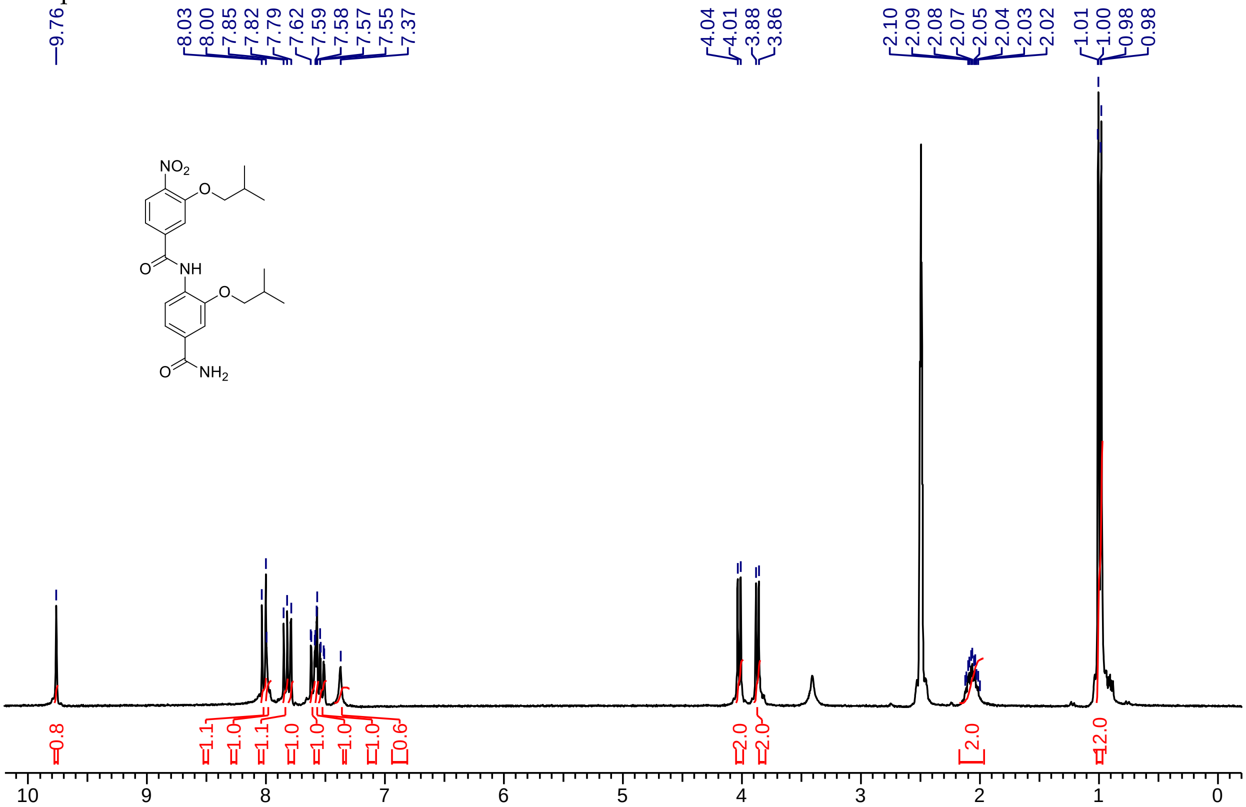

9.76
8.03 8.00 7.85 7.82 7.79 7.62 7.59 7.58 7.57 7.55 7.37
4.04 4.01 3.88 3.86
2.10 2.09 2.08 2.07 2.05 2.04 2.03 2.02
1.01 1.00 0.98 0.98
NO2
O
NH
O
O
NH2
0.8 1.1 1.0 1.1 1.0 1.0 1.0 1.0 0.6 2.0 2.0 2.0 12.0
10 9 8 7 6 5 4 3 2 1 0

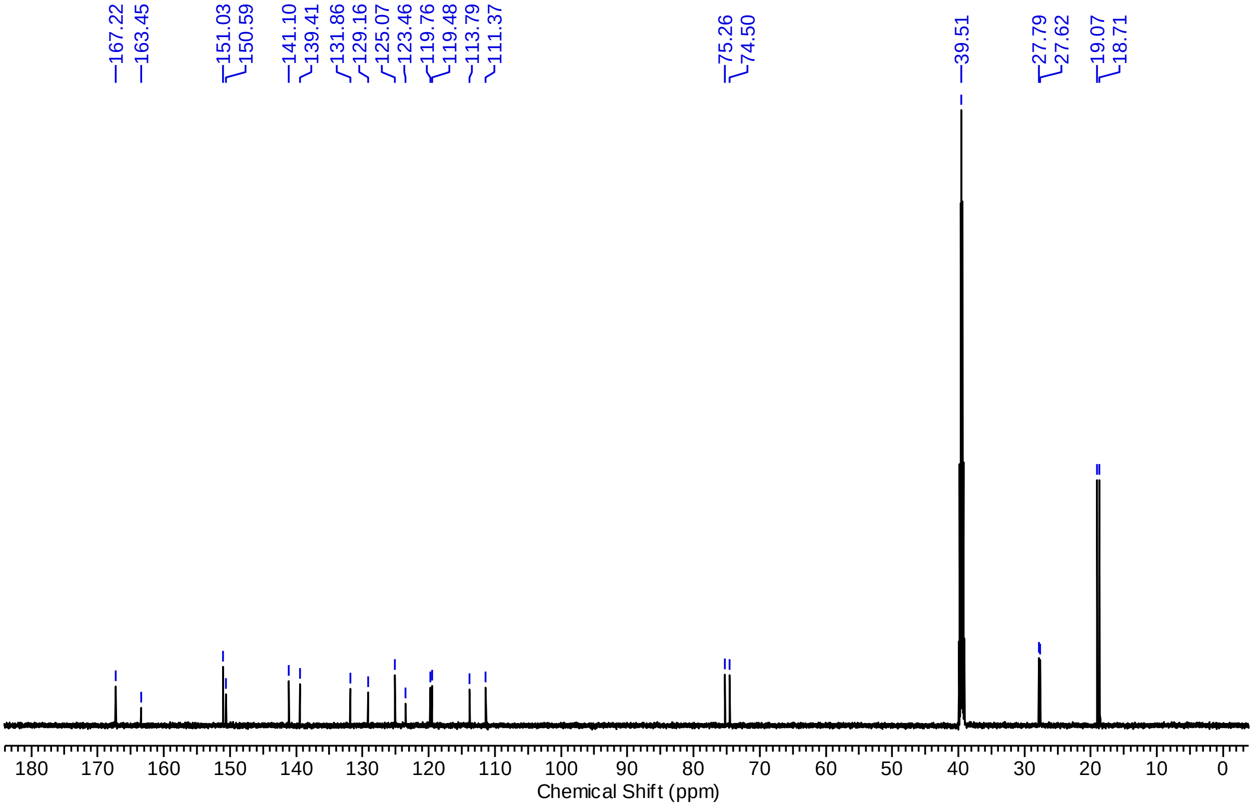

167.22 163.45
151.03 150.59
141.10 139.41 131.86 129.16 125.07 123.46 119.76 119.48 113.79 111.37
75.26 74.50
39.51
27.79 27.62
19.07 18.71
180 170 160 150 140 130 120 110 100 90 80 70 60 50 40 30 20 10 0
Chemical Shift (ppm)

Compound **9b**

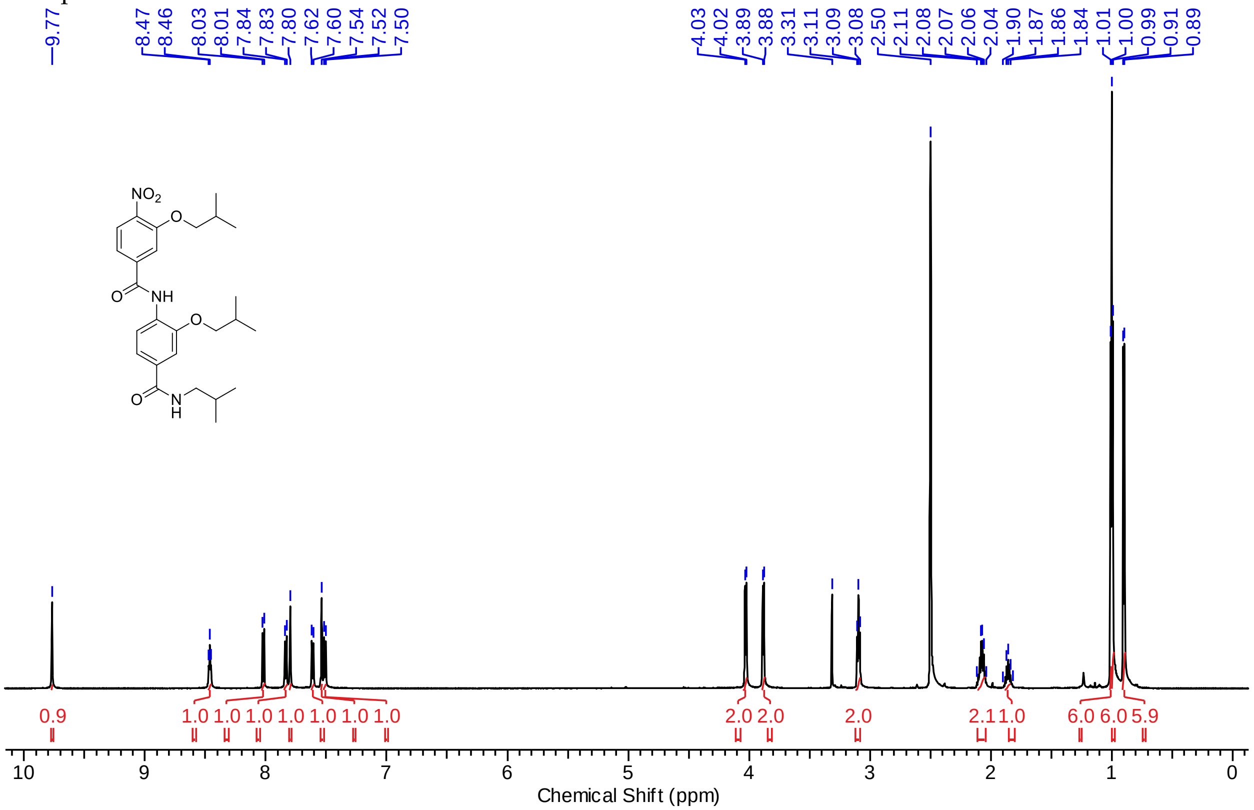
9.77
8.47 8.46 8.03 8.01 7.84 7.83 7.80 7.62 7.60 7.54 7.52 7.50
4.03 4.02 3.89 3.88 3.31 3.11 3.09 3.08 2.50 2.11 2.08 2.07 2.06 2.04 1.90 1.87 1.86 1.84 1.01 1.00 0.99 0.91 0.89
NO2
O
O
NH
O
O
N
H
0.9
1.0 1.0 1.0 1.0 1.0 1.0 1.0
2.0 2.0
2.0
2.1 1.0
6.0 6.0 5.9
Chemical Shift (ppm)

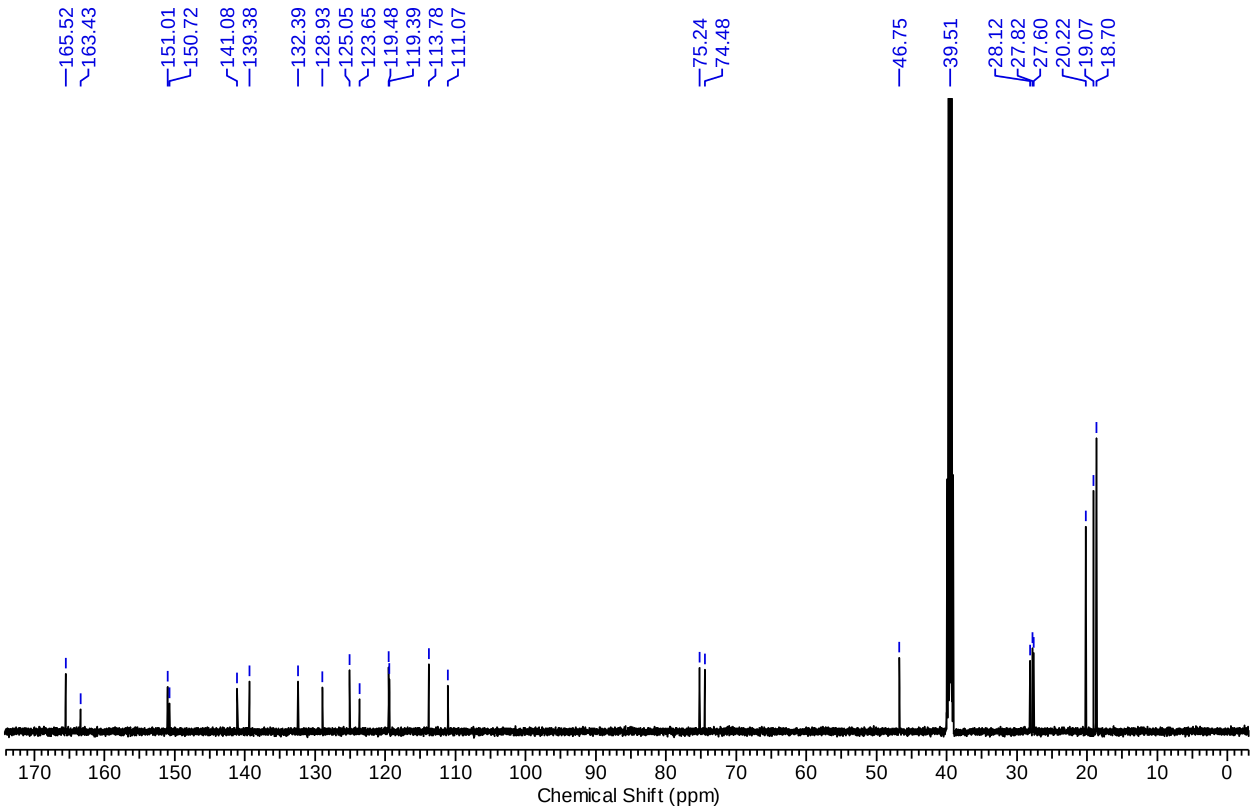
165.52 163.43
151.01 150.72
141.08 139.38
132.39 128.93 125.05 123.65 119.48 119.39 113.78 111.07
75.24 74.48
46.75
39.51
28.12 27.82 27.60 20.22 19.07 18.70
Chemical Shift (ppm)

Compound **9d**

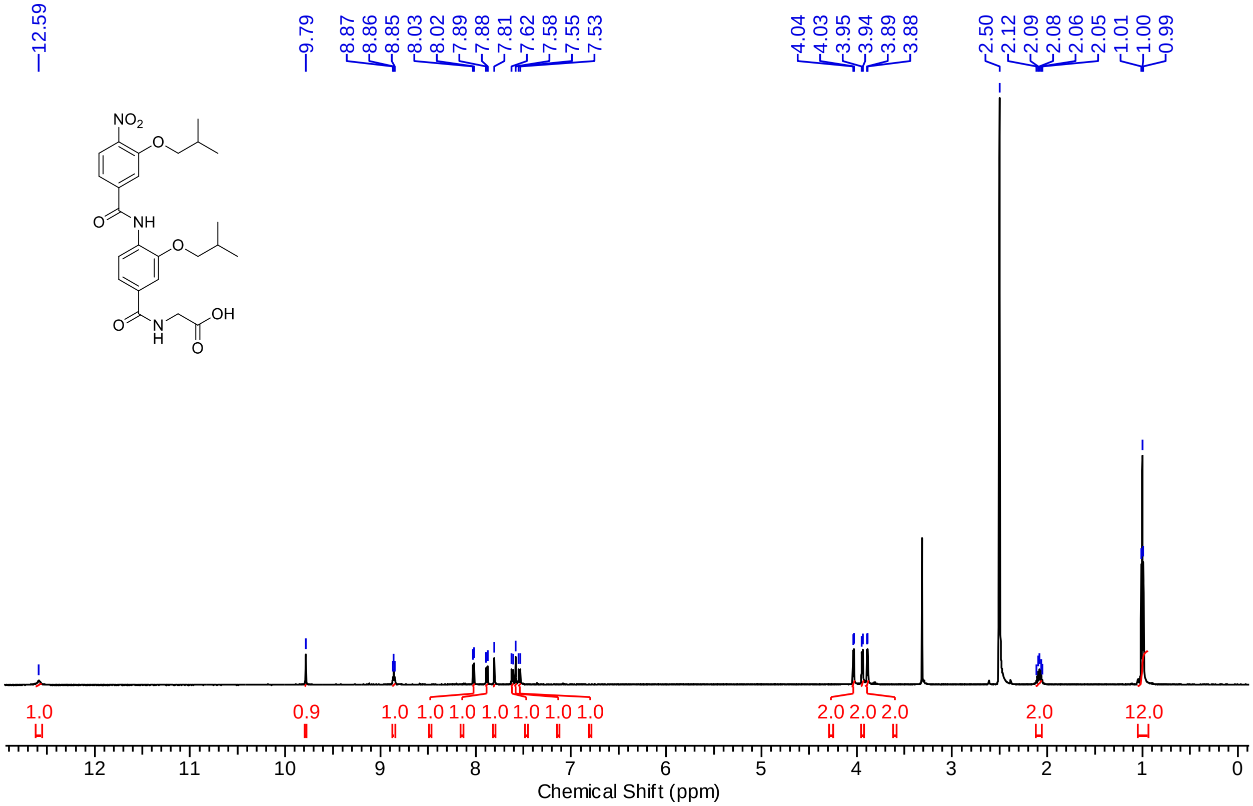

12.59
9.79
8.87
8.86
8.85
8.03
8.02
7.89
7.88
7.81
7.62
7.58
7.55
7.53
4.04
4.03
3.95
3.94
3.89
3.88
2.50
2.12
2.09
2.08
2.06
2.05
1.01
1.00
0.99
NO2
O
O
NH
O
N
H
OH
O
1.0
0.9
1.0 1.0 1.0 1.0 1.0 1.0 1.0
2.0 2.0 2.0
2.0
12.0
12
11
10
9
8
7
6
5
4
3
2
1
0
Chemical Shift (ppm)

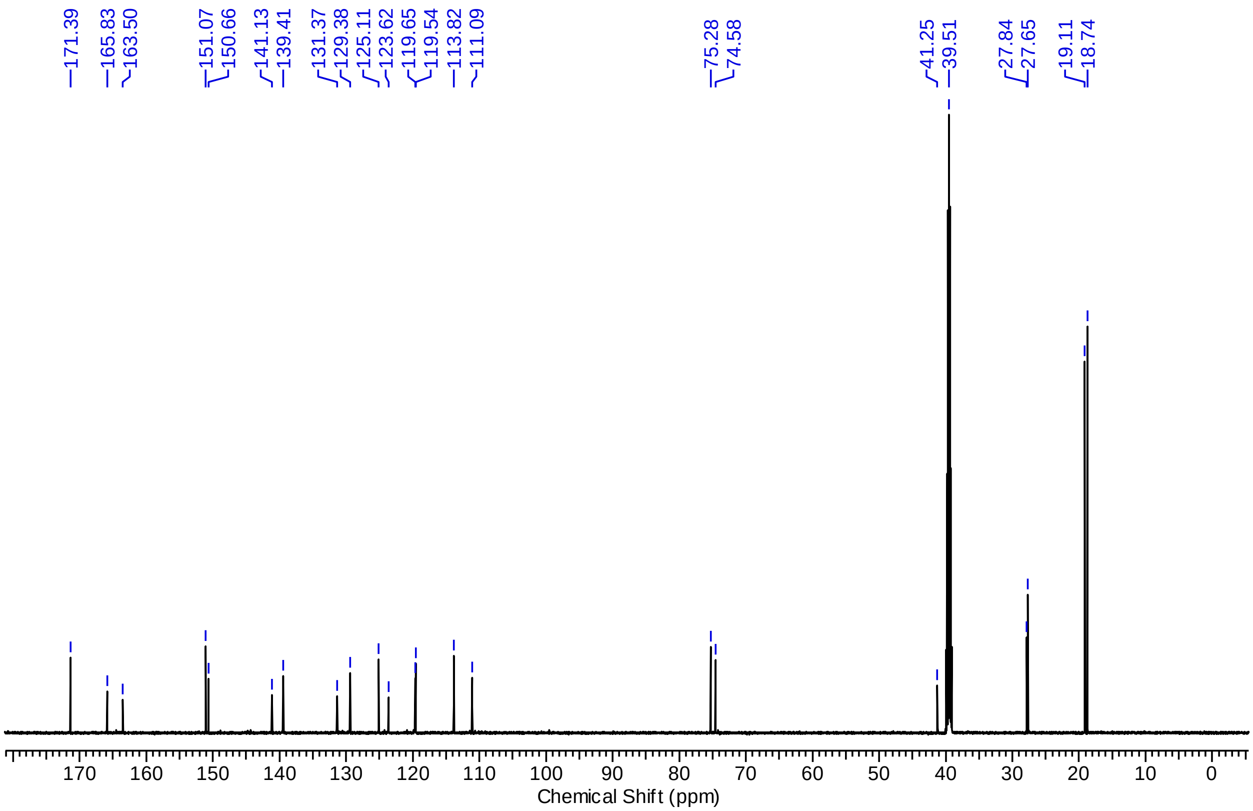

171.39
165.83
163.50
151.07
150.66
141.13
139.41
131.37
129.38
125.11
123.62
119.65
119.54
113.82
111.09
75.28
74.58
41.25
39.51
27.84
27.65
19.11
18.74
170
160
150
140
130
120
110
100
90
80
70
60
50
40
30
20
10
0
Chemical Shift (ppm)

Compound **14a**

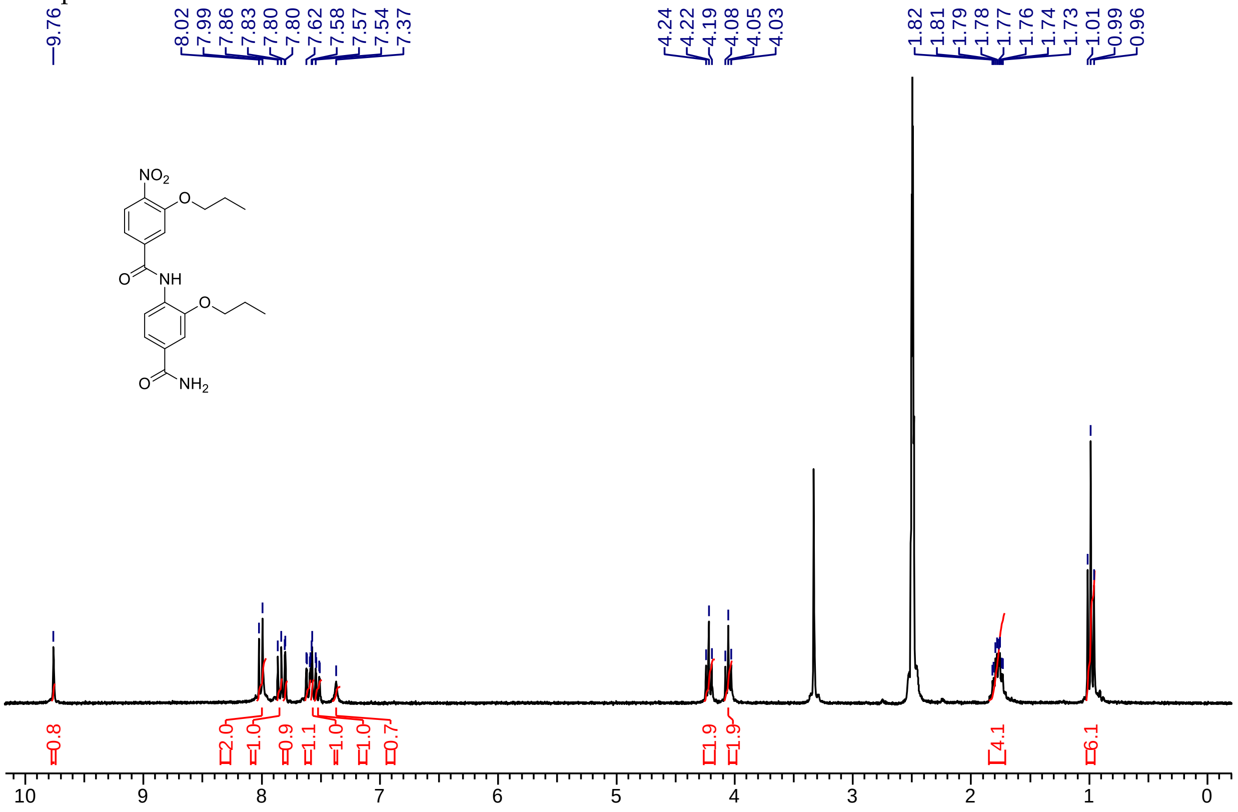

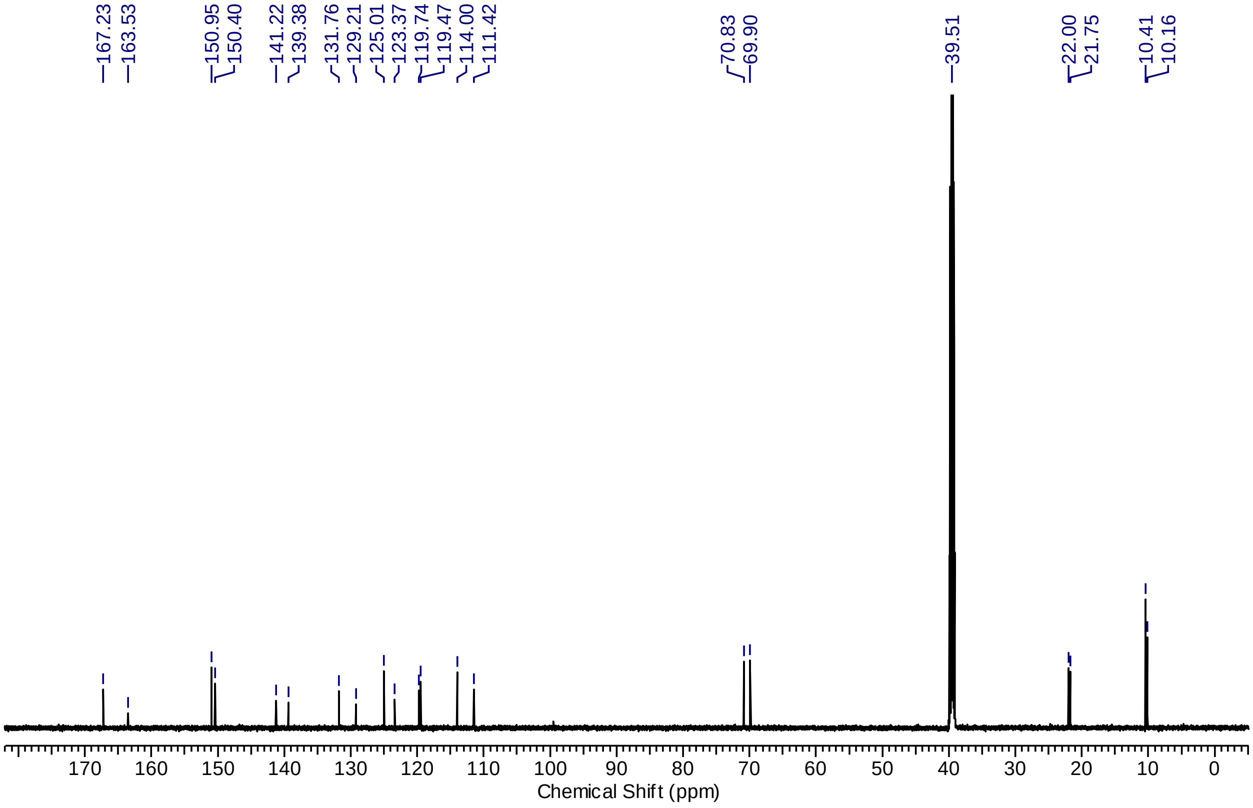

Compound **14b**

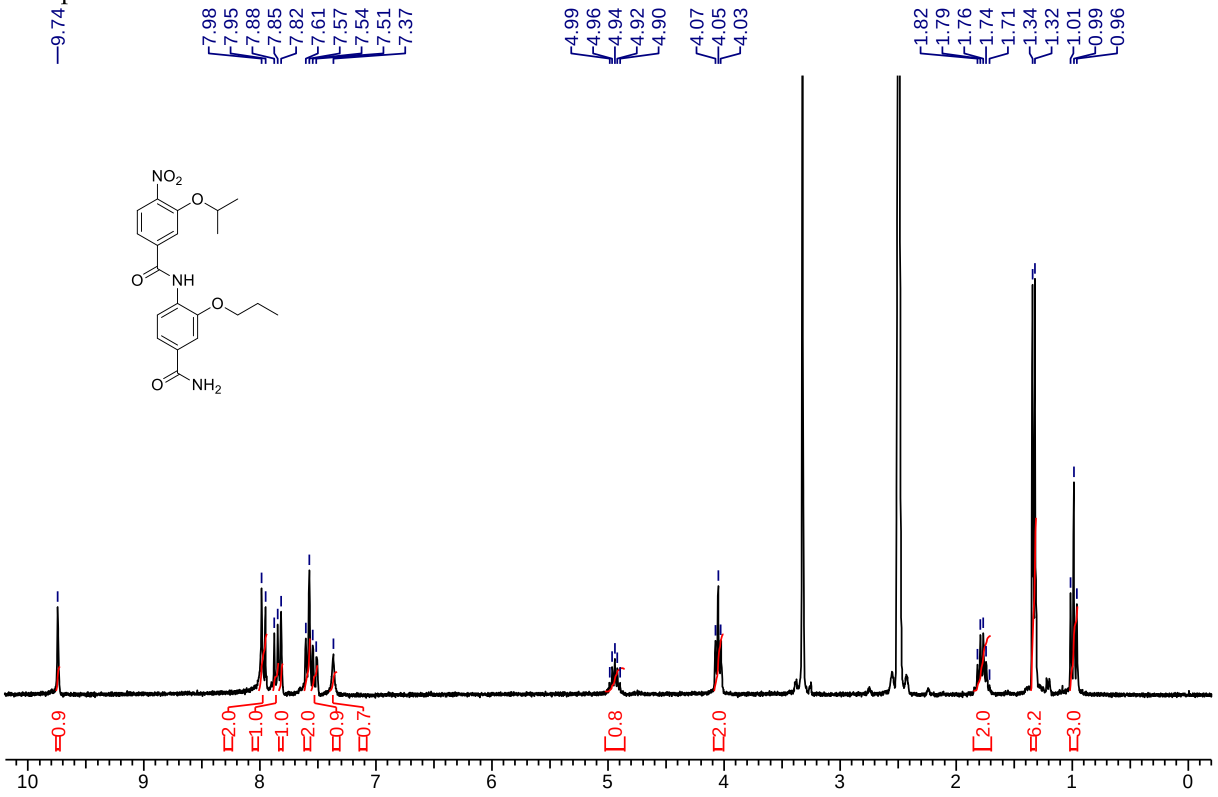

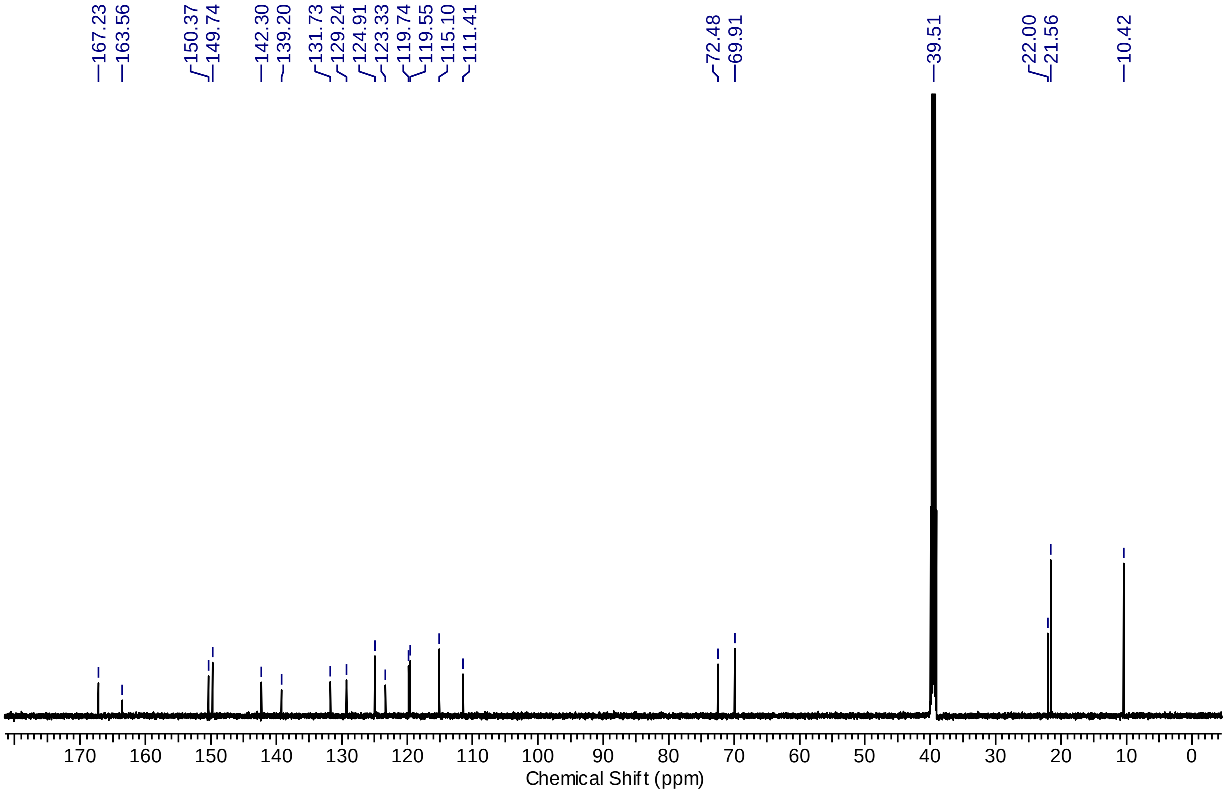

Compound **14c**

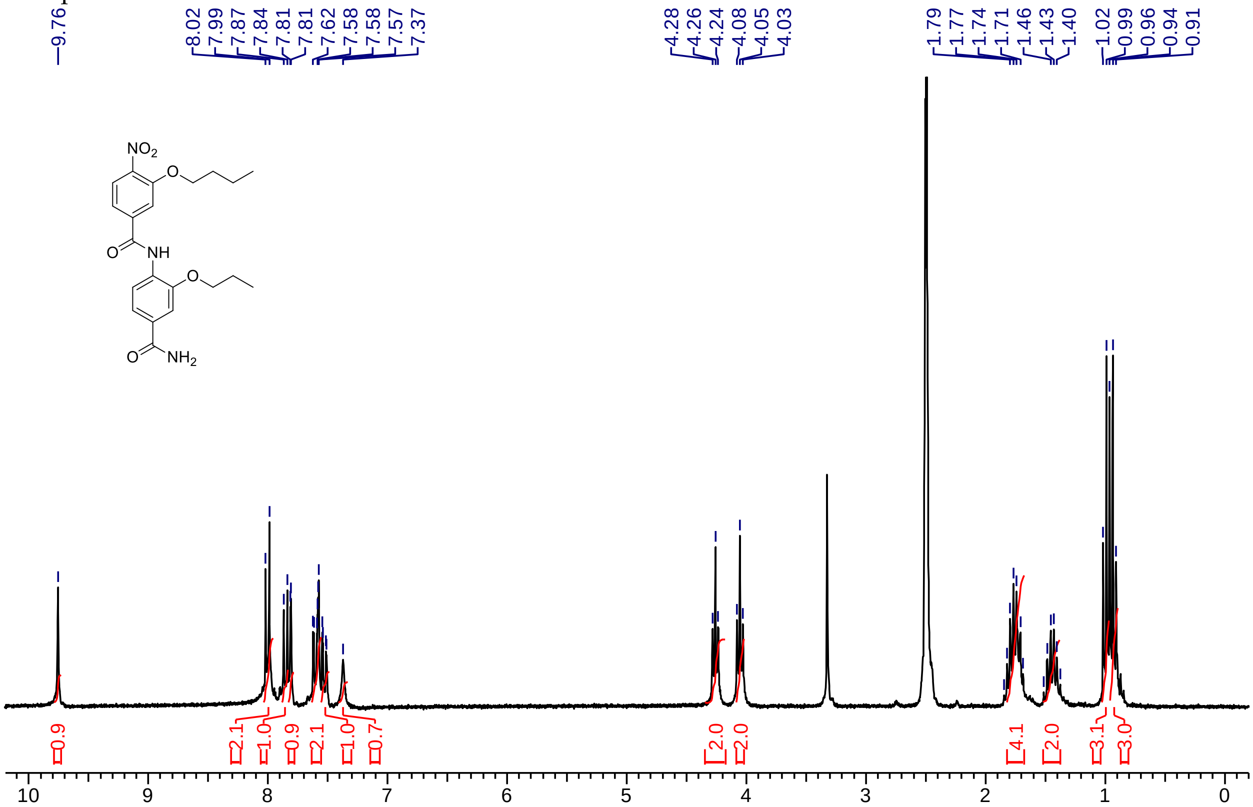

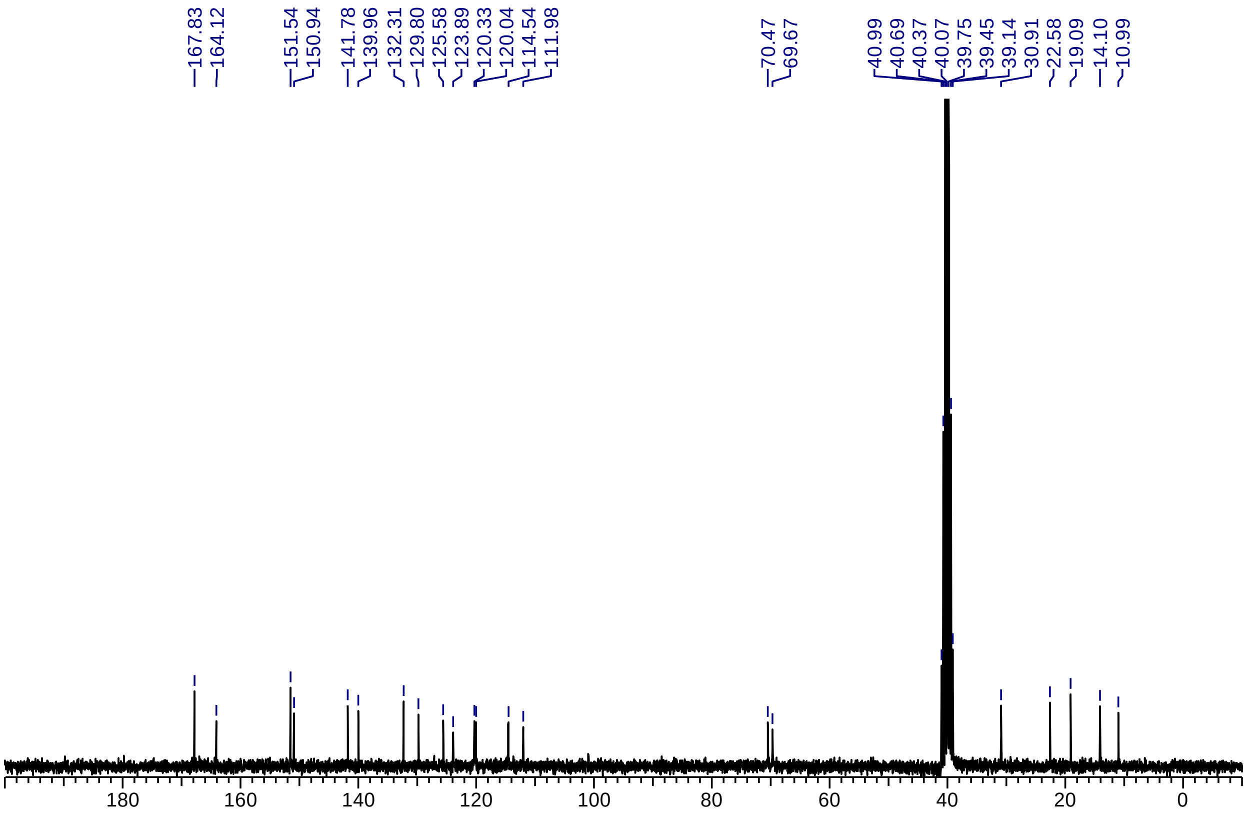

Compound **14d**

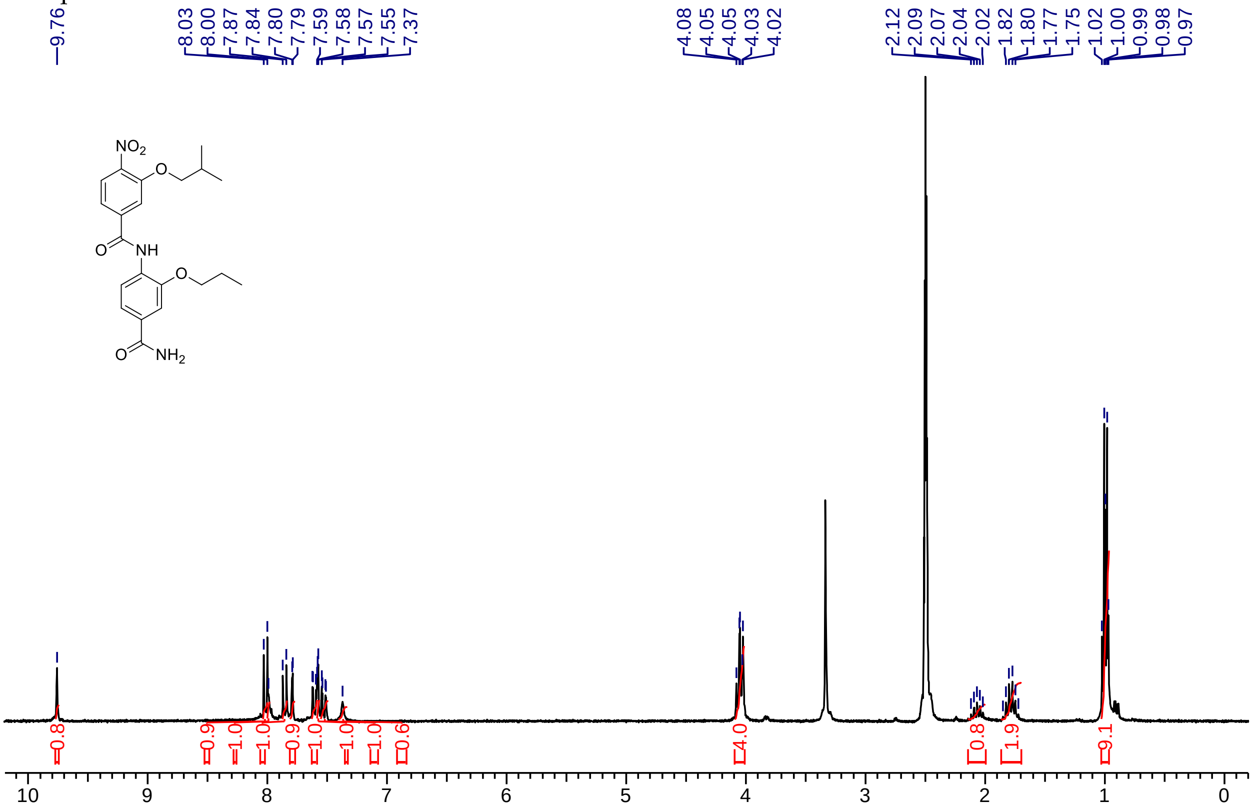

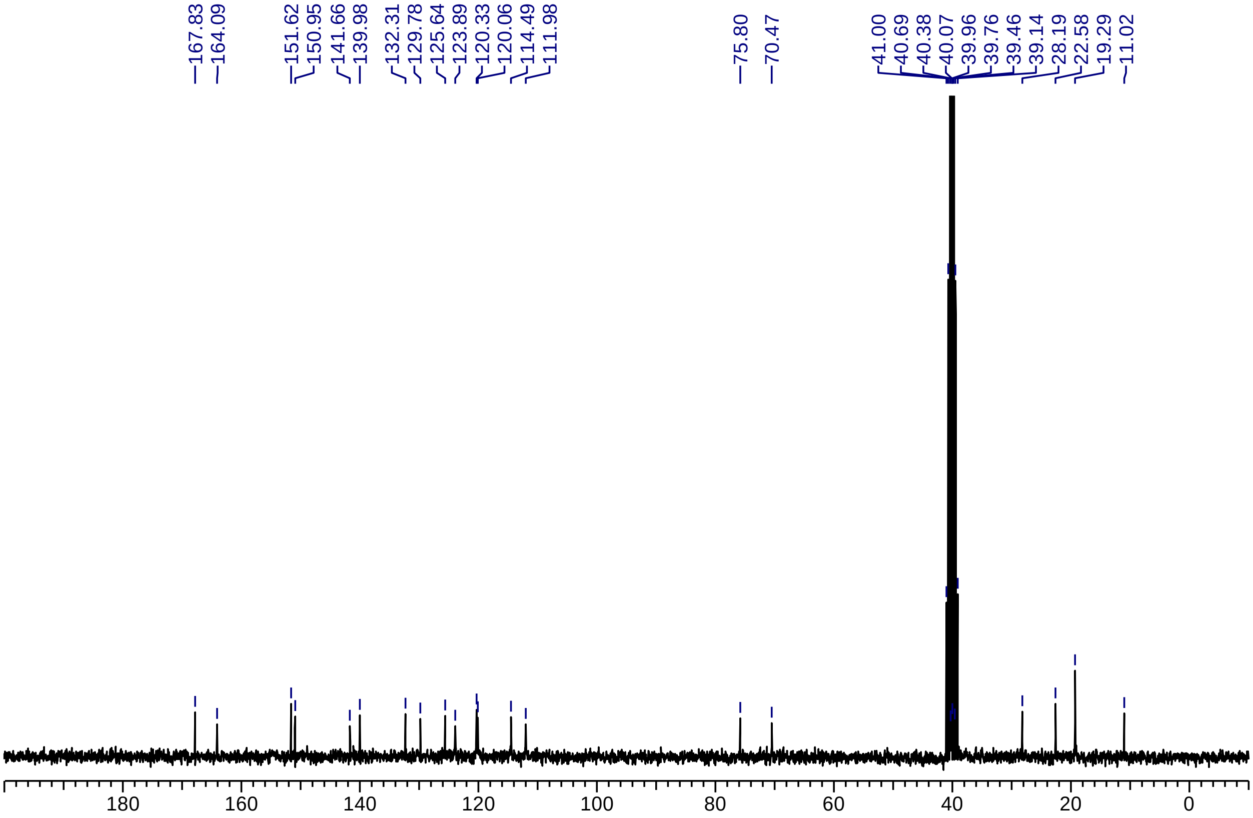

Compound **14e**

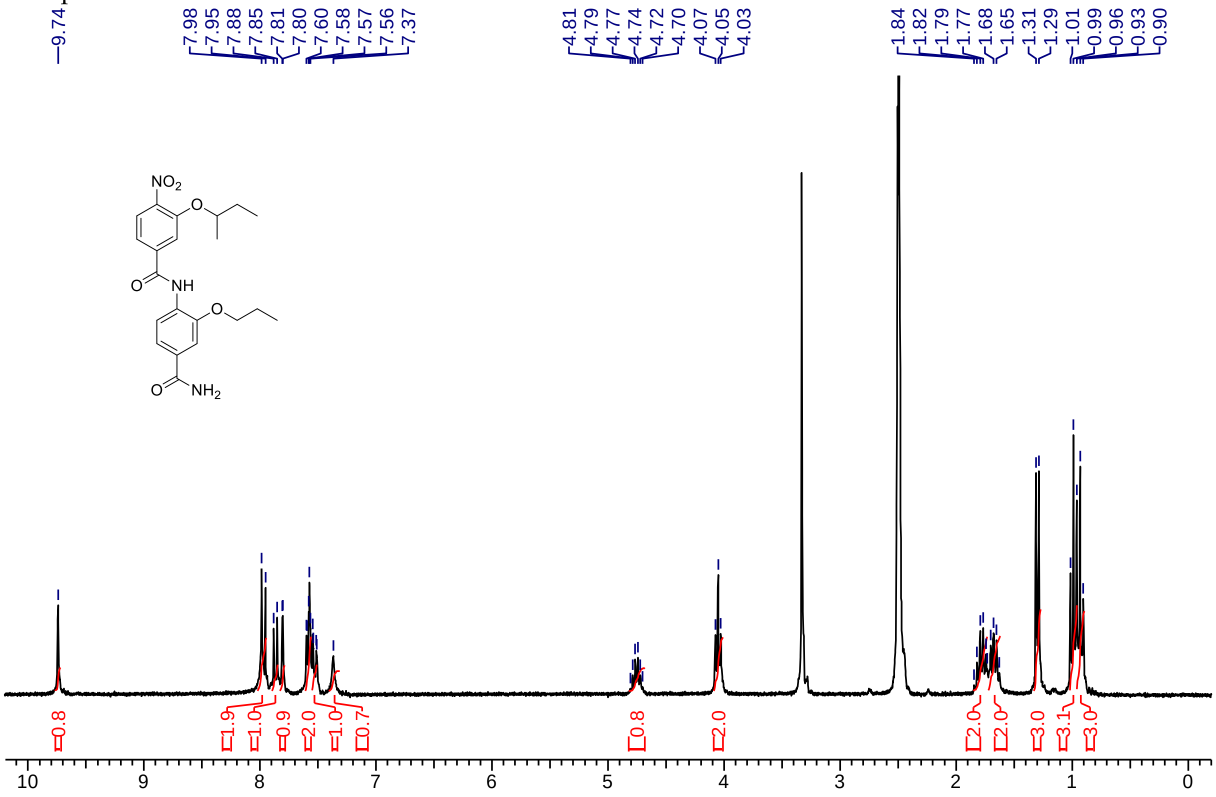

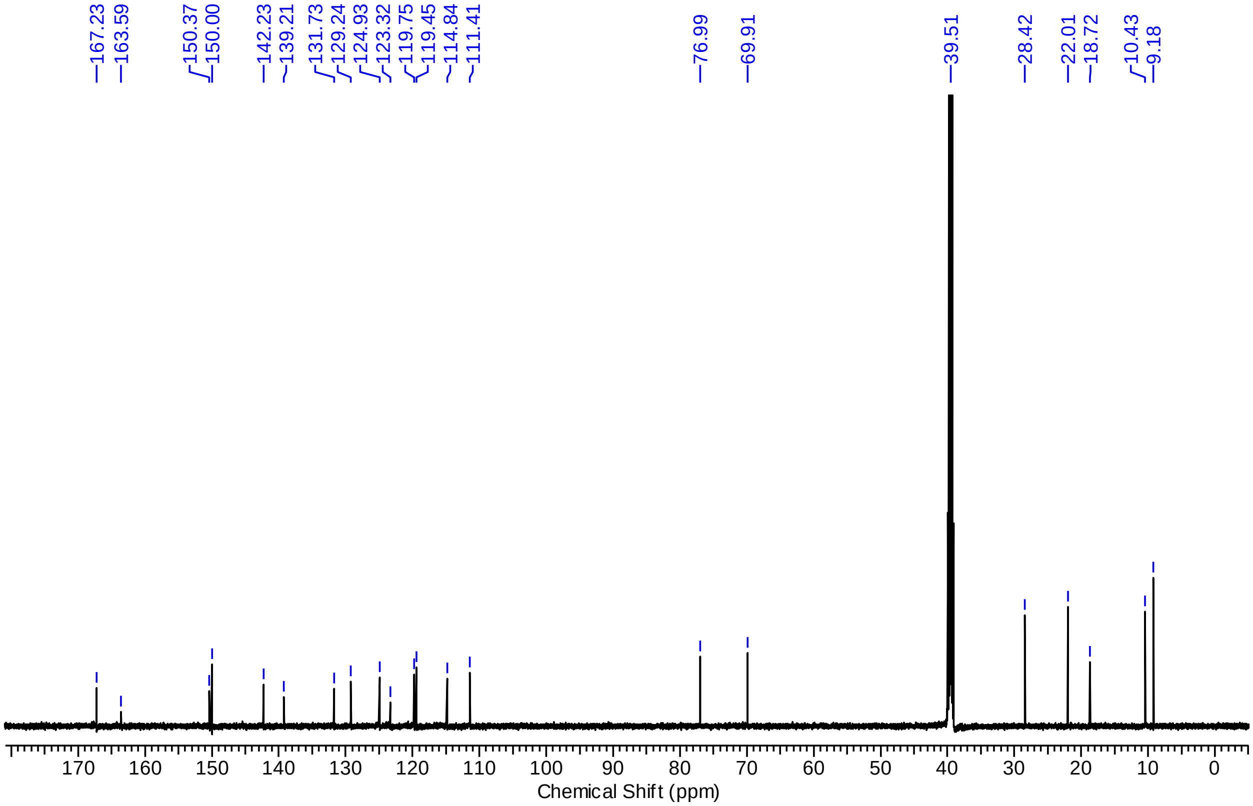

Compound **14f**

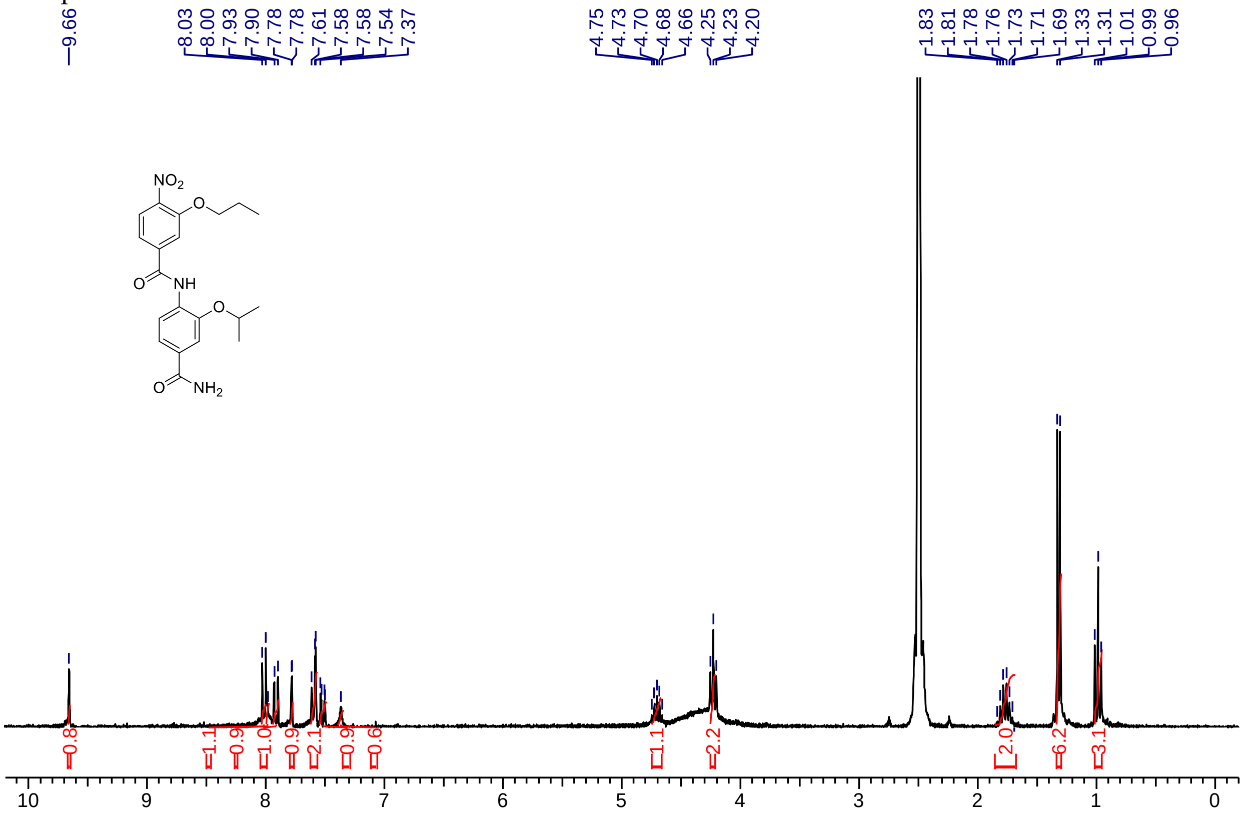

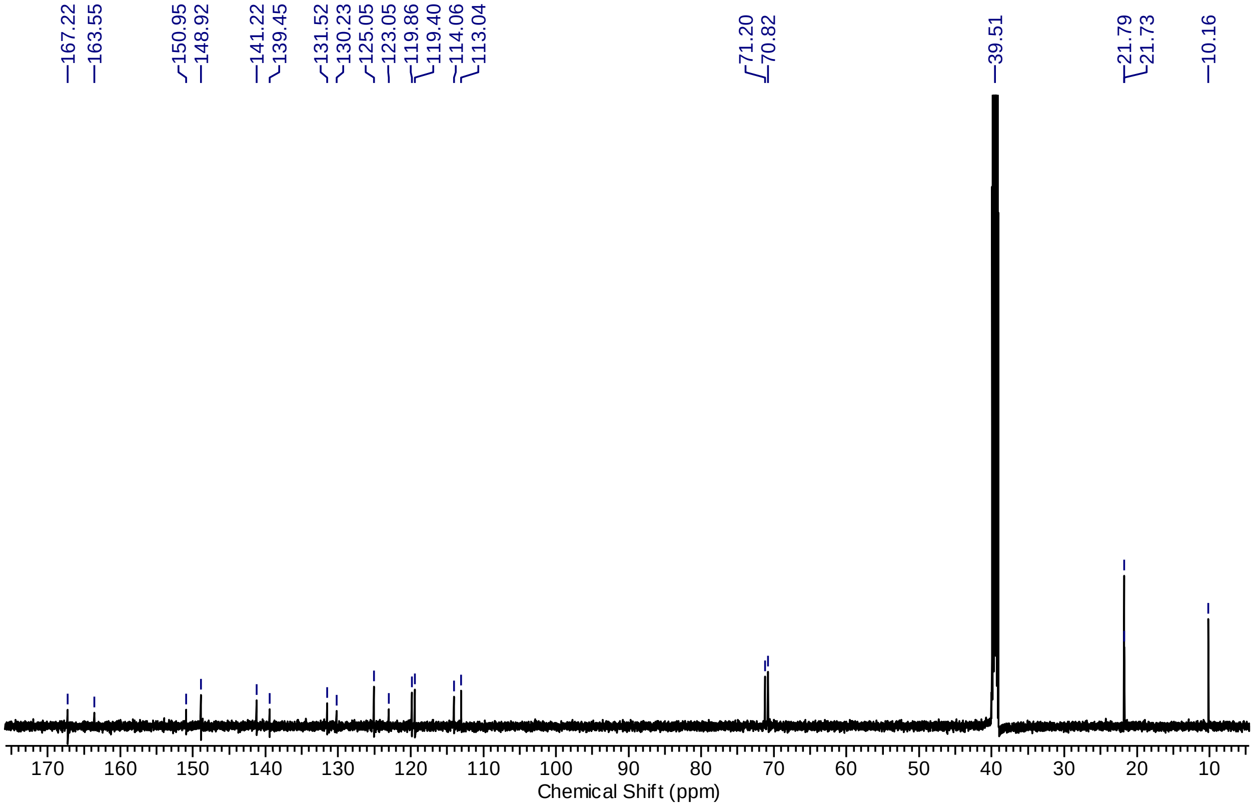

Compound **14g**

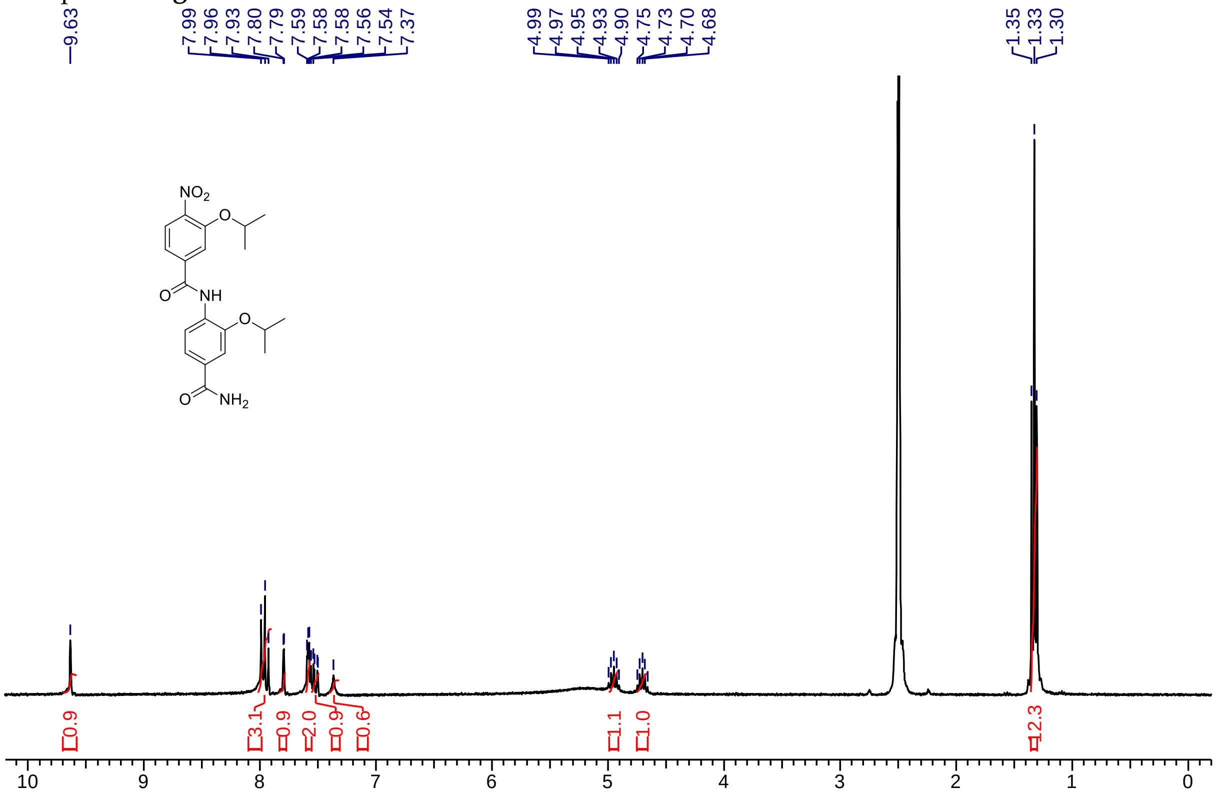

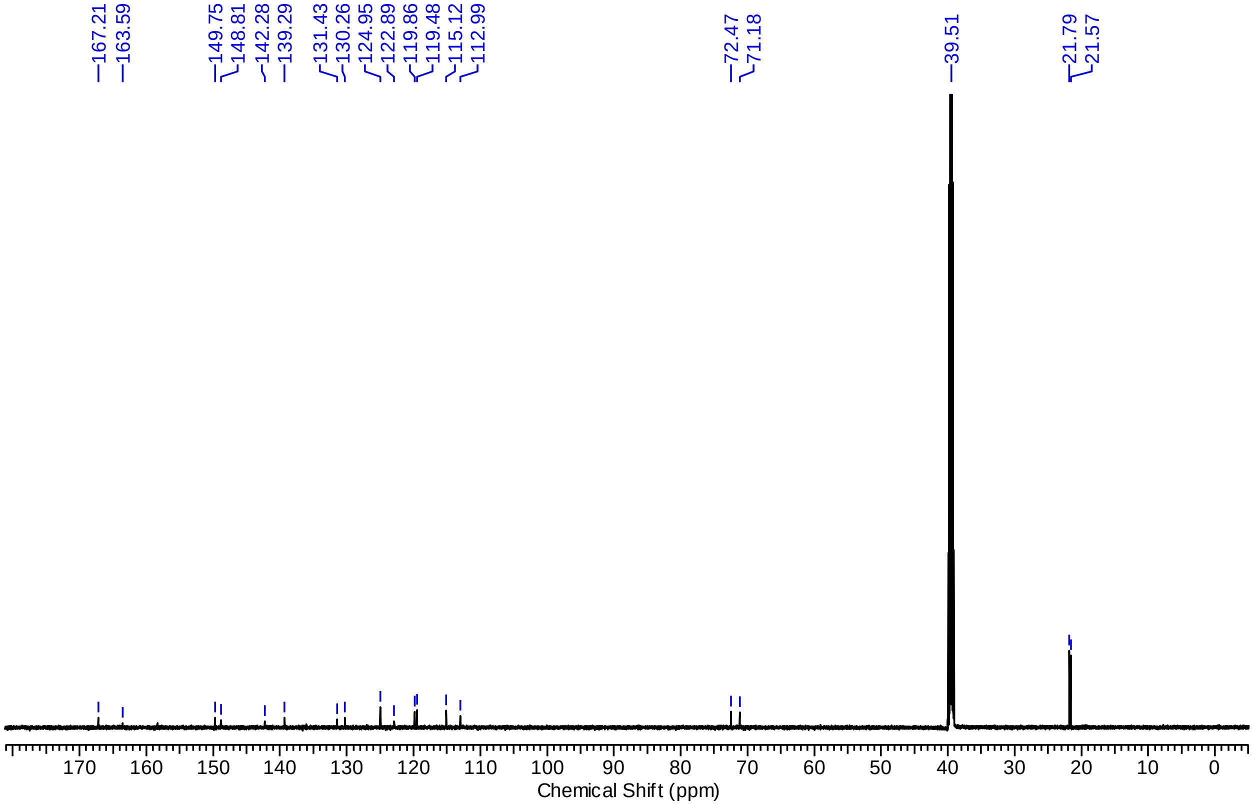

Compound **14h**

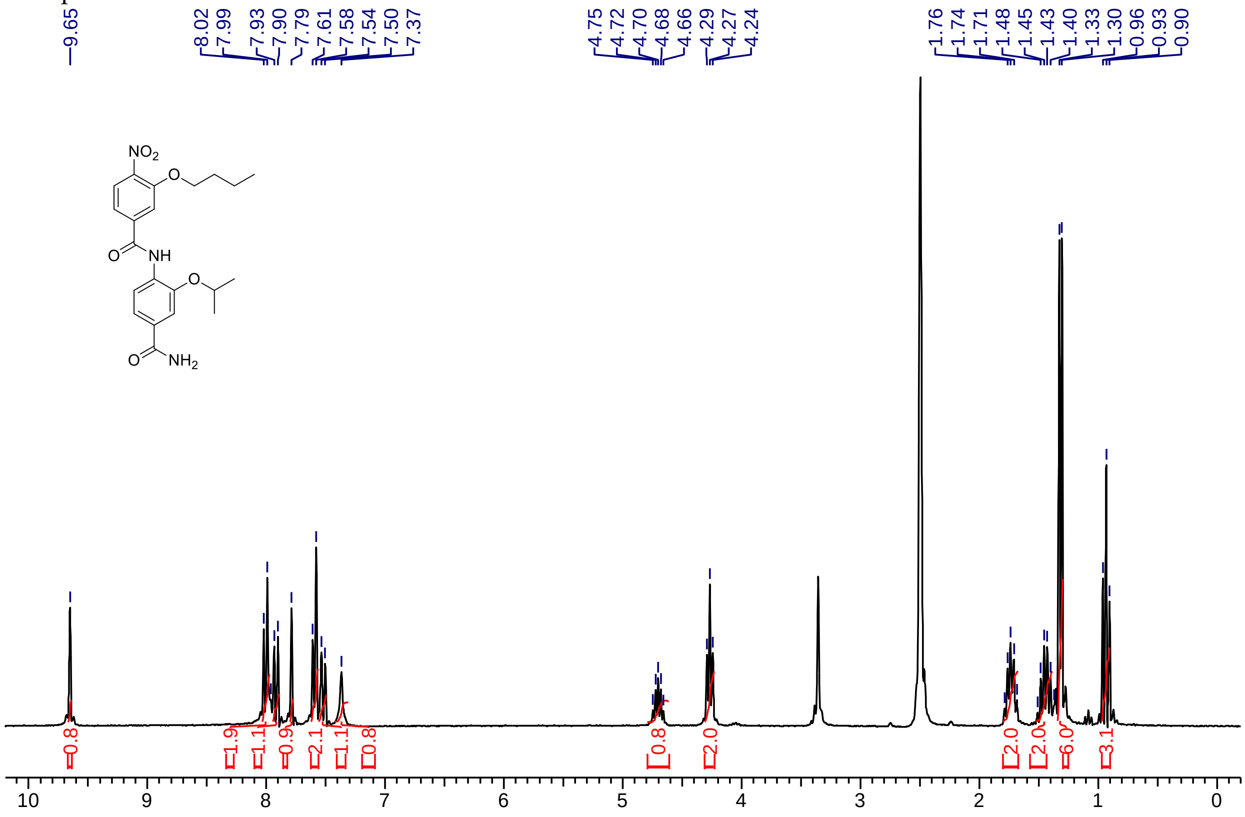

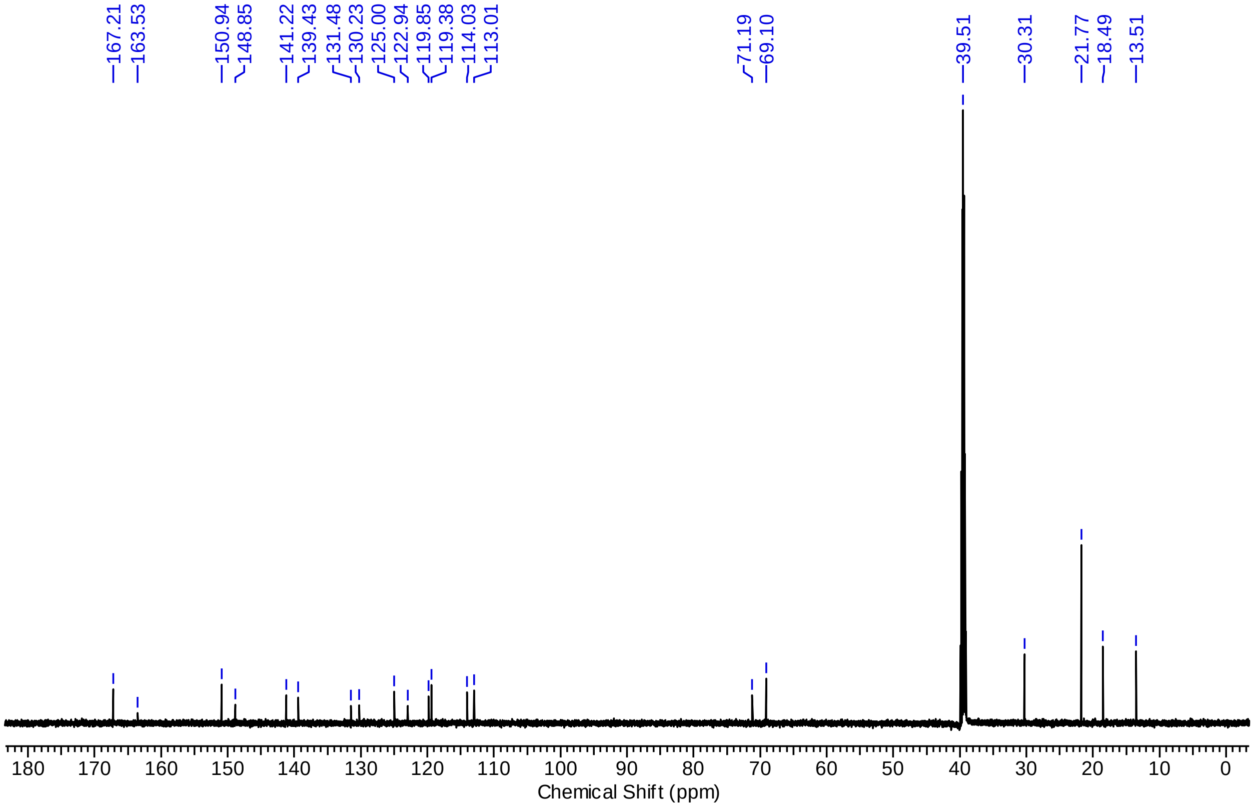

Compound **14i**

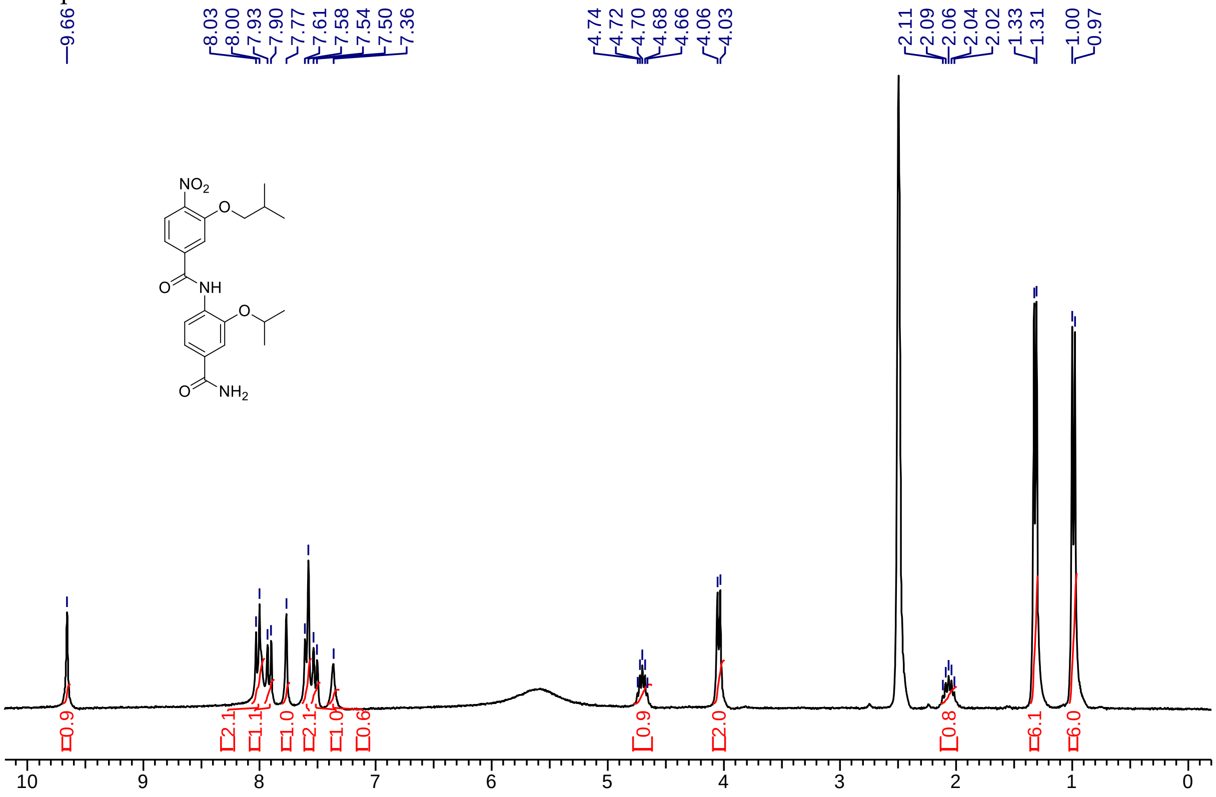

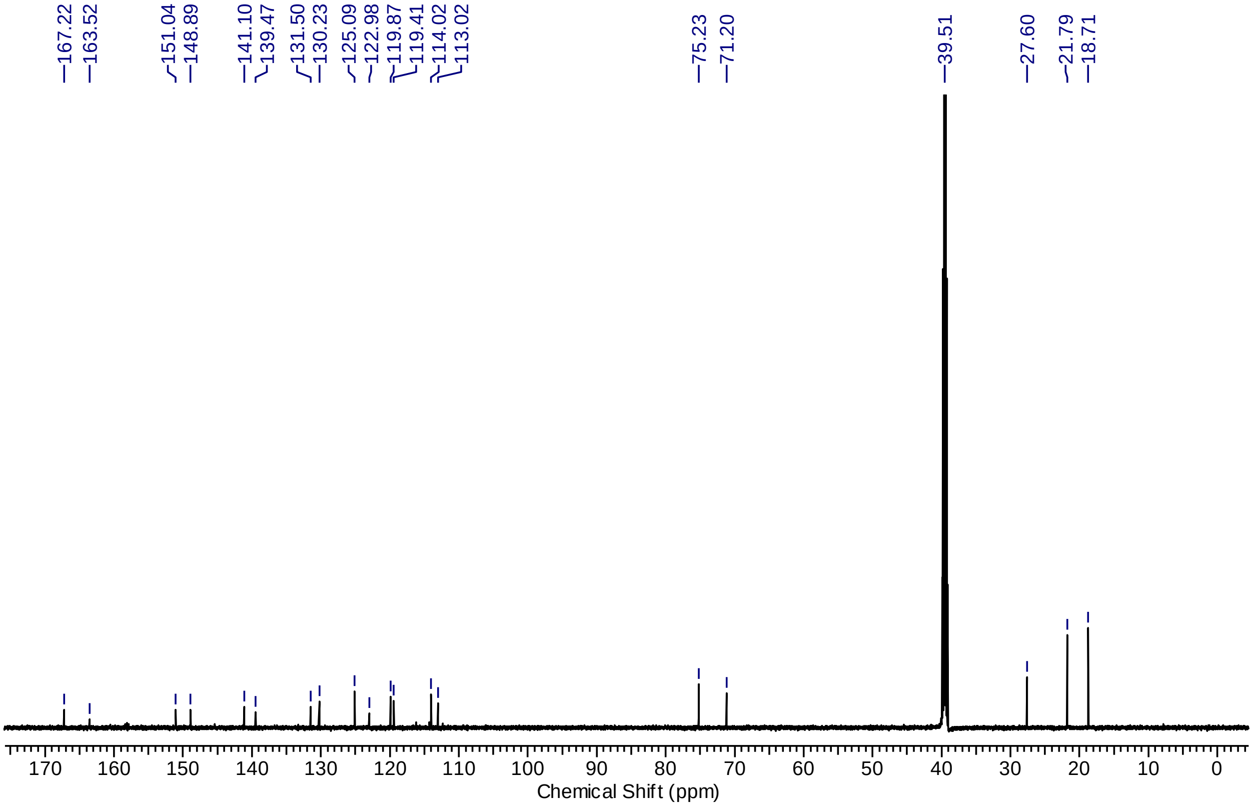

Compound **14j**

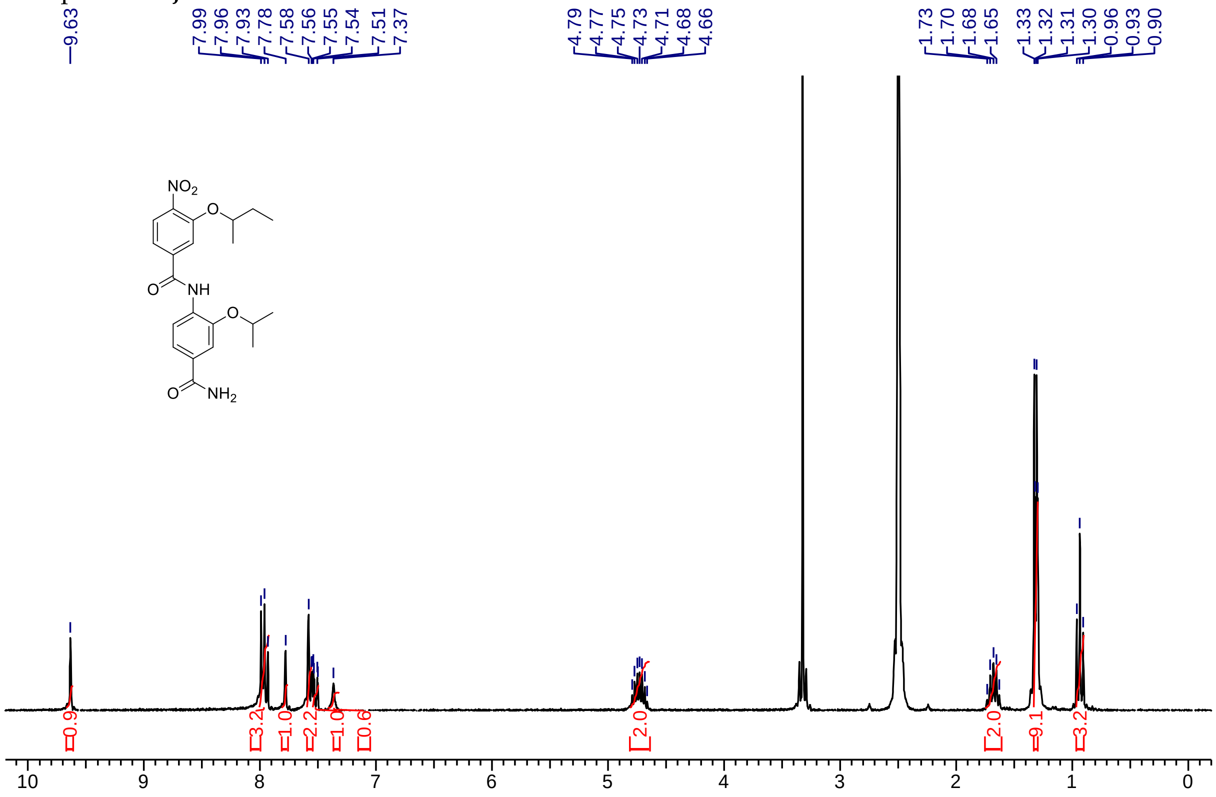

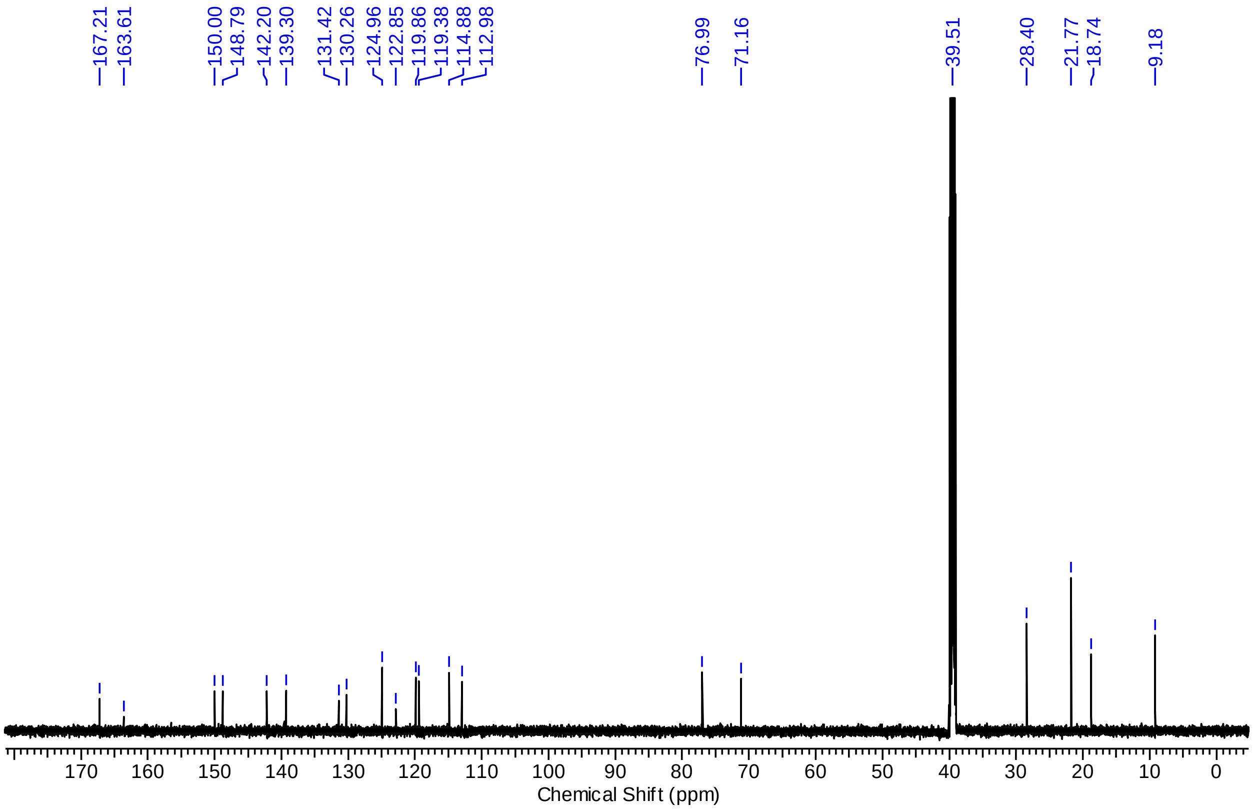

Compound **14k**

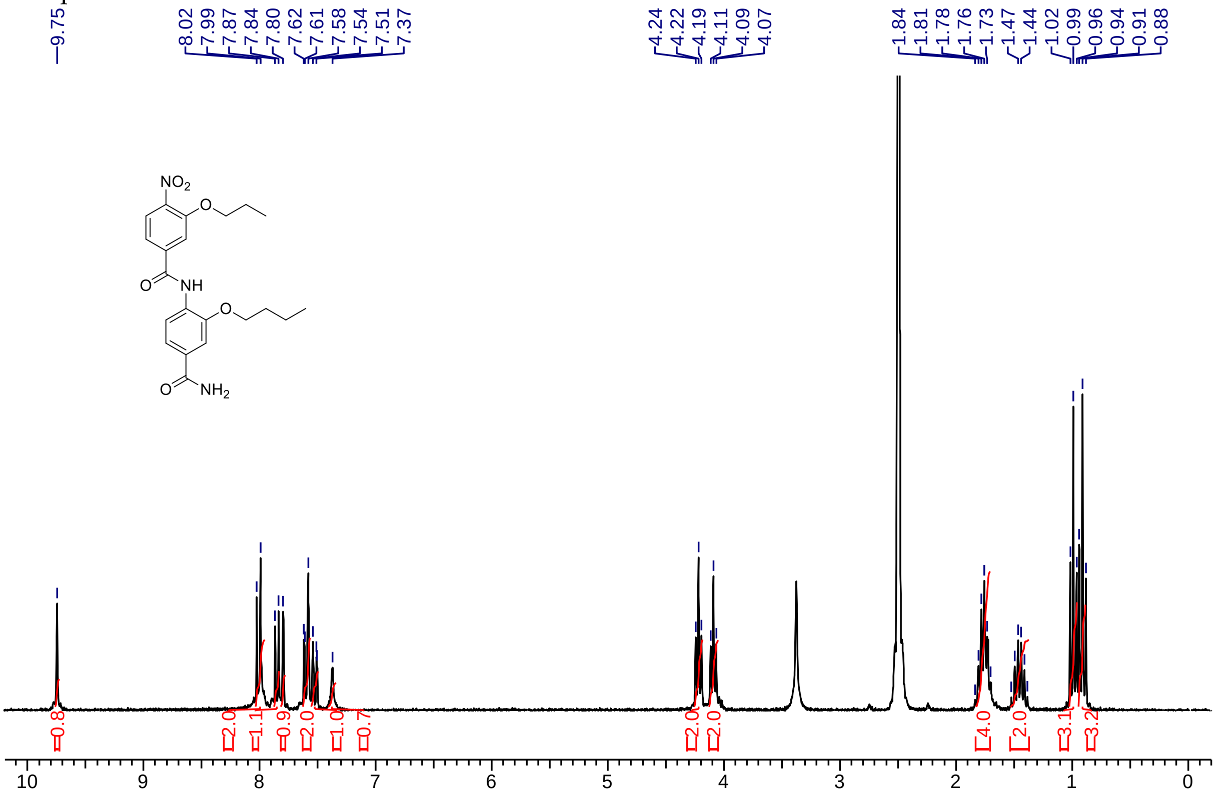

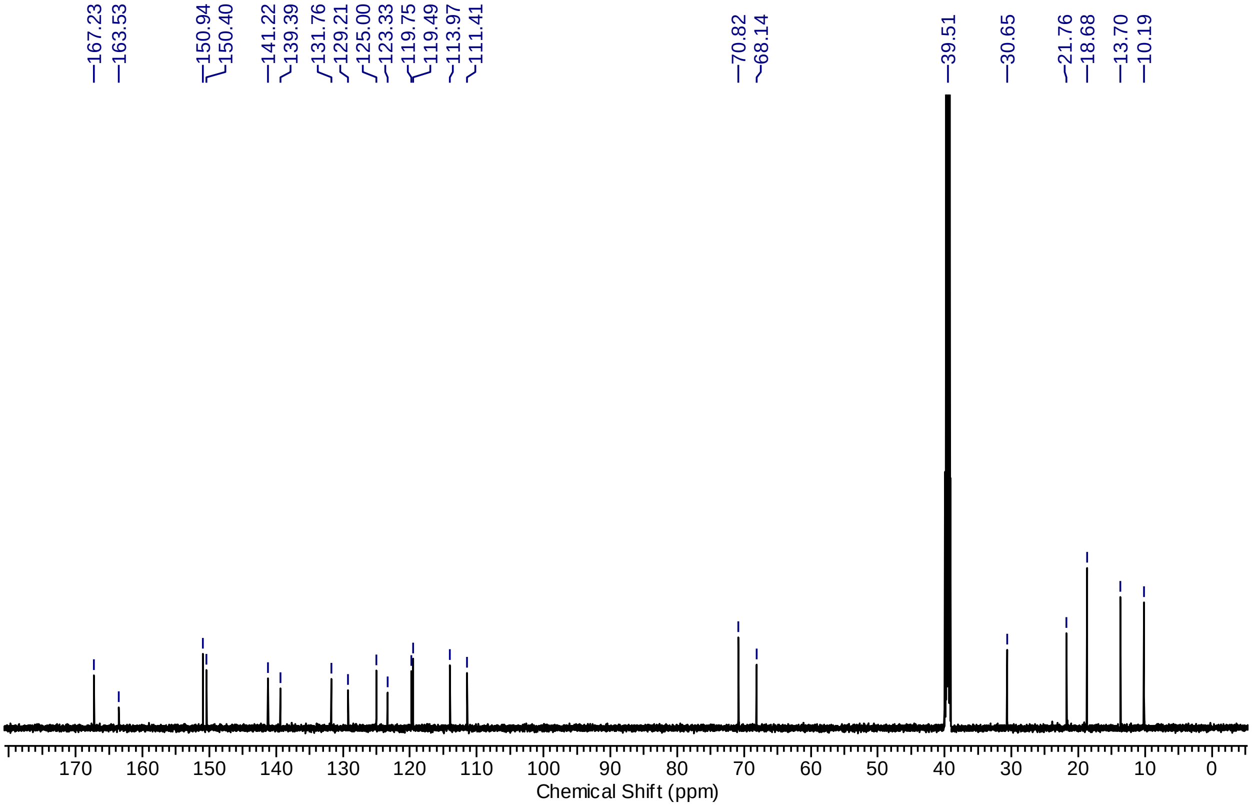

Compound **14l**

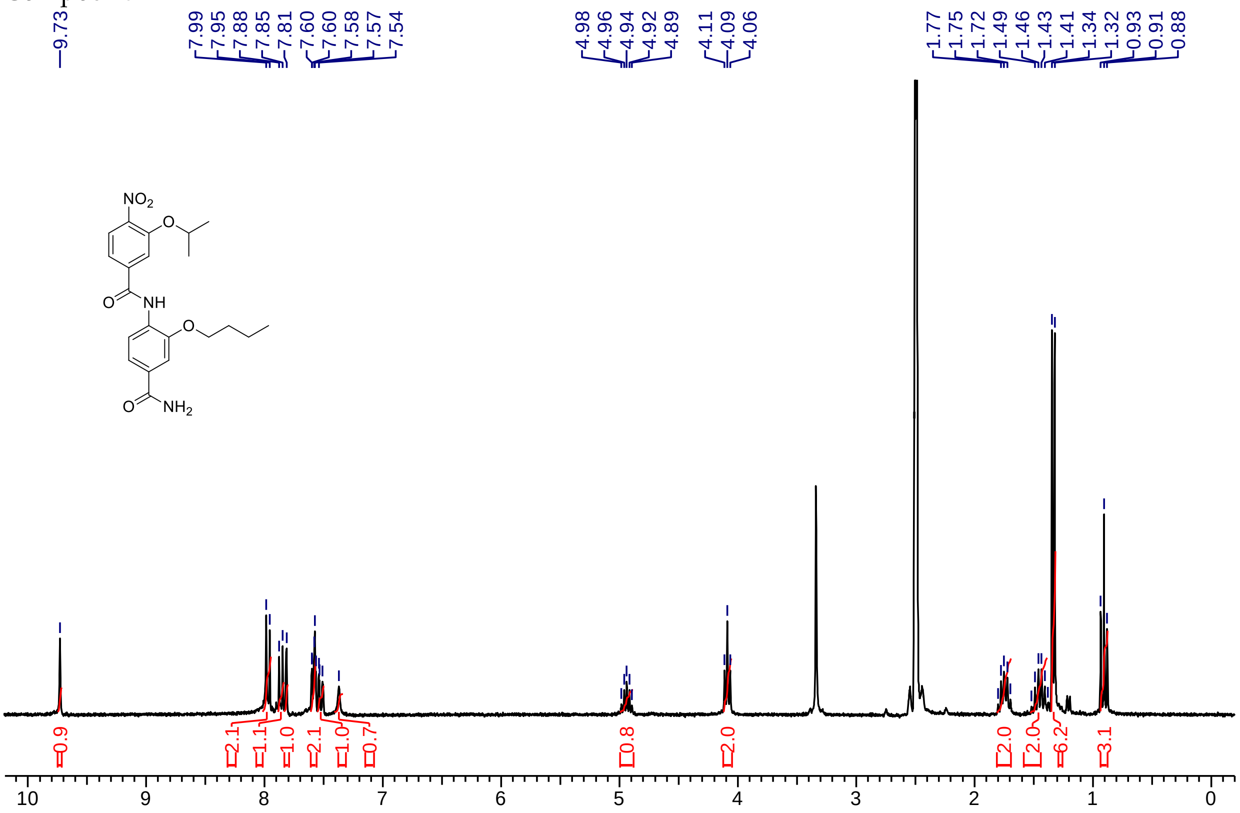

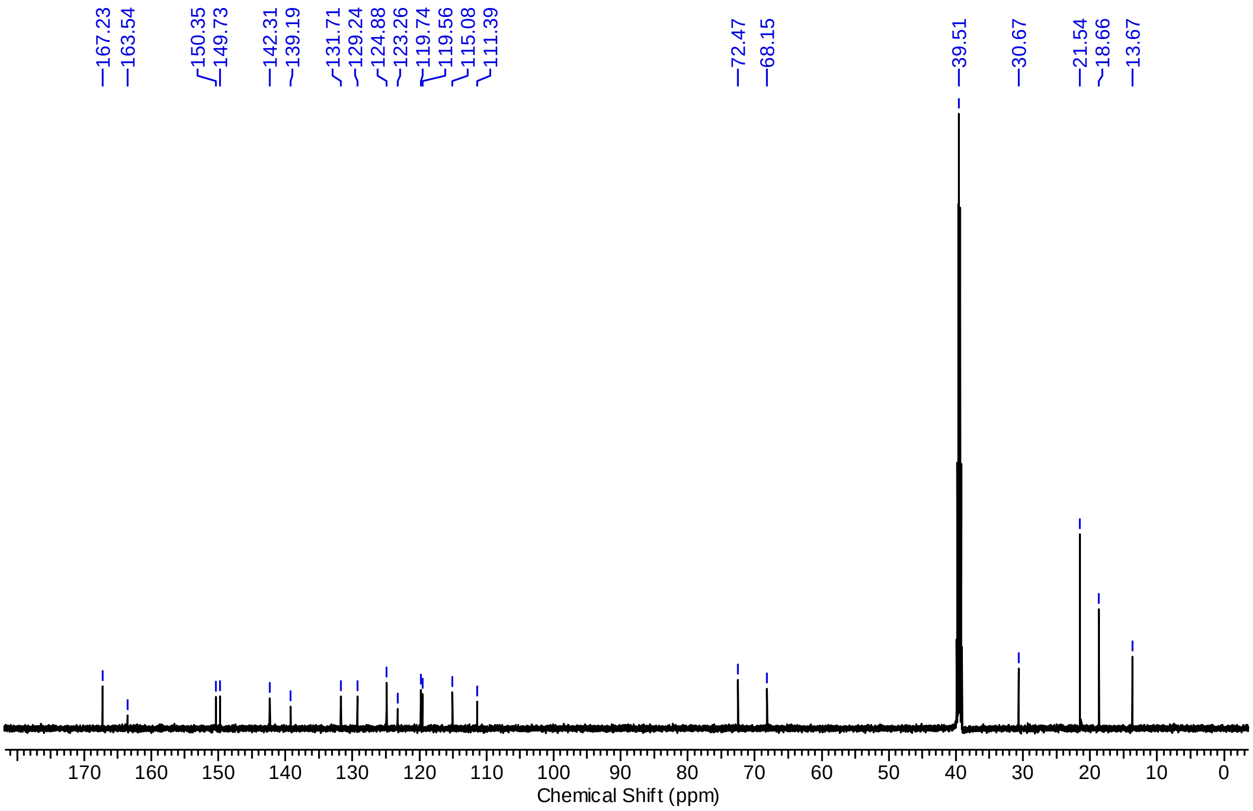

Compound **14m**

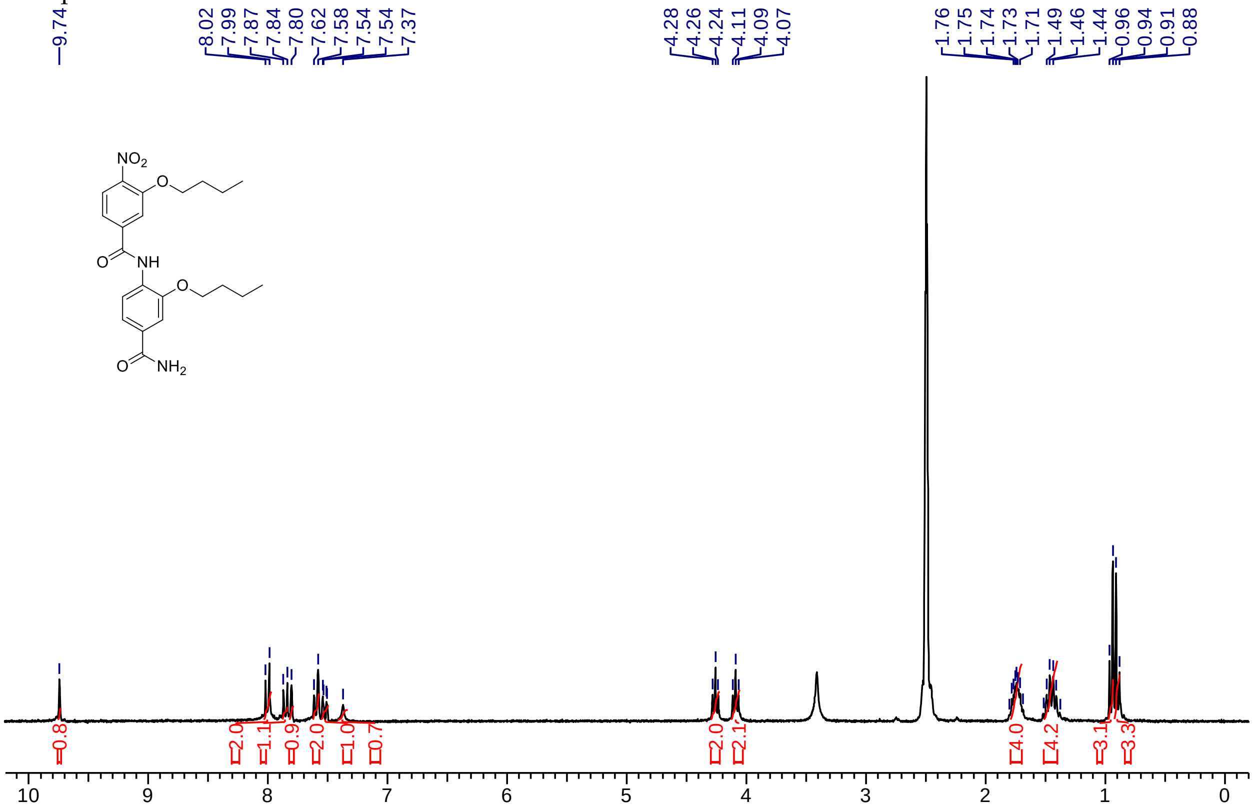

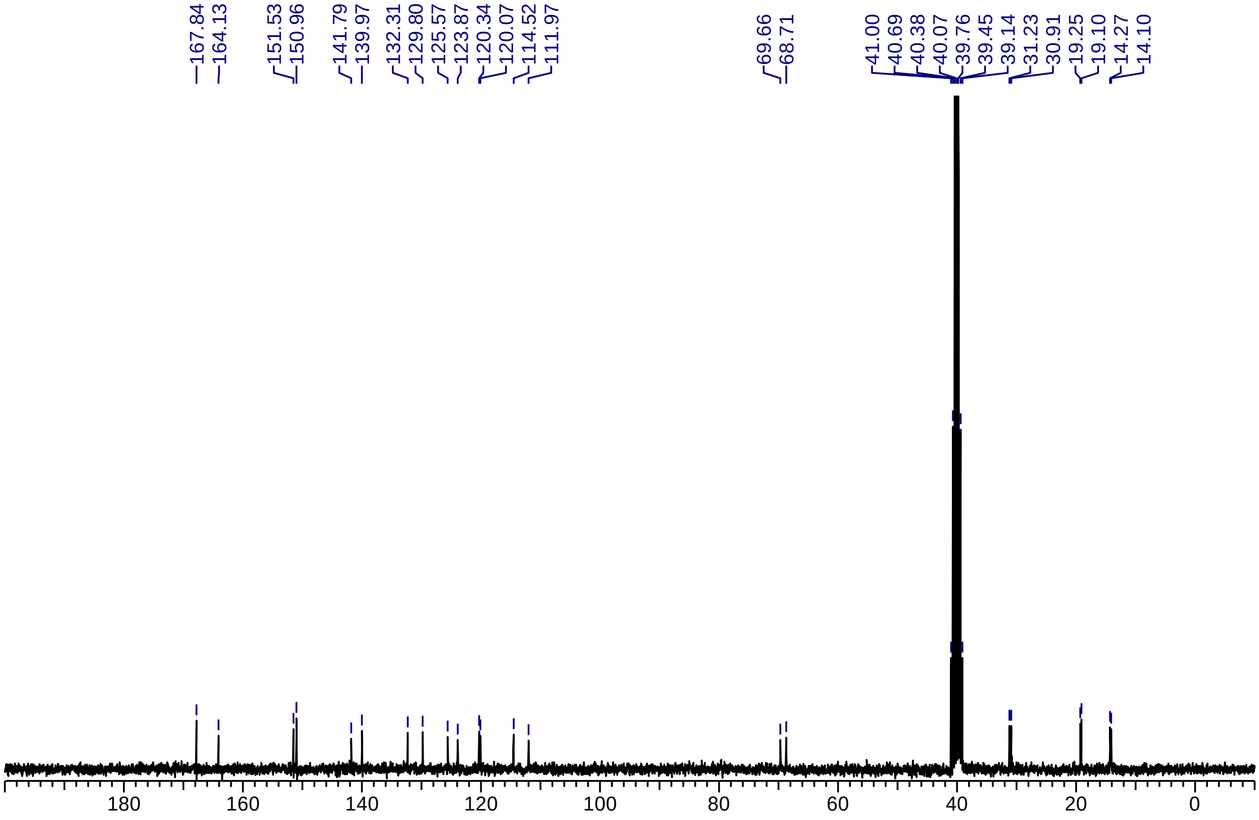

Compound **14n**

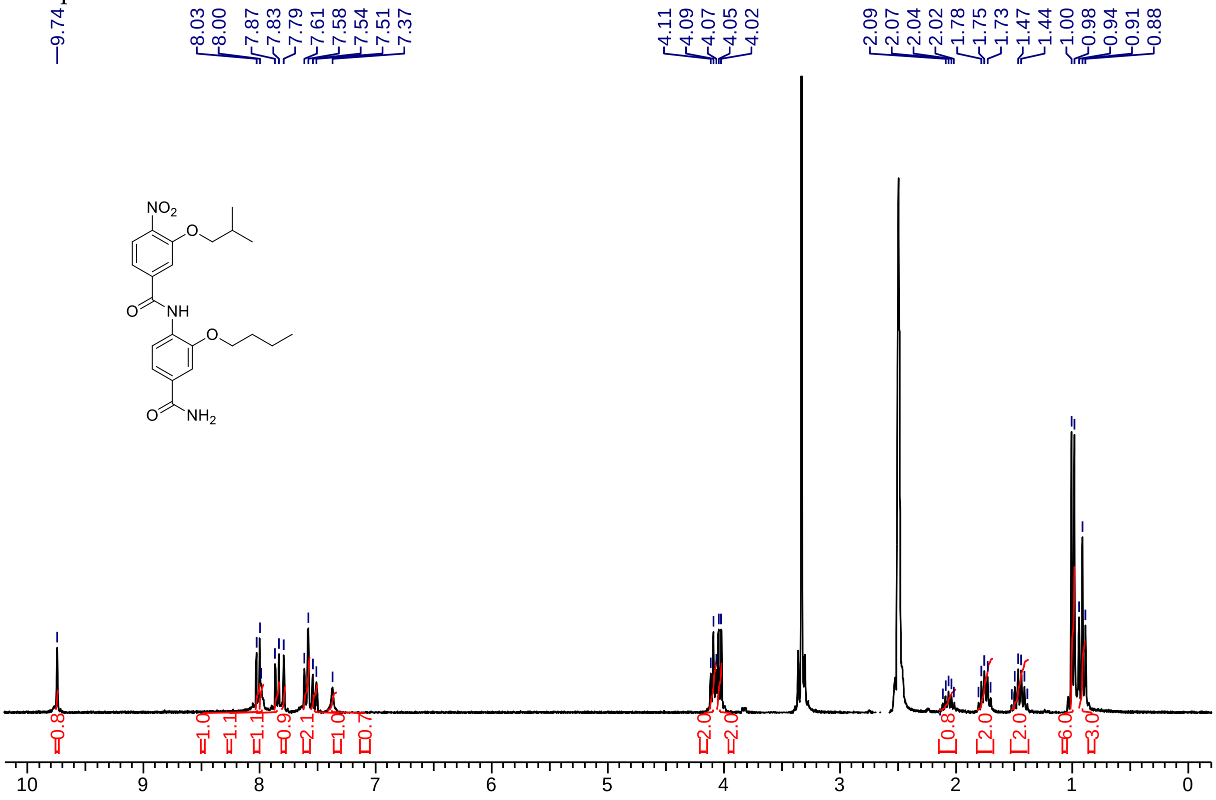

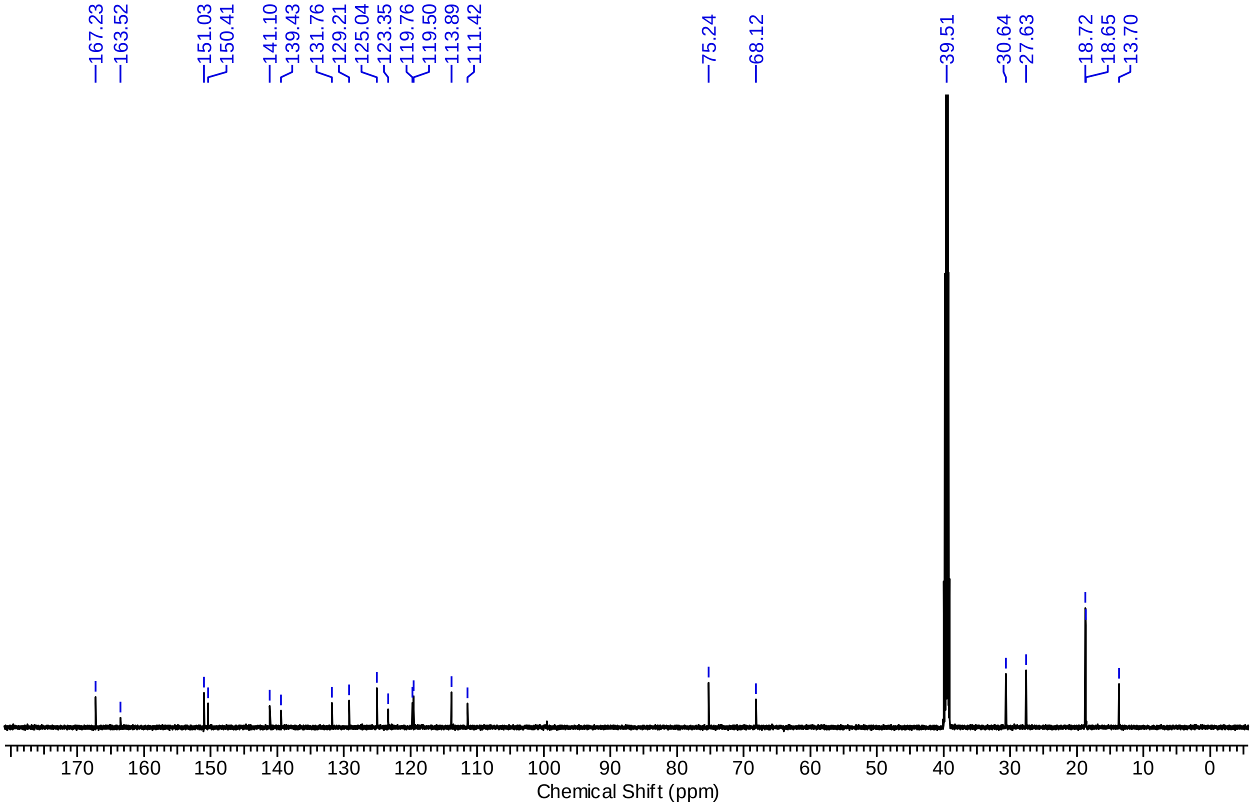

Compound **14o**

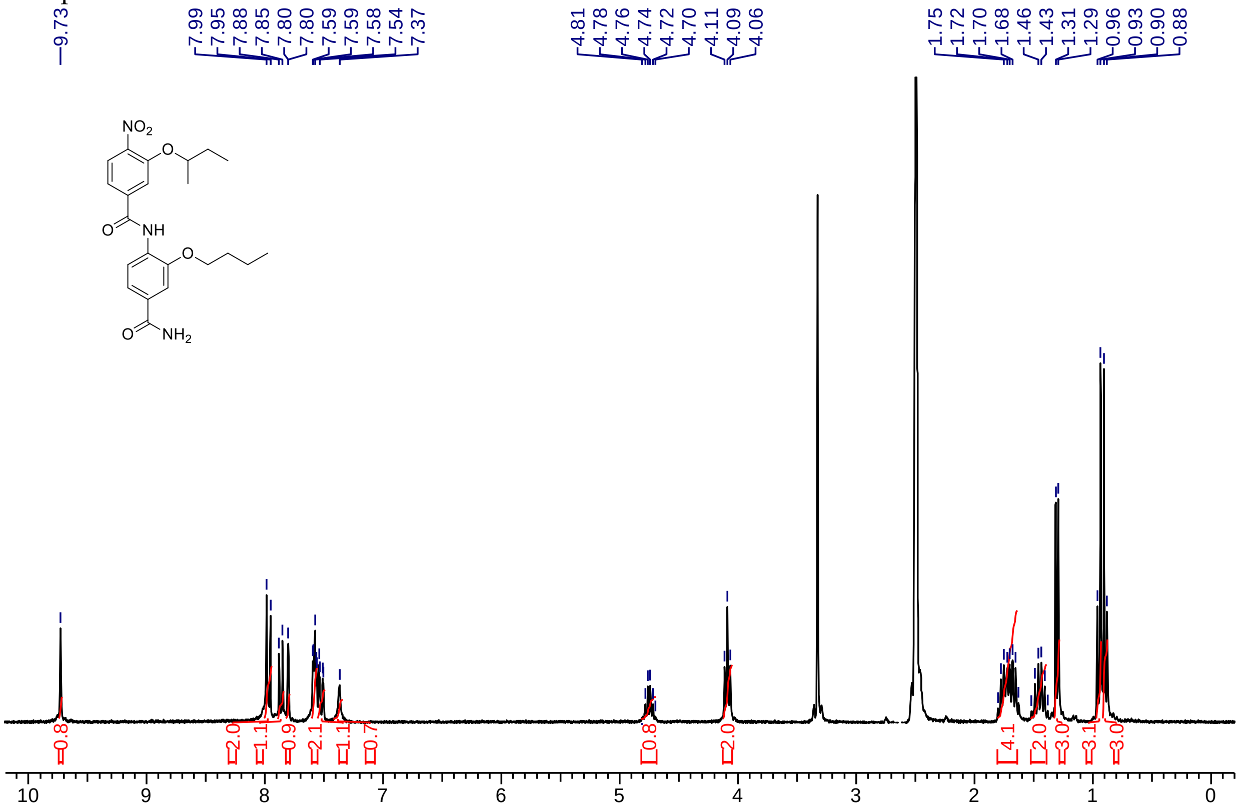

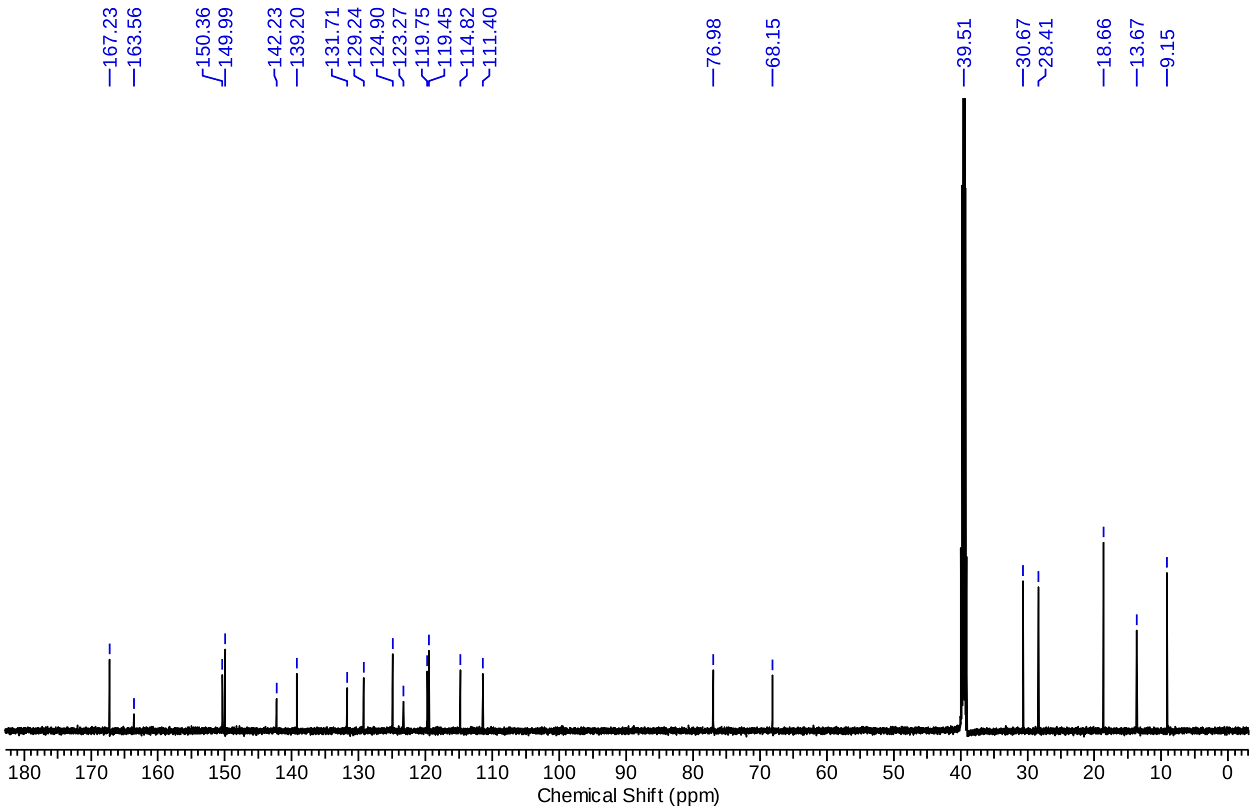

Compound **14p**

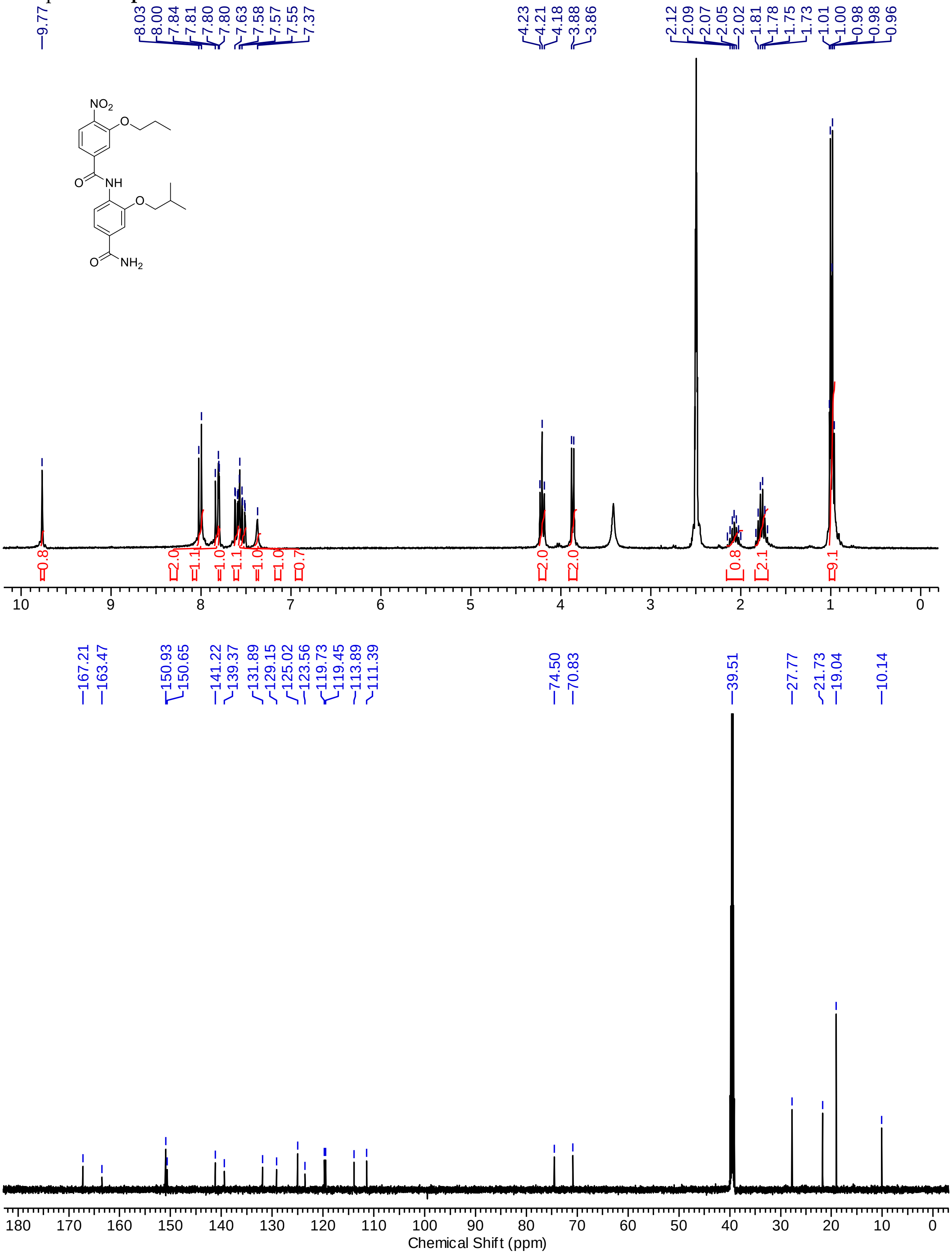

Compound **14q**

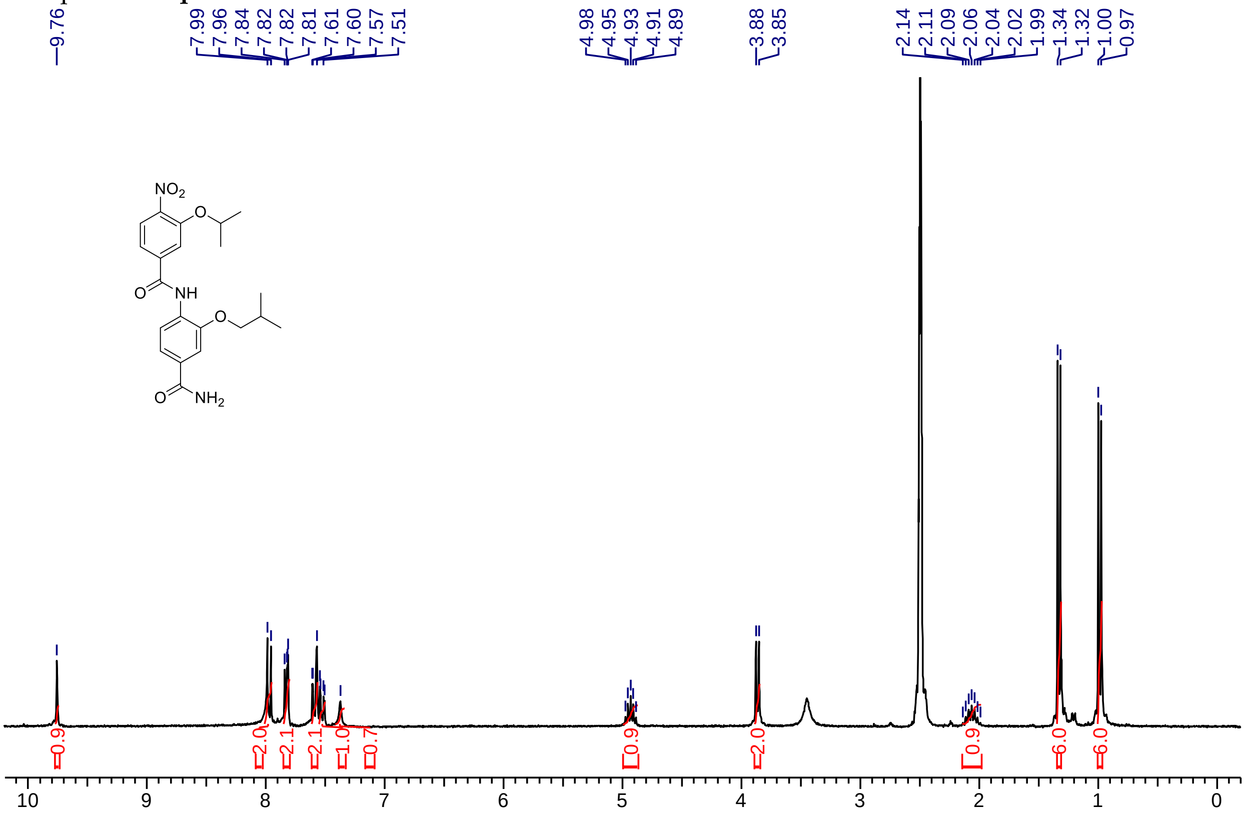

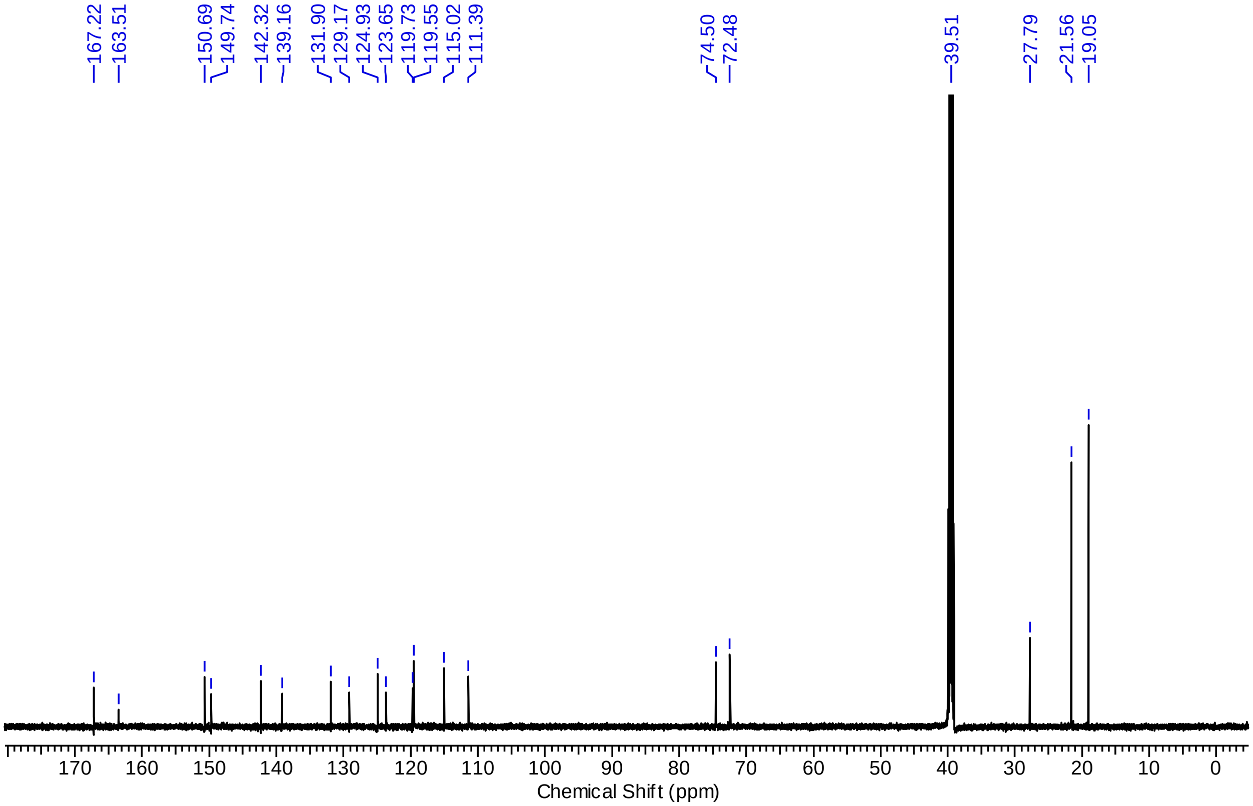

Compound **14r**

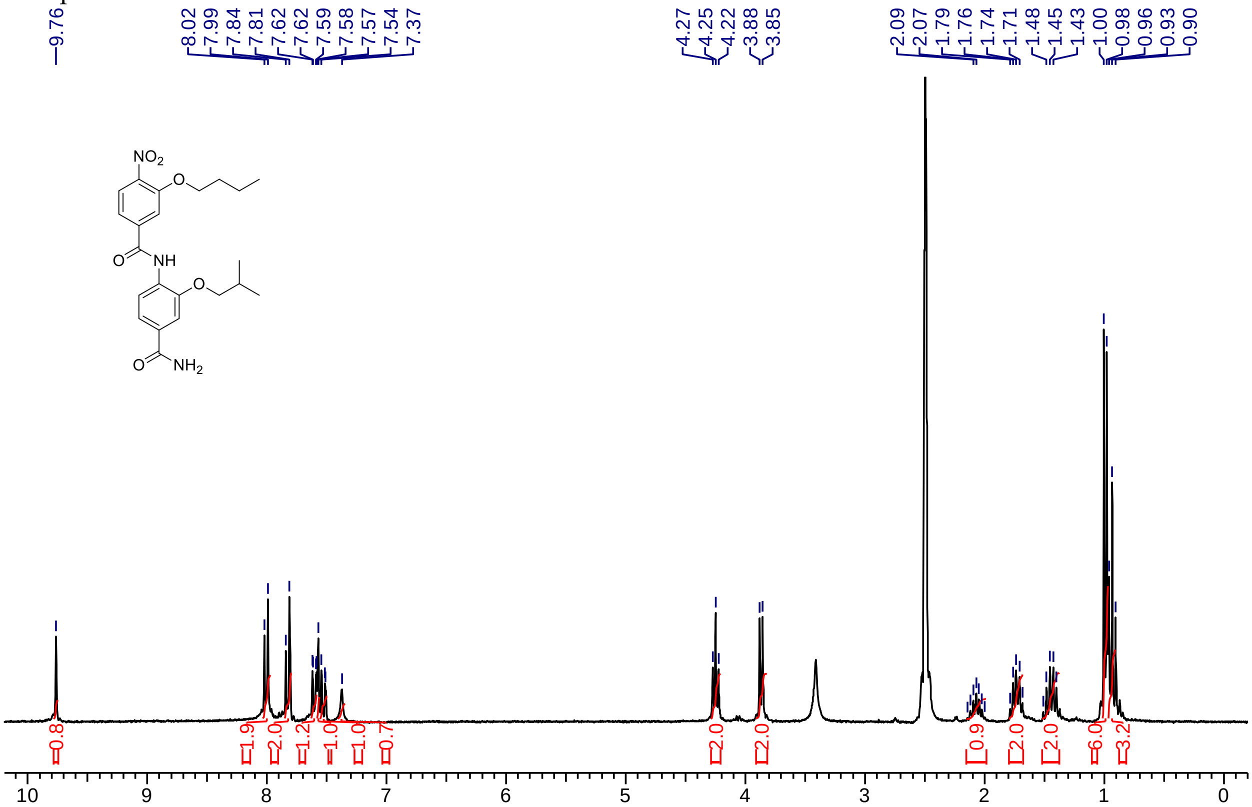

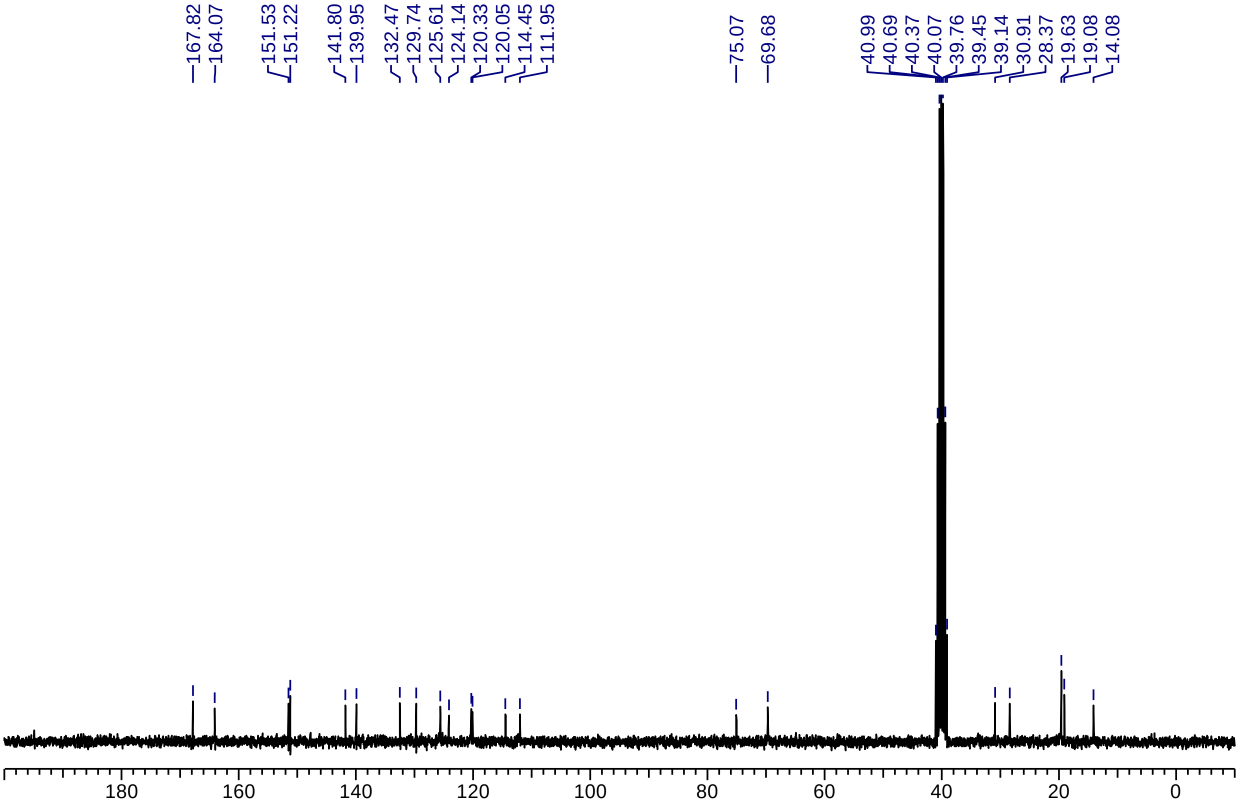

Compound **14s**

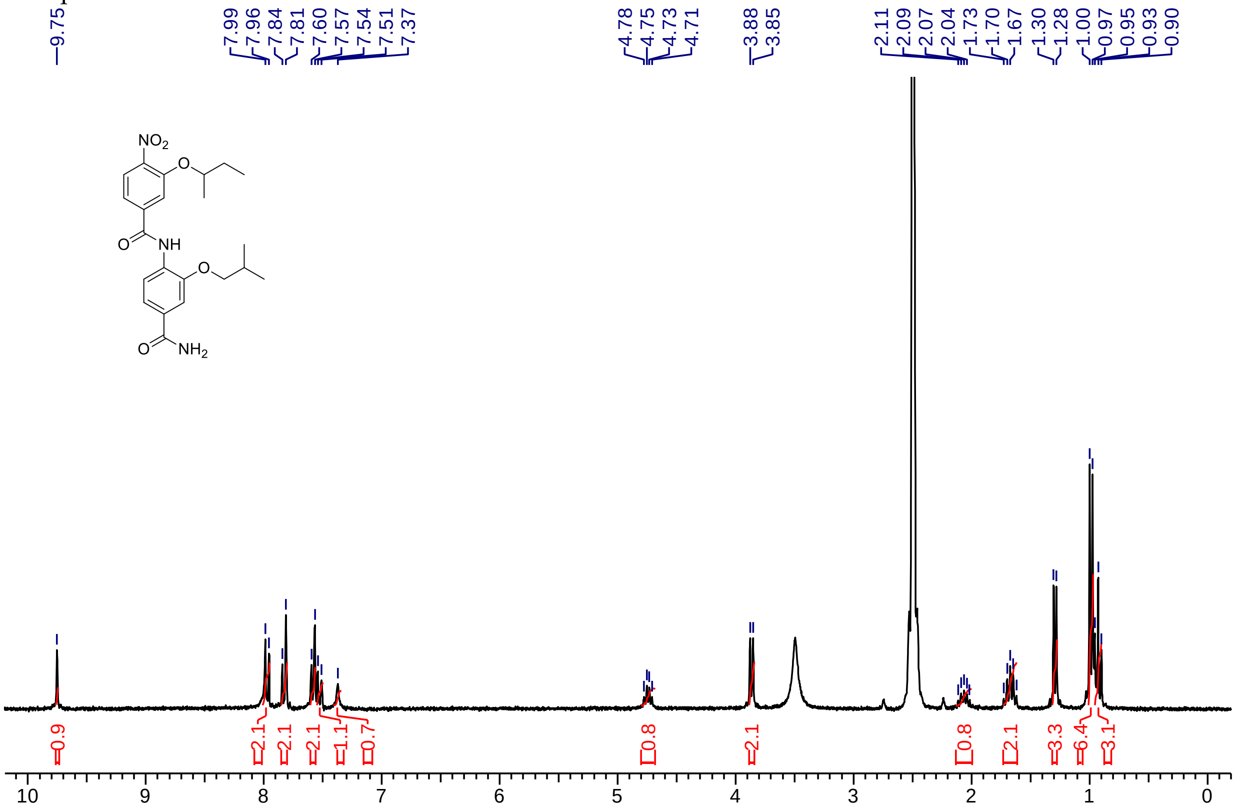

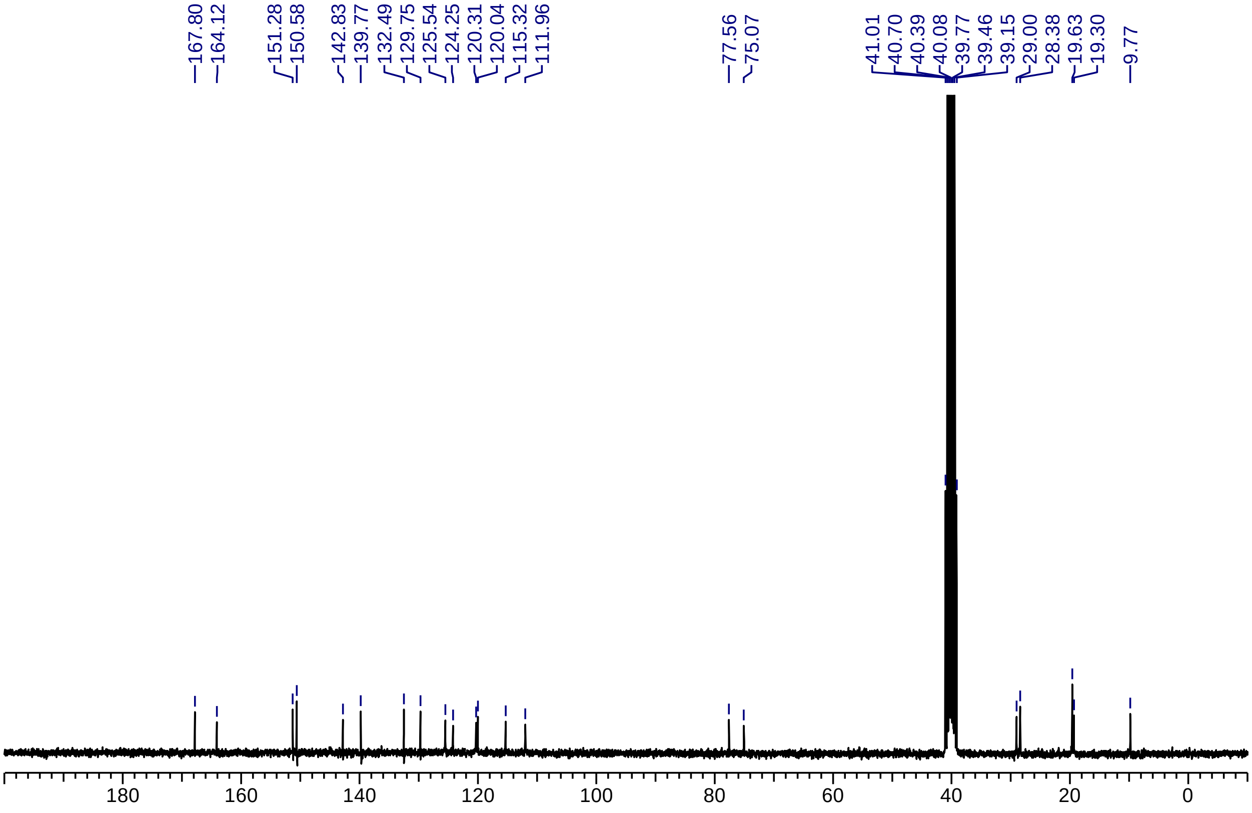

Compound **14t**

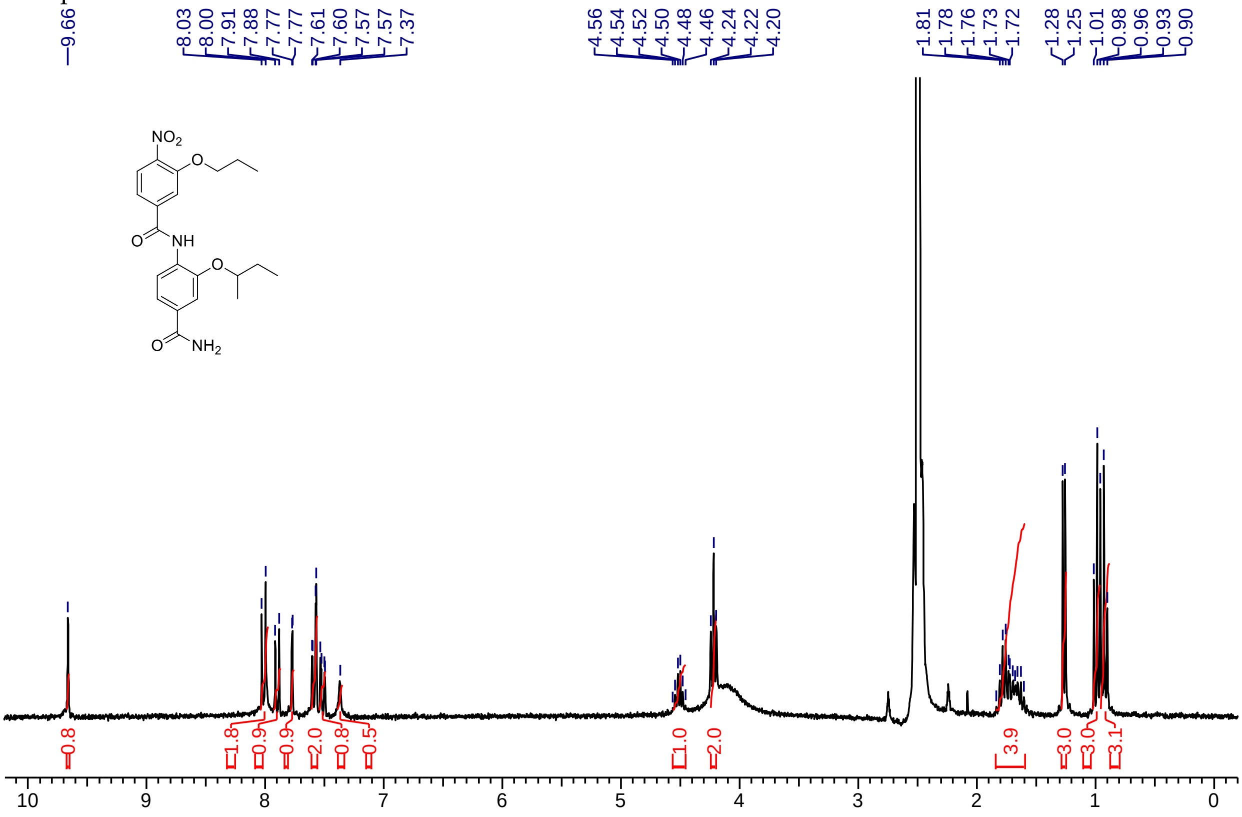

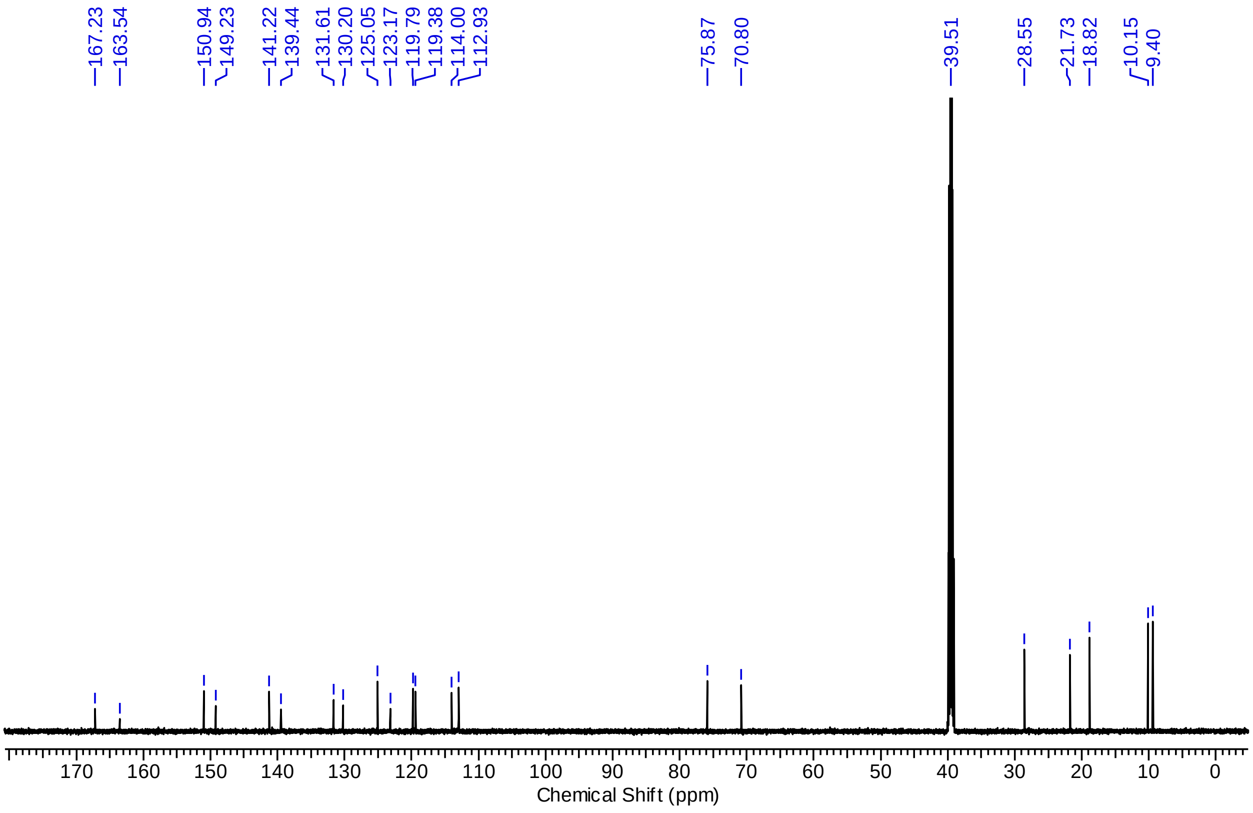

Compound **14u**

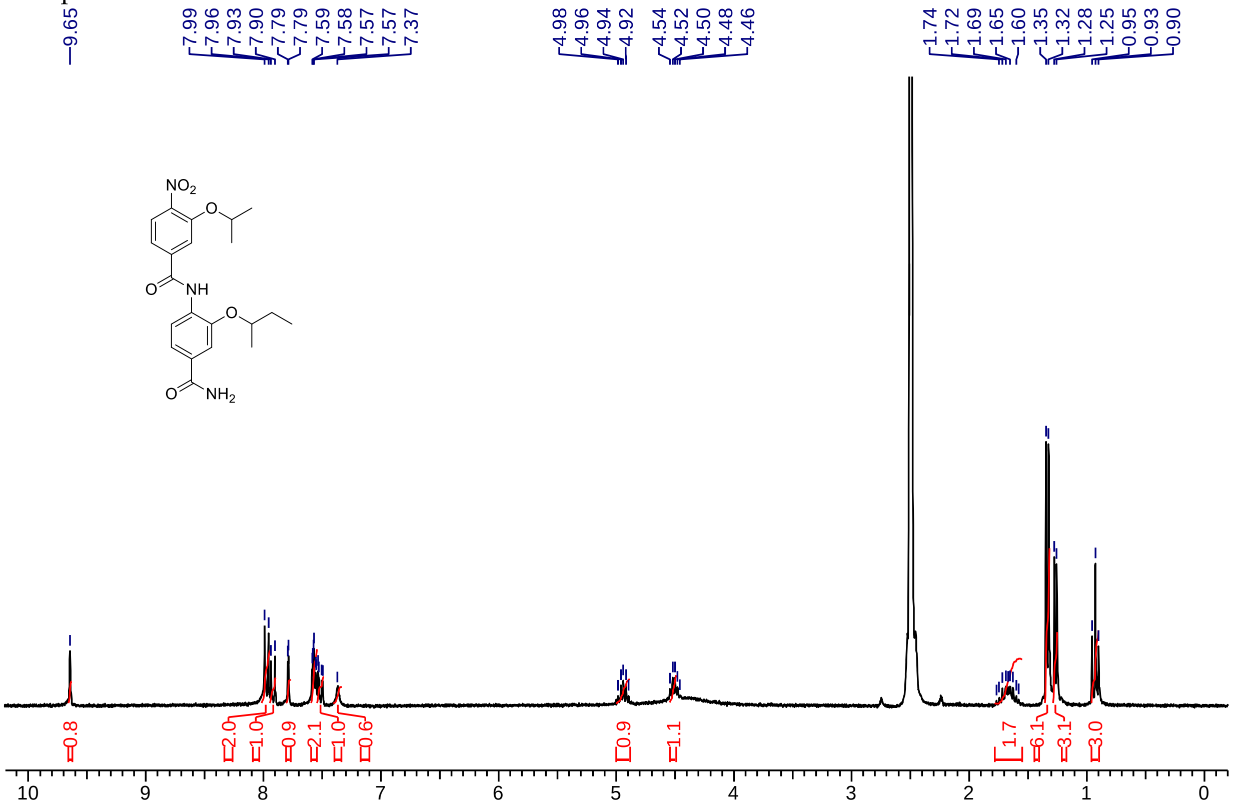

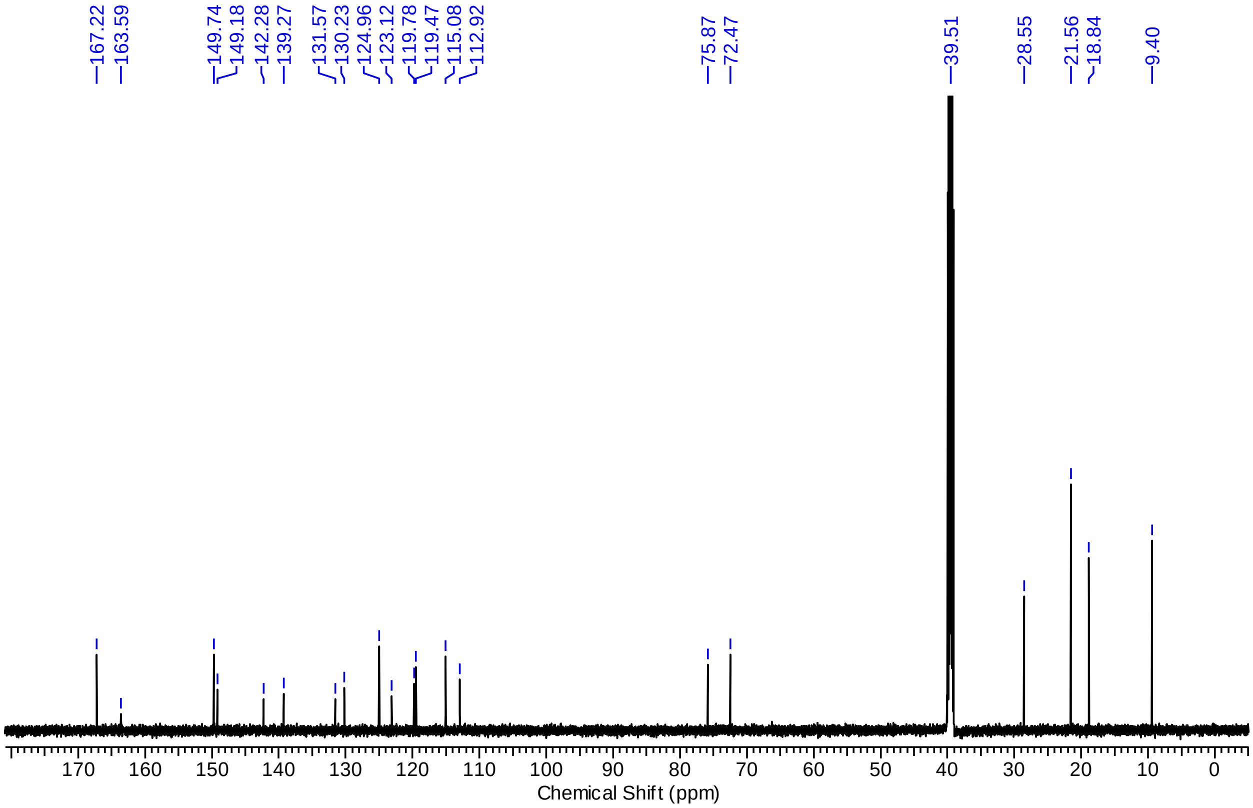

Compound **14v**

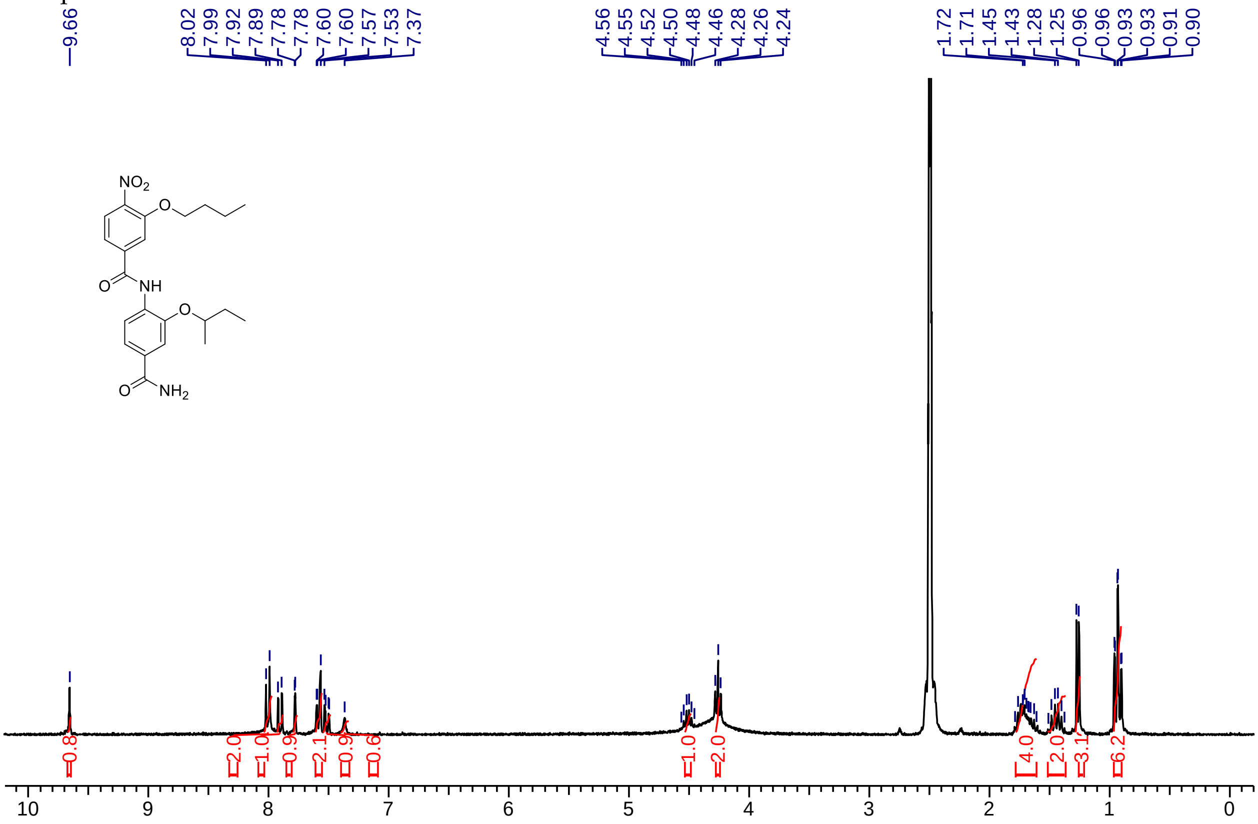

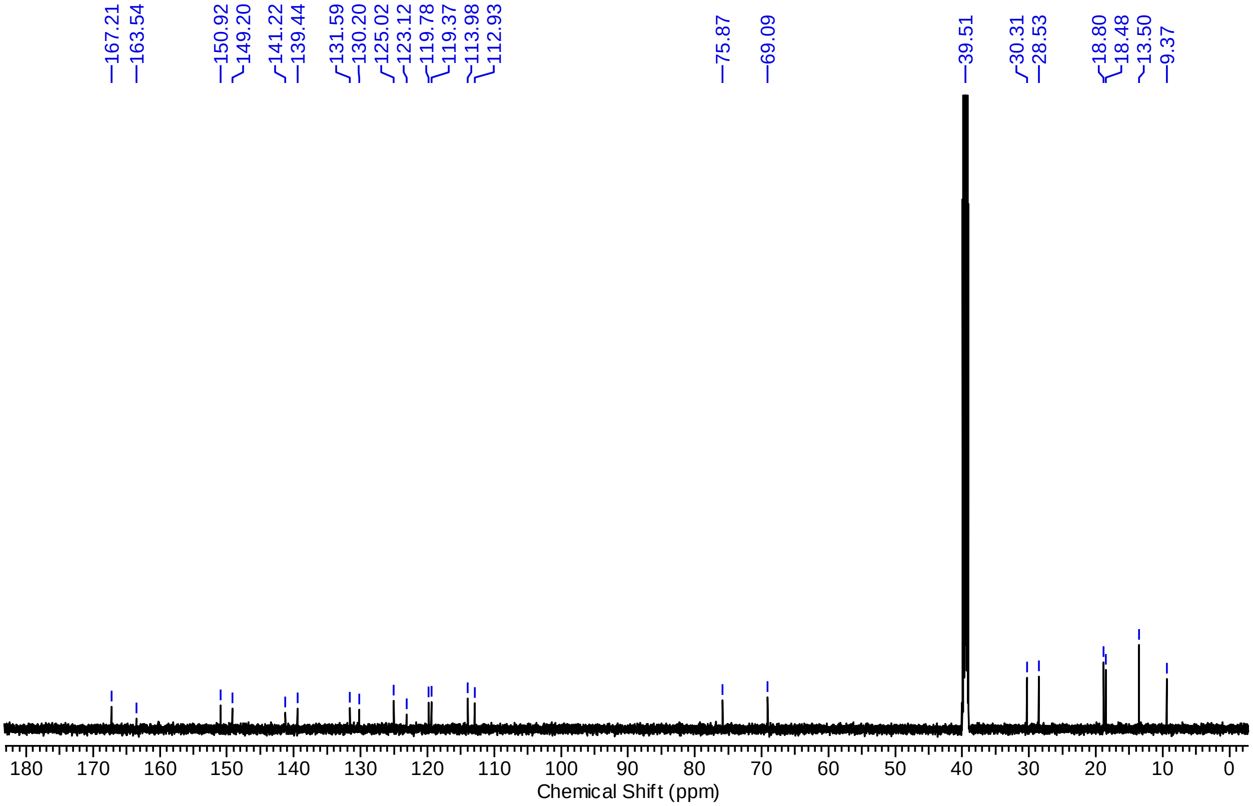

Compound **14w**

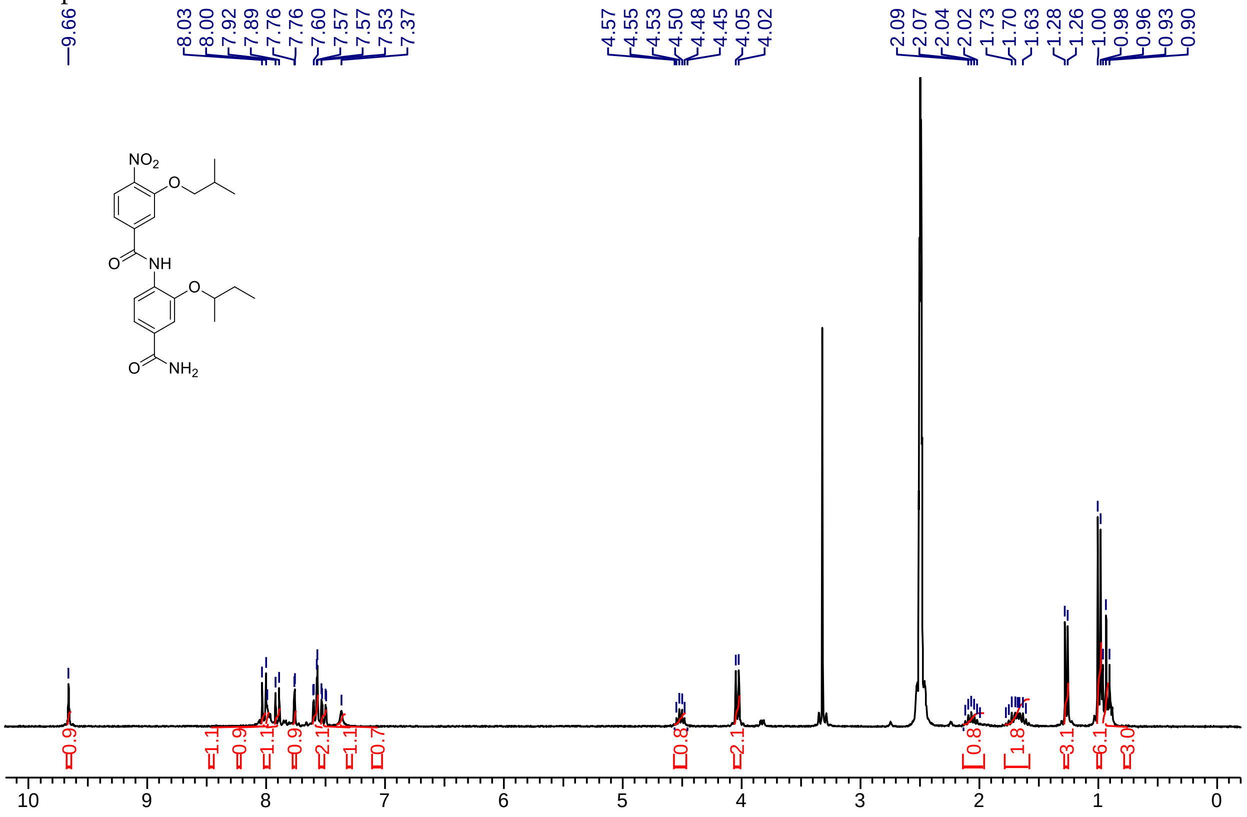

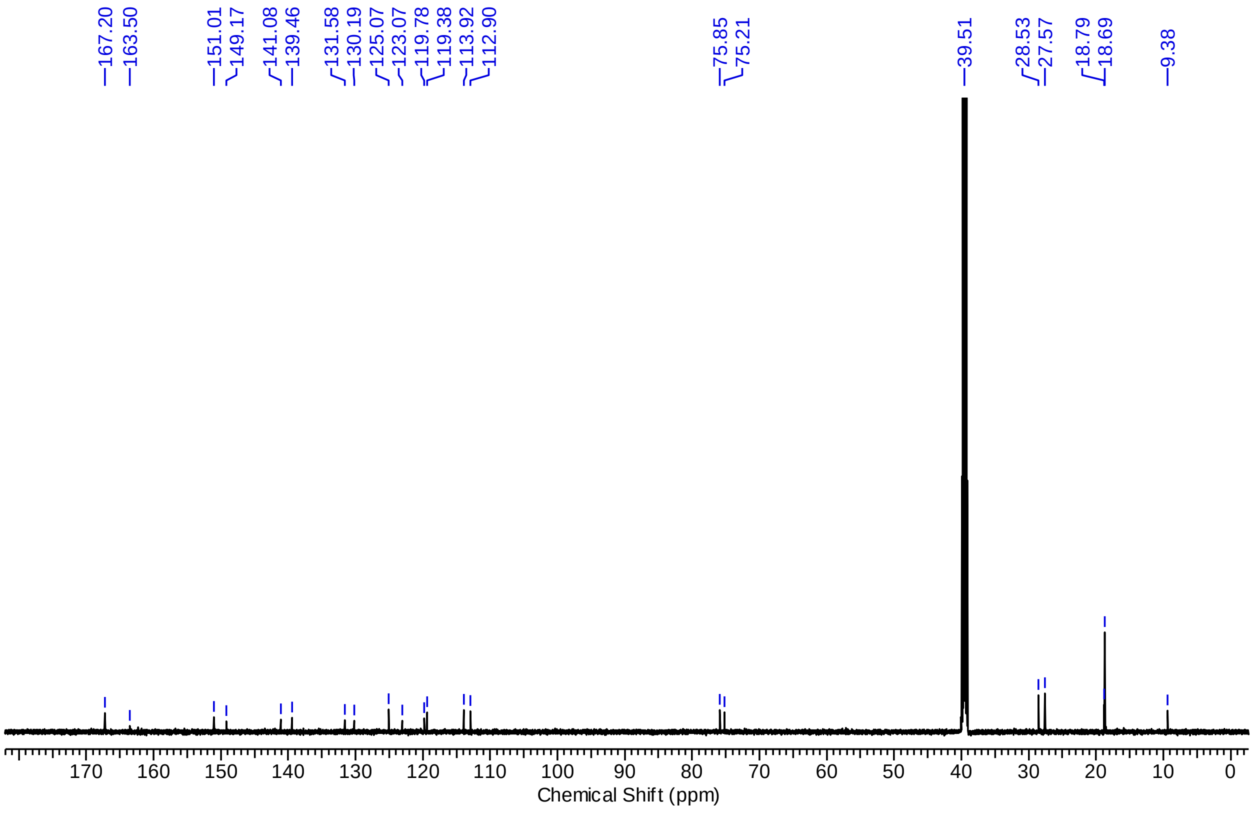

Compound **14x**

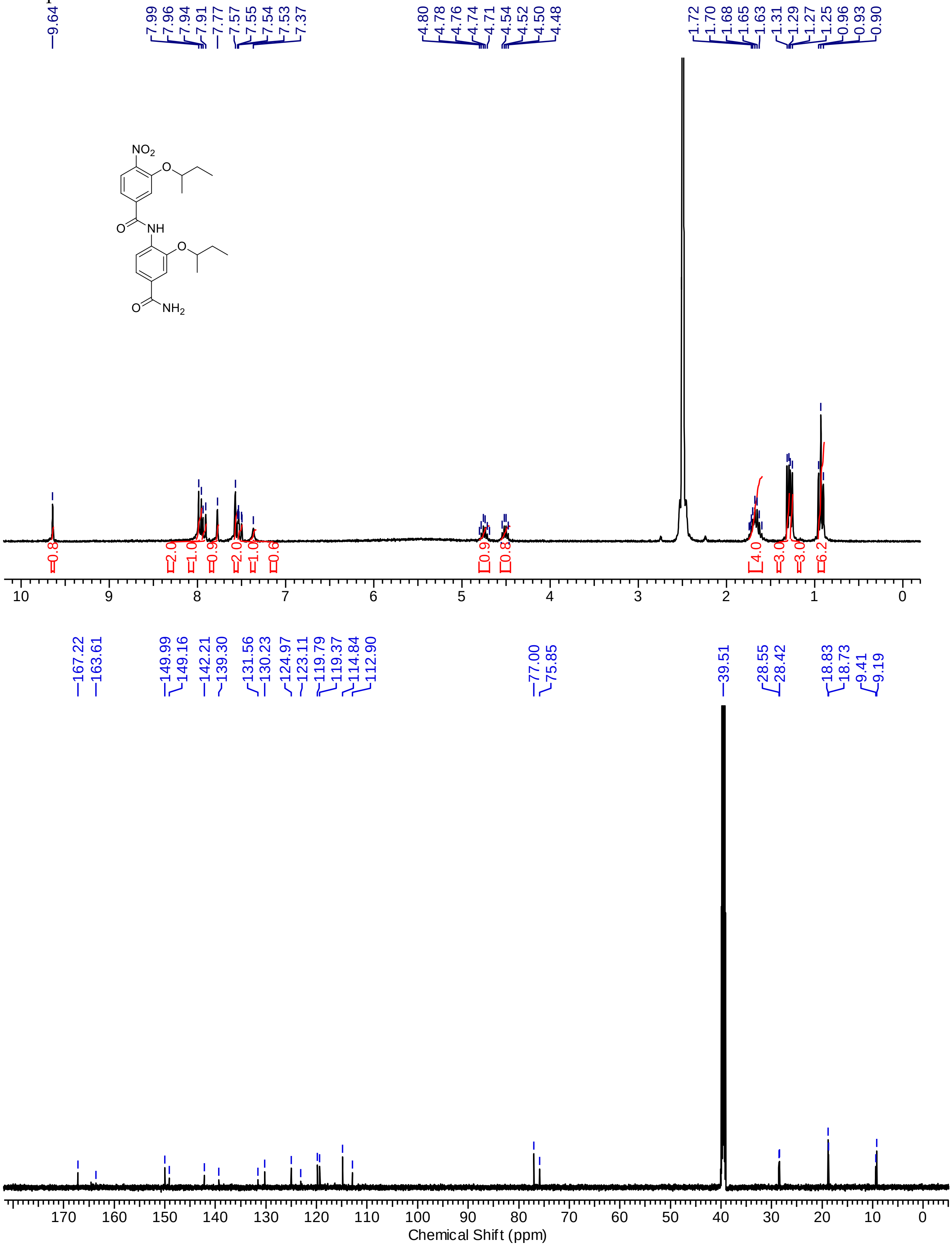

HPLC chromatograms of bis-benzamide library **14**

HPLC analysis: a linear gradient of B (10–90%) over 40 min (eluent A: H_2O/0.1% TFA; eluent B: CH_3CN) with a flow rate of 1.0 mL/min at 280 nm.

Compound **14a**

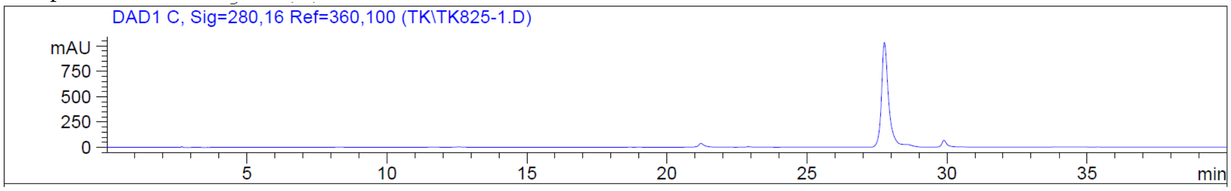

Compound **14b**

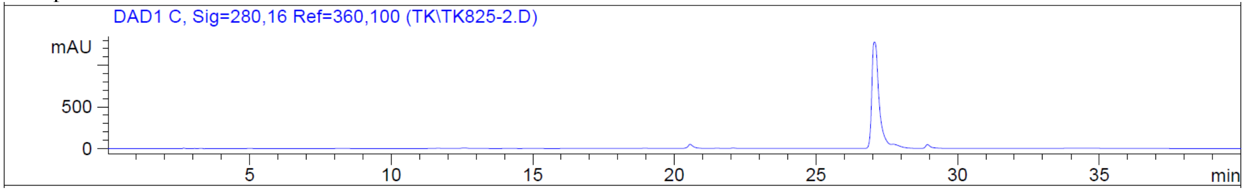

Compound **14c**

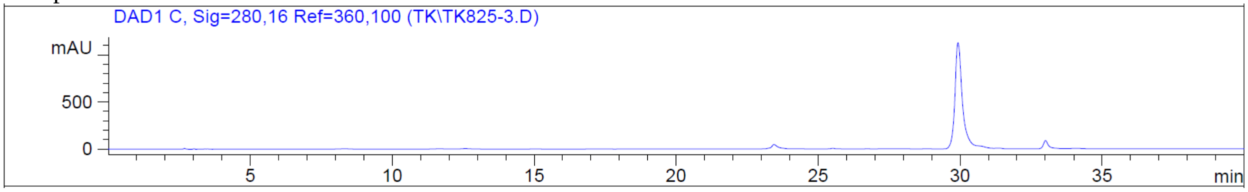

Compound **14d**

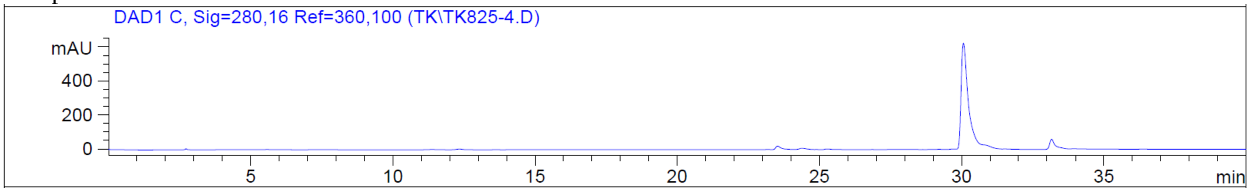

Compound **14e**

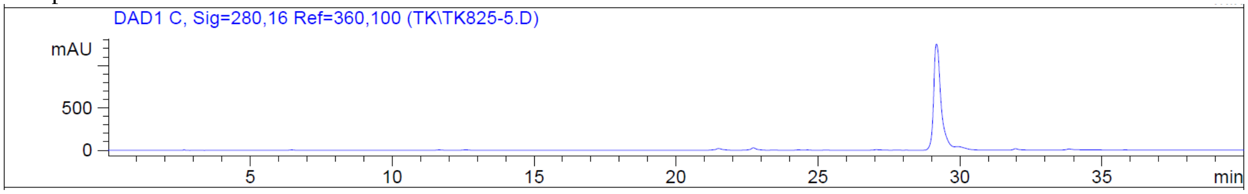

Compound **14f**

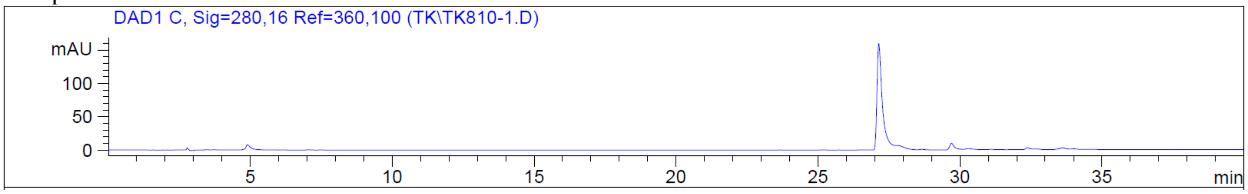

Compound **14g**

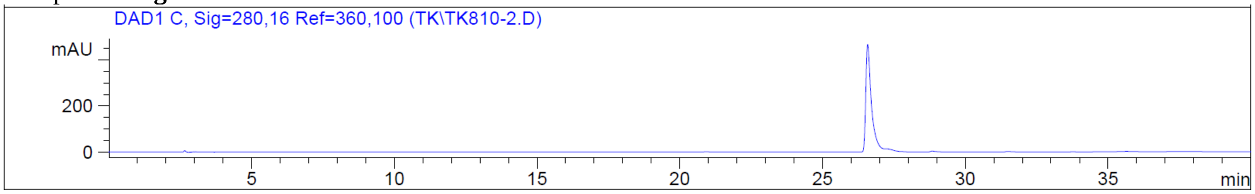

Compound **14h**

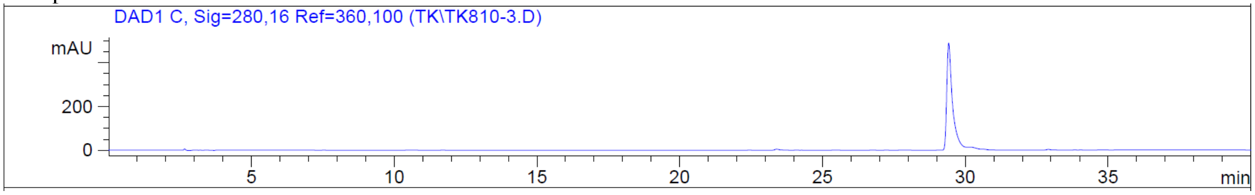

Compound **14i**

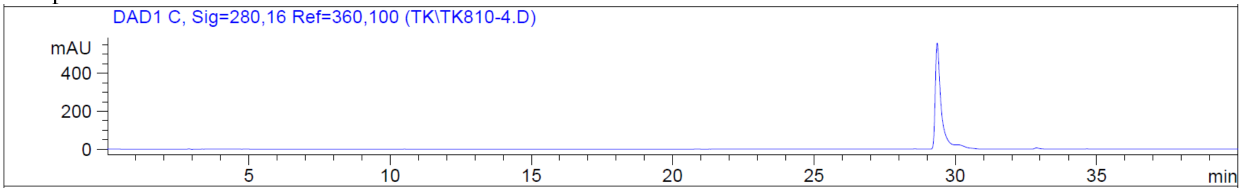

Compound **14j**

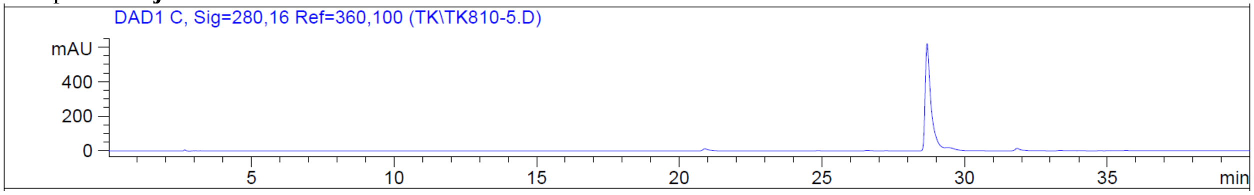

Compound **14k**

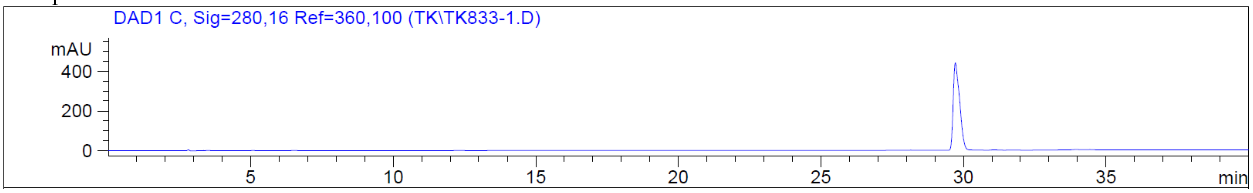

Compound **14l**

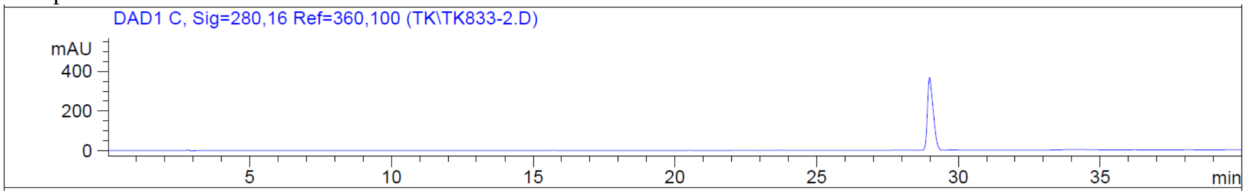

Compound **14m**

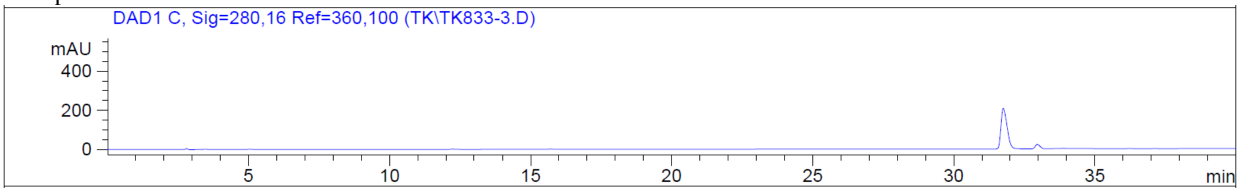

Compound **14n**

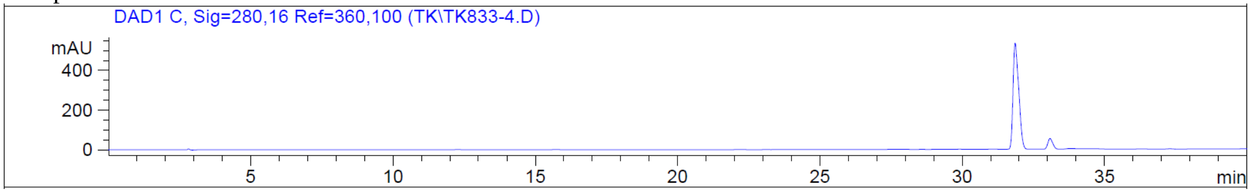

Compound **14o**

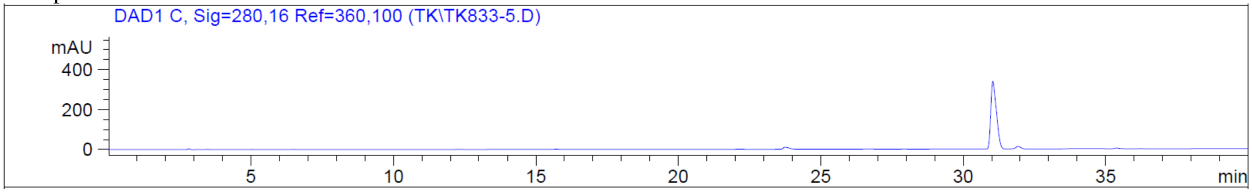

Compound **14p**

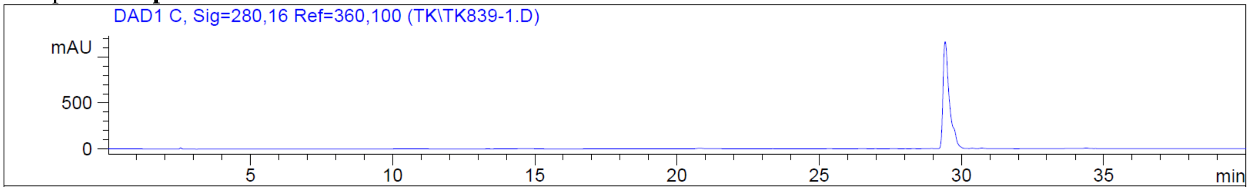

Compound **14q**

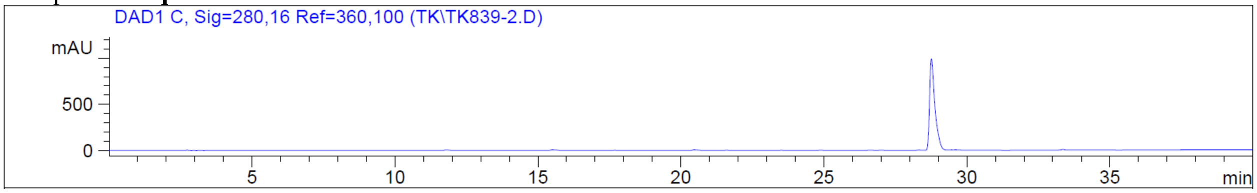

Compound **14r**

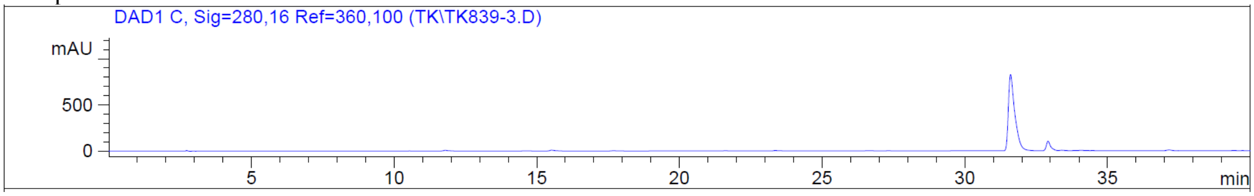

Compound **14s**

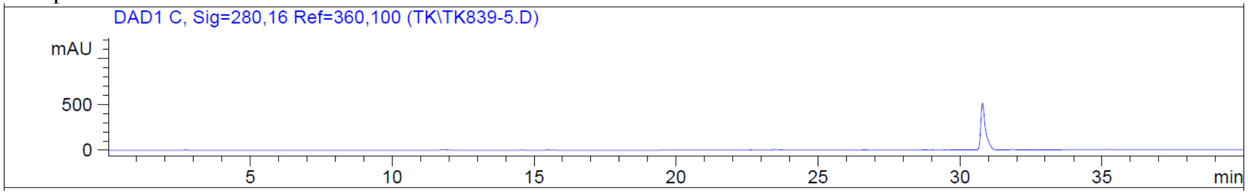

Compound **14t**

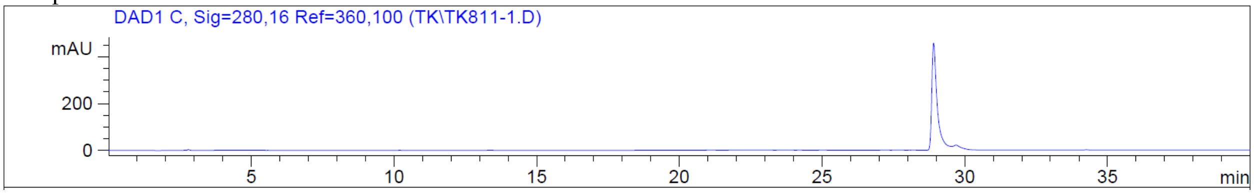

Compound **14u**

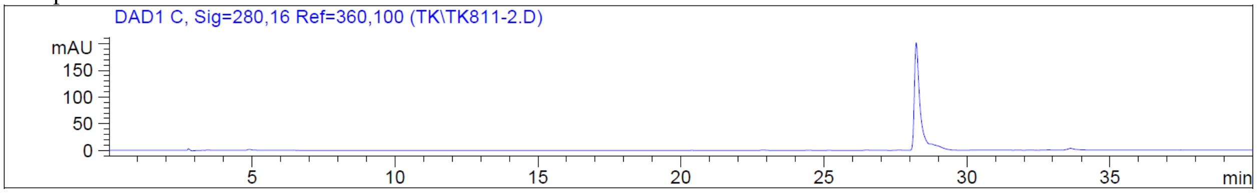

Compound **14v**

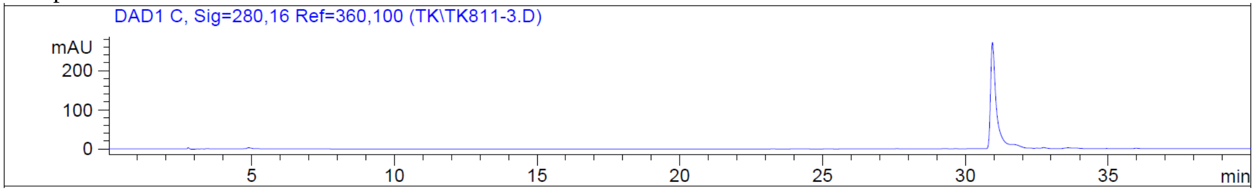

Compound **14w**

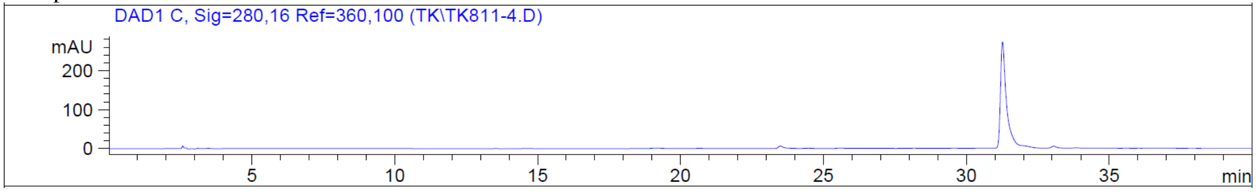

Compound **14x**

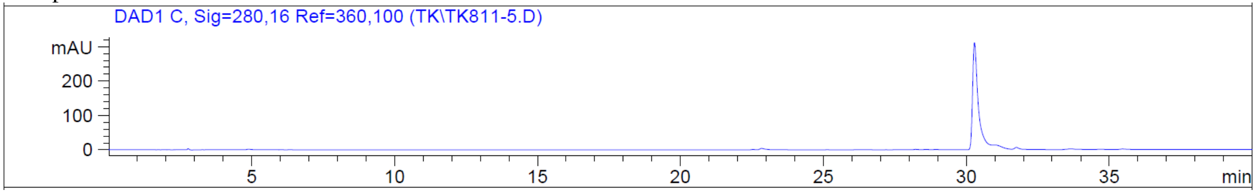

References

1. Raj, G.V.; Sareddy, G.R.; Ma, S.; Lee, T.-K.; Viswanadhapalli, S.; Li, R.; Liu, X.; Murakami, S.; Chen, C.C.; Lee, W.R.; Mann, M,; Krishnan, S.R.; Manandhar, B.;, Gonugunta, V.K.; Strand, D.; Tekmal, R.R.; Ahn, J.-M.; Vadlamudi, R.K. Estrogen receptor coregulator binding modulators (ERXs) effectively target estrogen receptor positive human breast cancers. *eLife* **2017**, *6*, e26857.
2. Ravindranathan, P.; Lee, T.-K.; Yang, L.; Centenera, M.M.; Butler, L.; Tilley, W.D.; Hsieh, J.T.; Ahn, J.-M.; Raj, G.V. Peptidomimetic targeting of critical androgen receptor-coregulator interactions in prostate cancer. *Nat. Commun*. **2013**, *4*, 1923.
